# Lymph Node Ratio as a Prognostic Factor in Neck Dissection in Oral Cancer Patients: A Systematic Review and Meta-Analysis

**DOI:** 10.3390/cancers14184456

**Published:** 2022-09-14

**Authors:** Zoi Gartagani, Stergios Doumas, Artemis Kyriakopoulou, Panagiota Economopoulou, Theodora Psaltopoulou, Ioannis Kotsantis, Theodoros N. Sergentanis, Amanda Psyrri

**Affiliations:** 1 Department of Clinical Therapeutics, “Alexandra” Hospital, School of Medicine, National and Kapodistrian University of Athens, 11528 Athens, Greece; 2 East Kent Hospitals University NHS Foundation Trust, Kent CT1 3NG, UK; 3 Department of Internal Medicine, Section of Medical Oncology, National and Kapodistrian University of Athens, Attikon University Hospital, 12462 Athens, Greece; 4 Department of Public Health Policy, School of Public Health, University of West Attica, 12243 Athens, Greece

**Keywords:** oral squamous cell carcinoma (OSCC), lymph node ratio (LNR), lymph node density, neck dissection, lymph node yield (LNY), survival

## Abstract

**Simple Summary:**

Lymph node ratio (LNR) is a well-studied prognostic factor in colorectal and breast cancer, and it has been recently evaluated as a clinically relevant biomarker in oral squamous cell carcinoma. LNR represents the ratio of positive lymph nodes extracted in a neck dissection to the total number of nodes harvested (lymph node yield, LNY). Many single-center cohort studies and a few multicenter have assessed the significance of LNR as a prognostic factor in oral cancer. In this systematic review and meta-analysis of 32 studies and 20,994 oral cancer patients, we demonstrate that LNR is an independent prognostic indicator in patients with oral squamous cell carcinoma.

**Abstract:**

Many studies have evaluated the clinical implications of lymph node ratio (LNR) as a prognostic factor in patients with oral squamous cell carcinoma (OSCC). The main purpose of this systematic review and meta-analysis was to address LNR as a prognosticator in patients with OSCC. A systematic search was conducted in the following databases: PubMed, EMBASE, Google Scholar, OpenGrey, Cochrane library, and ClinicalTrials.gov, and studies between 2009 and 2020 were sought. The pooled relative risk was calculated along with 95% confidence intervals for the following endpoints: overall survival (OS), disease-free survival (DFS), disease-specific survival (DSS), distant metastasis-free survival (DMFS), locoregional disease-free survival (LRDFS), local recurrence-free survival (LRFS), and recurrence-free survival (RFS) according to the random-effects model (Der Simonian–Laird approach). Subgroup and meta-regression analyses were performed as well. Finally, 32 cohort studies were eligible, which included 20,994 patients with OSCC. Patients were subdivided into two categories, group YES (studies that included in their analysis only patients with positive lymph nodes) and group NO (studies that did not exclude LNR = 0 patients). In the group YES, patients with high LNR had shorter OS (RR = 1.68, 95% CI: 1.47–1.91), DFS (RR = 1.68, 95% CI: 1.42–1.99), DSS (RR = 1.94, 95% CI: 1.56–2.42), DMFS (RR = 1.83, 95% CI: 1.13–2.96), LRDFS (RR = 1.55, 95% CI: 1.10–2.20), and LRFS (RR = 1.73, 95% CI: 1.41–2.13) compared to patients with low LNR. In the group NO, patients with high LNR in comparison had shorter OS (RR = 2.38, 95% CI: 1.99–2.85), DFS (RR = 2.04, 95% CI: 1.48–2.81), and DSS (RR = 2.90, 95% CI: 2.35–3.57) compared to patients with low LNR. Based on those findings, LNR might be an independent prognostic factor for OS in patients with OSCC and could be incorporated into future classification systems for better risk stratification.

## 1. Introduction

Oral cavity cancer is an emerging health problem worldwide, with a constantly increasing incidence rate and a clear male predominance [1]. The most common type of oral cancer is squamous cell carcinoma (OSCC), which accounts for nearly 90% of all oral carcinomas and is etiologically associated with tobacco exposure and increased alcohol consumption [2]. The risk of OSCC increases along with the patient’s age, with a mean age of occurrence at 58.4 years. Tongue cancer represents the most common primary tumor subsite [3].

Lymph node metastasis is the strongest prognostic factor in OSCC, and neck involvement is typically associated with poor prognosis [4,5]. The most commonly used staging system for head and neck cancer is the 8th edition (2017) of the American Joint Committee on Cancer (AJCC) Tumor Node Metastasis (TNM) Classification. The 8th edition has introduced many changes to oral cavity cancer staging. More specifically, it has incorporated high-risk pathological characteristics, such as extranodal extension (ENE) and depth of invasion (DOI), aiming for a more accurate patients’ risk stratification [6] and resulting in upstaging in many cases [7]. Depth of invasion (DOI) is defined as the distance from an adjacent normal mucosal line to the deepest point of cancer cells invasion [8] and extranodal extension (ENE) as the lymph node metastasis, which is extended beyond the capsule and can infiltrate the surrounding stromal tissue with or without stromal reaction [9].

Lymph node ratio or lymph node density (LNR, LND) is defined as the ratio of positive lymph nodes to the total number of lymph nodes excised [10]. It is a well-described prognostic factor in colorectal [11] and breast cancer [12,13], and research during the last decade has also focused on OSCC. In a few multicenter studies [14,15,16,17] and in many more single-center ones, LNR has been evaluated as a prognostic factor in patients with OSCC, as it provides fundamental information regarding the lymph node status and the extent of neck dissection [18]. In this background, the goal of our systematic review and meta-analysis was to evaluate LNR as a prognostic indicator in OSCC.

## 2. Materials and Methods

### 2.1. Protocol

The present systematic review and meta-analysis were performed following the Preferred Reporting Items for Systematic Reviews and Meta-Analyses (PRISMA) guidelines [19]. The PRISMA Statement consists of a 27-item checklist, presented in Supplementary Table S1, that includes items essential for transparent reporting of a systematic review. The objectives and methods were prespecified in a study protocol to eliminate the likelihood of biased post hoc decisions. Our study protocol was designed and agreed upon by all authors and submitted to PROSPERO International Prospective Register of Systematic Reviews (ID:318693, https://www.crd.york.ac.uk/prospero/#myprospero (accessed on 16 March 2022)). The study included patients with oral cavity cancer who had undergone neck dissection. Lymph node ratio was associated with overall survival (OS), disease-specific survival (DSS), disease-free survival (DFS), recurrence-free survival (RFS), locoregional disease-free survival (LRDFS), local recurrence-free survival (LRFS), and distant metastasis-free survival (DMFS). The study protocol is presented in Supplementary Table S2.

### 2.2. Search Strategy and Eligibility of Studies

A systematic search was conducted in PubMed, EMBASE, Google Scholar (first 1000 hits), OpenGrey, Cochrane library, and ClinicalTrials.gov and revealed a total of 2155 studies (end of search date: 20 December 2020). Our search algorithm in PubMed was the following: ((node OR nodal) AND (ratio OR density)) AND oral AND (carcinoma OR carcinomas OR cancer OR cancers OR neoplasm OR neoplasms OR malignant OR malignancy) AND (Prognosis OR Prognostic OR Outcome OR fatal OR OS OR mortality OR fatality OR death OR survival OR PFS OR DFS OR DSS OR progression OR TTP OR EFS OR recurrence OR LRF). We applied an extensive searching algorithm in order to maximize the number of studies to be scrutinized, aiming to uncover any hidden information.

Eligible studies were case-control, cohort, observational studies (retrospective cohort studies), and experimental studies (RCTs and non-RCTs) investigating the association between survival and high vs. low LNR. Case series, case reports, reviews, and meta-analyses were excluded from the analysis. All studies included patients with cancer of the oral cavity that had undergone neck dissection, and the survival data should have been presented by measurements of the LNR as categorical and not as a continuous variable. Pre-operative radiation or chemotherapy was not allowed. In the case of multiple publications from the same group of authors, articles were checked for overlapping patient pools among studies to avoid the introduction of bias by multiple data entries. In such cases, the largest sample size was chosen. The selection of studies was performed by two independent reviewers (ZG and SD), and any discrepancies were resolved upon consultation and discussion with a senior author (TNS).

### 2.3. Data Collection and Effect Estimates

Collection of data included general information about the study (first author’s name, year of publication), study characteristics (type of study, time period, geographical region, sample size, median age of patients, percentage of males, LNR cut-off value that each study used for risk stratification of patients, TNM classification, type of therapy, type of neck dissection, lymph node yield (total number of extracted lymph nodes), median number of positive nodes removed, median follow-up period), and the definition of endpoints, as well as adjustment factors in case of multivariate analysis. If the required data were not available, the reviewers contacted via e-mail the corresponding authors twice (and a reminder was sent seven days following initial contact). This policy also applied to the case of Kim KY et al. (2017) [10], where we did not receive any answer from the authors, and therefore the aforementioned study was excluded from our analysis.

Data from the eligible articles were collected and imported into a predeveloped data extraction sheet using Excel software by two independent reviewers (Z.G. and A.K.). The datasets were cross-checked, and discrepancies were discussed with the senior author (TNS).

All eligible studies were cohort studies, either prospective or retrospective, and the maximally adjusted effect estimates, relative risk (RR) or hazard ratio (HR) with their confidence intervals, were extracted from each study by category of LNR (high vs. low). A RR or HR of >1 indicated a worse prognosis. When more than two LNR cut-off values were present in a study, only the lowest cut-off was taken into consideration in the analysis. If the adjusted estimate was not available, by the provision of the number of patients under each LNR category and survival data, crude effect estimates (relative risk, RR) were calculated, and 95% CIs using 2 × 2 tables.

### 2.4. Statistical Analyses

LNR was analyzed as a dichotomous categorical variable according to the cut-off that each primary study used. The two comparison groups were patients with oral cancer who had undergone a neck dissection with a high LNR vs. those with low LNR. Statistical analyses included pooling of studies as well as meta-regression and subgroup analyses. When HRs were calculated in the primary studies using both the univariate and the multivariate model, the adjusted HRs were used in order to reduce confounding. In the eligible studies, random-effects models (Der Simonian–Laird approach) were used to calculate pooled effect estimates. Between-study heterogeneity was assessed by estimating I^2^, where I^2^ > 50% indicated substantial heterogeneity, as well as using the Mantel–Haenszel Q-test [20], where also a *p*-value < 0.05 indicated significant heterogeneity.

To facilitate our analysis, we divided the studies into two groups, group YES and group NO, depending on whether the primary studies excluded or not the patients with negative lymph nodes and LNR = 0. Hence, group A studies excluded from analysis patients with LNR = 0, group B studies included patients who could have either negative or positive lymph nodes, whereas patients with LNR = 0 belonged to the low-LNR groups.

Moreover, subgroup analyses were performed based on LNR cut-off value when two or more studies were paired under each category. In order to assess whether LNR can be characterized as an independent prognostic factor, subgroup analyses by the degree of adjustment (multivariate versus univariate analysis) under each survival outcome were also performed. The research came to the conclusion that LNR can be characterized as an independent prognostic factor, as it was proven significant in the subgroup analysis of studies performing the multivariate adjustment.

Meta-regression analysis was performed in cases of 10 or more pooled study arms [20] and aimed to assess whether gender (expressed as a 10% increase in the percentage of males in the individual studies), age (expressed as a 10-year increase in mean age), percentage of each oral cancer subsite (lip, gum, buccal mucosa, tongue, alveolus, retromolar trigone, gingiva, hard palate, and floor of mouth, expressed as a 10% increase), percentage of radical dissection (expressed as a 10% increase), percentage of extracapsular spread (per 10% increase), percentage of positive margins (per 10% increase), percentage of administered radiotherapy (per 10% increase), percentage of administered chemotherapy (per 10% increase), the median number of nodes removed (per 1 node increase), the median number of positive nodes removed (per 1 positive node increase), and the publication year (per 1-year increase) modified the association between higher LNR values and worse prognosis. Meta-regression analysis examined the quantitative influence of study characteristics on the effect size (pooled RR/HR) and allowed authors to examine the contribution of different variables to the heterogeneity in study findings.

Statistical analysis and meta-regression analysis were performed using STATA/SE version 13 (Stata Corp, College Station, TX, USA).

### 2.5. Assessment of Study Quality and Publication Bias

Regarding the risk of bias, the Newcastle–Ottawa Quality scale was used to evaluate the quality of the included non-randomized studies [21]. Regarding the items assessing the adequacy of follow-up of cohorts and whether the follow-up period was enough for outcomes to occur, the cut-off values were set a priori at 90% response rate and 2 years, respectively. Study quality was considered “low” when the Newcastle–Ottawa score (NOS) ranged between 1 and 3, “intermediate” for studies with NOS between 4 and 6, and “high” for those with a score between 7 and 9. Two independently working reviewers (ZG, AK) rated the studies, and, in case of disagreement, the final decision was reached after consultation with a senior author (TNS) and team consensus.

Publication bias was evaluated in the analyses that included 10 or more study arms. Egger’s statistical test was implemented as well as a visual inspection of the funnel plot for asymmetry. For the interpretation of Egger’s test, statistical significance was defined as *p* < 0.1. The evaluation of publication bias was performed using STATA/SE version 13 (Stata Corp, College Station, TX, USA).

## 3. Results

### 3.1. Description of Eligible Studies

**Figure 1:**
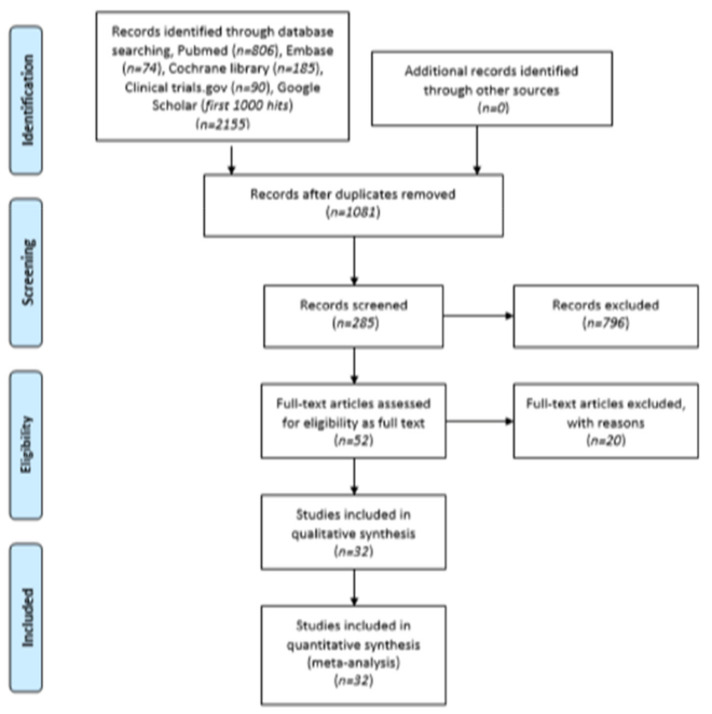
PRISMA flow diagram.

A total of 2155 studies were identified (806 from PubMed, 74 from EMBASE, 185 from Cochrane Library, and 90 from ClinicalTrials.gov) using the search algorithm. The first 1000 hits of Google Scholar were also screened. No relevant reports of unpublished literature were identified through OpenGrey. After duplicates were removed, out of 1081 records, 796 titles were considered irrelevant, and finally, 285 abstracts were screened. Reference lists of reviews and eligible articles were also systematically searched for relevant articles in a “snowball” procedure. In total, 233 were excluded as irrelevant to the topic. Fifty-two full-text articles were retrieved and assessed for eligibility, with the justified exclusion after critical appraisal of the full-text publications, of 20 articles for not meeting the eligibility criteria, data overlap or missing data, and insufficient analysis. The flow chart presenting the successive steps in the selection of eligible studies is provided in Figure 1. The studies excluded are presented in detail alongside the reasons for exclusion in Supplementary Table S3 [5,10,22,23,24,25,26,27,28,29,30,31,32,33,34,35,36,37,38,39]. Thirty-two studies were finally included in the qualitative and quantitative synthesis of our meta-analysis.

### 3.2. Study Characteristics

The main characteristics of the eligible studies are presented in Table 1, while the remaining data are presented in Supplementary Tables S4 and S5. The included articles were published between 2009 and 2020. All studies were cohort studies, with the vast majority being retrospective and only three with a prospective design: Son et al. (2017) [40], Suzuki et al. (2016) [41], and Bharath et al. (2018) [42]. There was only one multicontinental study, with 11 centers worldwide, conducted by Patel et al. (2013) [15]. Seventeen studies took place in Asia and the remaining in Europe, USA or Canada, and Australia. The sample size ranged between 35 and 4254, with a total number of 20,994 patients from all studies. The median age was between 47 and 70 years across studies. The majority of patients were male. Median follow-up ranged from 20 to 89 months.

Most studies investigated the relationship of LNR with survival in oral cancer patients, without focusing on one oral cancer subsite, except for cases that reported survival outcomes for cancer of the tongue, Bharath et al. (2018) [42], Iftikhar et al. (2020) [43], Lieng et al. (2016) [44], and Ong et al. (2016) [18], and only one study for cancer of the buccal mucosa, Chow et al. (2017) [45]. The median lymph node yield (LNY) varied between 19 and 42.5 number of total nodes removed in a neck dissection, and the median positive nodes removed from 0 to 3.4. The LNR cut-off points used in the studies ranged from 0.012 to 0.2, and the values were mainly determined via ROC-curve analysis or according to previously published literature. The most frequent outcome measured was OS (overall survival), followed by DSS (disease-specific survival), DFS (disease-free survival), LRDFS (locoregional disease-free survival), DMFS (distant metastasis disease-free survival), LRFS (local recurrence free-survival), and RFS (recurrence free-survival).

**Table 1:** Main characteristics of the eligible studies.

Study	Number of Patients	Age, Mean (Range)	Oral Cancer Subsite	Median Follow-Up, (Range)	Endpoints	LNR Cut-Off and Method of Determination	Median Nodes Removed (Lymph Node Yield, LNY)
Agarwal et al. (2019) [46]	94	47, (24–80)	Lip, buccal mucosa, tongue, alveolus, retromolar trigone	66.5 mo, (7–80)	OS, DFS	0.12 (log-rank test)	NR
Arun et al. (2020) [47]	212	52, (21–85)	NR	23.2 mo	DFS, OS	0.04 (median)	42.5
Bharath et al. (2018) [42]	51	NR	Tongue	24 mo, (24–36)	DFS, OS	0.05 (previous literature)	23.16
Chang et al. (2018) [48]	389	51.8, (23–84)	Lip, retromolar trigone, gingiva, tongue, hard palate, buccal mucosa, floor of mouth	42 mo, (0–152)	OS, DFS	0.05 (ROC curve)	NR
Chow et al. (2017) [45]	39	70, (46–95)	Buccal mucosa	79 mo, (5–167)	OS, DSS	0.07 (previous literature)	23
Ding et al. (2019) [49]	149	59, (28–88)	Tongue, floor of mouth, other	20 mo, (0–137),	OS, DFS, LRDFS, DMFS	0.1 (median)	29
Ebrahimi et al. (2011) [50]	313	63.4, (28.5–91.5)	Tongue, floor of mouth, alveolus, retromolar trigone, buccal, other	32.3 mo	OS, DSS	0.025 (log scale)	27.4
Gil et al. (2009) [51]	386	58, (14–88)	Tongue, floor of mouth, upper gum, lower gum, hard palate, retromolar trigone, buccal mucosa	67 mo, (4–184)	OS, DSS, LRDFS	0.06 (median)	35
Hosni et al. (2017) [52]	914	61, (18–92)	Tongue, others	51 mo, (1–189)	RF, DF, OS	0.06 (maximally selected rank statistic)	36
Iftikhar et al. (2020) [43]	130	High ratio: 48.3, Low ratio: 50.2	Tongue	NR	OS, DFS	0.012 (ROC curve)	NR
Jin et al. (2020) [53]	233	59.24	Tongue, non-tongue	68 mo, (1–122)	OS	0.024 (X-tile software calculation)	21.97
Kim et al. (2011) [54]	211	55, (21–88)	Tongue, floor of mouth, buccal mucosa, gingiva, hard palate, retromolar trigone	58 mo, (4–180)	DSS, OS	0.06 (previous literature)	25
Künzel et al. (2014) [55]	374	55, (26–85)	Tongue, floor of mouth, cheek, gingiva	3.99 y (0.01–24.04)	DSS, OS, LRC, LC, RC	0.05, 0.07 (ROC curve, median)	26
Lee C.C. et al. (2015) [56]	347	56	Buccal mucosa, tongue, other	33 mo	OS	0.2 (previous literature)	23.2
Lee C.C. et al. (2017) [14]	3958	59	Tongue, lip, floor of mouth, gum and retromolar trigone, buccal mucosa, hard palate, other	NR	DSS, OS	0.2 (previous literature)	33
Lee H. et al. (2019) [57]	345	55	Tongue, floor of mouth, buccal mucosa, gingiva, hard palate, retromolar trigone, lip	58 mo	DFS, OS, DSS	0.1 (ROC curve)	35
Lieng et al. (2016) [44]	72	60, (24–89)	Tongue	55 mo, (2.1–177)	DFS, OS	0.143 (log-rank test)	19
Moratin et al. (2020) [58]	430	63.9, (18–92)	Tongue, buccal mucosa, floor of mouth, alveolar process, maxilla, soft palate	NR	OS, PFS	0.08 (ROC curve)	NR
Ong et al. (2016) [18]	99	62, (23–94)	Tongue	48.5 mo, (2–156)	OS, DSS	0.06 (previous literature)	33
Patel et al. (2013) [15]	4254	52.63, (14–99)	NR	41 mo, (2–322),	OS, DSS, DFS, LRFS, LRDFS, DMFS	0.07 (ROC curve)	39
Rempel et al. (2018) [59]	171	56.6, (24–81)	Floor of mouth, tongue, mandibula/alveolar process, maxilla/ hard palate, soft palate, buccal mucosa	80.5 mo	OS	0.07 (previous literature)	22
Safi et al. (2017) [60]	499	62.51, (28–98)	Floor of mouth, tongue, lower jaw, palate, cheek	35 mo, (3–117)	LRR	0.07 (ROC curve)	20
Shrime et al. (2009) [61]	143	58.7, (14.8–89.4)	Tongue, upper and lower gingiva, floor of mouth, hard palate, buccal mucosa, retromolar trigone	32.4 mo, (1.2–140.4)	OS	0.06 (maximally selected rank statistic)	36
Son et al. (2017) [40]	157	54, (24–87)	Tongue, floor of mouth, buccal mucosa, gingiva, lip, hard palate, retromolar trigone	46 mo, (14–74)	RFS, DSS, OS	0.05 (ROC curve)	NR
Spoerl et al. (2020) [16]	717	60.8, (28–91)	Buccal mucosa, upper alveolus and gingiva, lower alveolus and gingiva, hard palate, tongue, floor of mouth	89 mo	OS, RFS	0.055 (median)	38
Subramaniam et al. (2019) [62]	643	55.1, (18–82)	Tongue, floor of mouth, buccal cavity, alveolus/retromolar trigone	2.9 years, (0.5–11)	DFS, OS	0.1 (previous literature)	23
Suzuki et al. (2016) [41]	35	NR	Tongue, upper gum, lower gum, floor of mouth, cheek mucosa, hard palate	20.9 mo	OS, DMFS, Lung MFS	0.07 (previous literature)	NR
Urban et al. (2013) [17]	3091	60, (14–99)	Tongue, floor of mouth, gum and other	21 mo	OS, CSS	0.065 (previous literature)	27
Weckx et al. (2019) [63]	159	62	Floor of mouth, tongue, lower jaw, upper jaw and hard palate, soft palate, cheek	43 mo, (3–408)	OS	0.07 (NR)	NR
Xu et al. (2017) [64]	2036	59	Tongue, lower gingiva, buccal mucosa, floor of mouth, upper gingiva, hard palate	65 mo, (1–178)	DFS, DSS	0.06 (previous literature)	23.5
Yamagata et al. (2019) [65]	95	65.5, (35–88)	Tongue, lower gingiva, floor of mouth, buccal mucosa, hard palate, upper gingiva	65.5, (35–88)	OS	0.04 (ROC curve)	33
Zhao et al. (2020) [66]	248	55.4, (26–75)	Tongue, gingiva, buccal mucosa, palate, floor of mouth, retromolar trigone	55.4, (26–75)	OS, DFS, DSS, LRFS, DMFS	0.076 (ROC curve)	32.02

### 3.3. Meta-Analysis

Overall, 32 studies were eligible for this meta-analysis, and 20,994 patients were included. Results are shown in Table 2. Studies were stratified into two groups depending on whether they included only patients with positive lymph nodes (group YES) or both patients with positive or negative lymph nodes (group NO). Some studies belonged to both group YES and group NO, as they provided analyses for both cases. In nine studies [14,18,48,49,52,53,57,62,63], the results of the multivariate analysis compared high versus low LNR patients by using LNR = 0 as the reference group in their analysis. In order to surpass this challenge, we had to calculate crude effect estimates so that the two comparison groups could remain the same (high vs. low LNR patients).

#### 3.3.1. Studies Analyzing Exclusively Patients with Positive Lymph Nodes (Group YES)

**Figure 2:**
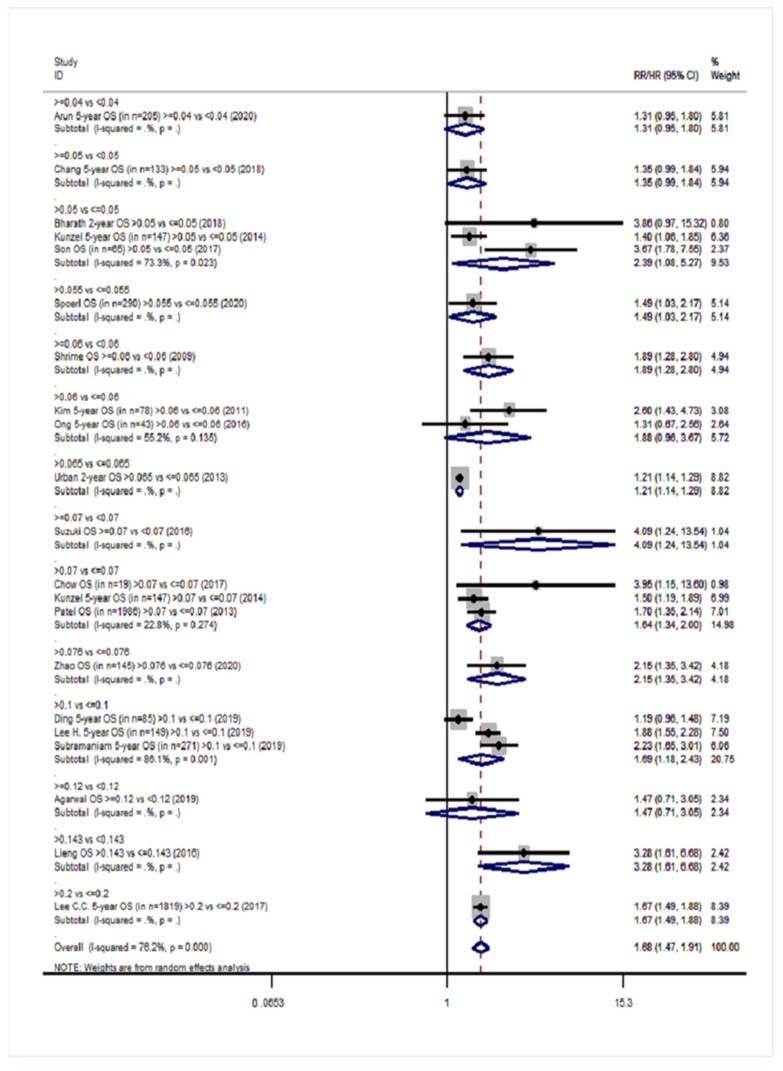
Forest plot describing the associations between lymph node ratio (LNR) and overall survival (OS) in group YES. Apart from the overall analysis, the subanalyses by LNR cut-off values are presented.

**Figure 3:**
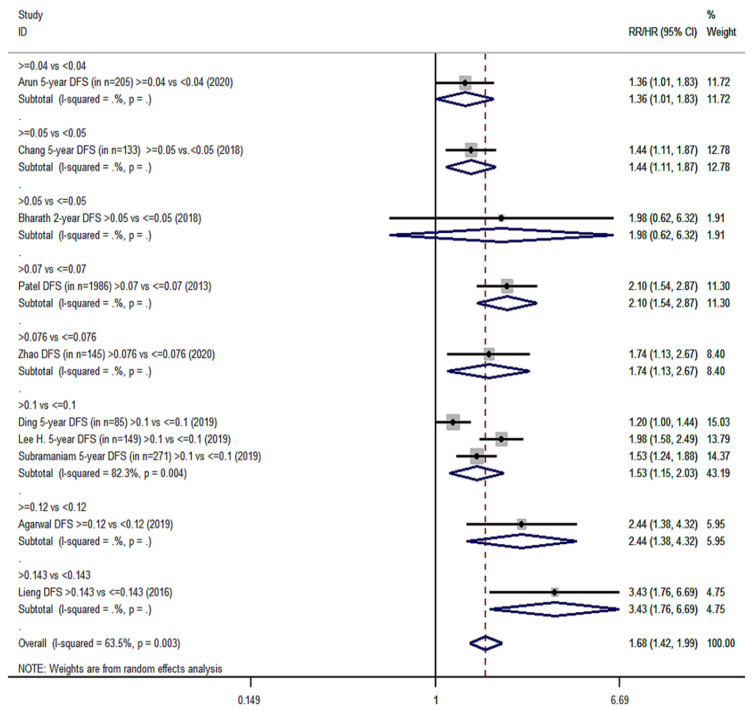
Forest plot demonstrating the associations between lymph node ratio (LNR) and disease-free survival (DFS) in group YES. Apart from the overall analysis, the subanalyses by LNR cut-off values are presented.

**Figure 4:**
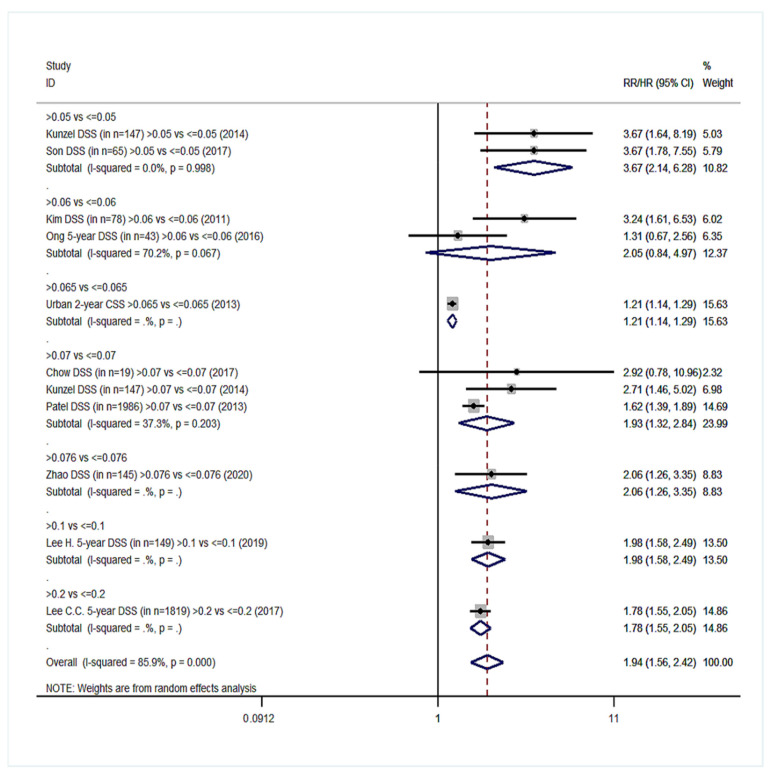
Forest plot demonstrating the associations between lymph node ratio (LNR) and disease-specific survival (DSS) in group YES. Apart from the overall analysis, the subanalyses by LNR cut-off values are presented.

**Figure 5:**
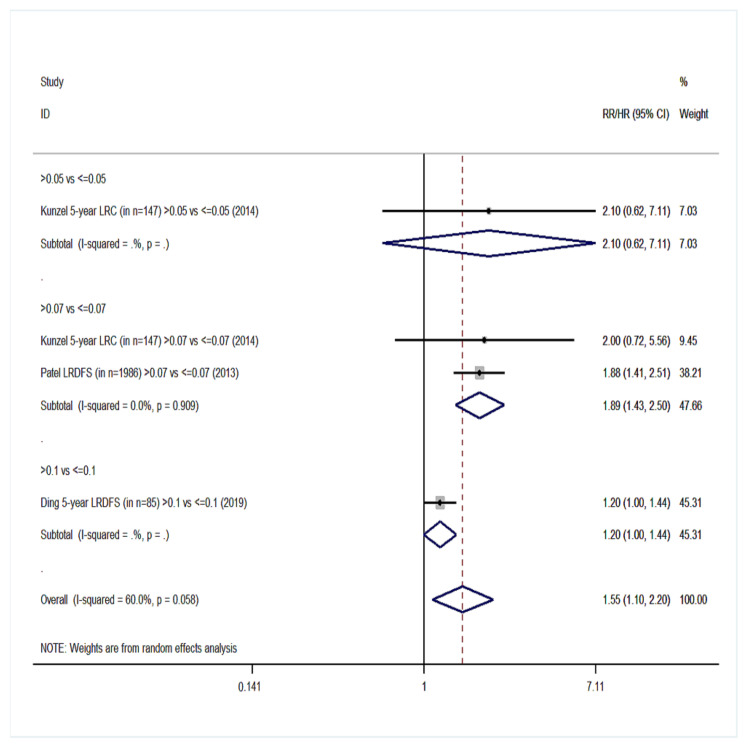
Forest plot demonstrating the associations between lymph node ratio (LNR) and locoregional disease-free survival (LRDFS) in group YES. Apart from the overall analysis, the subanalyses by LNR cut-off values are presented.

**Figure 6:**
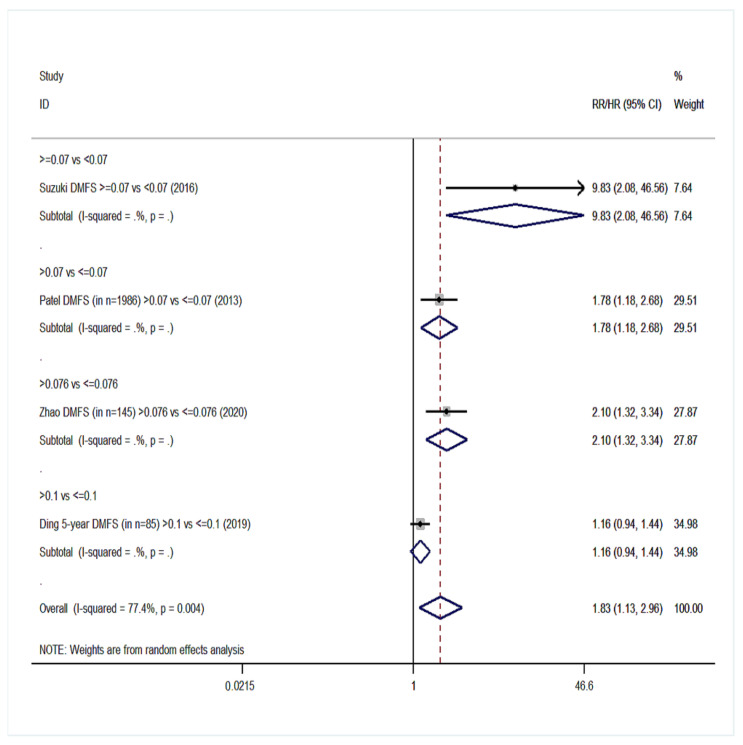
Forest plot demonstrating associations between lymph node ratio (LNR) and distant metastasis-free survival (DMFS) in group YES. Apart from the overall analysis, the subanalyses by LNR cut-off values are presented.

**Figure 7:**
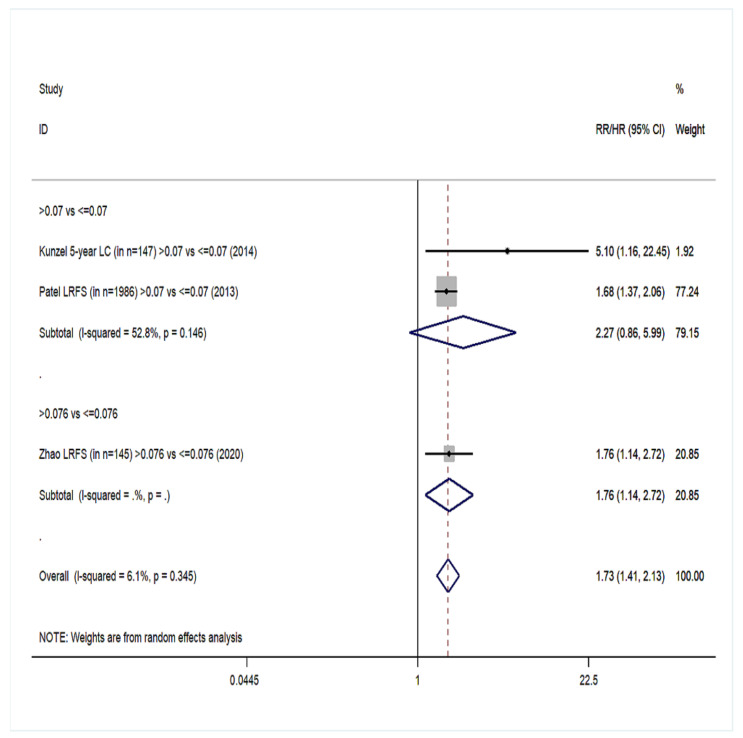
Forest plot showing the associations between lymph node ratio (LNR) and local recurrence-free survival (LRFS) in group YES. Apart from the overall analysis, the subanalyses by LNR cut-off values are presented.

Twenty cohort studies of the group YES were included in our meta-analysis with a total number of 15,281 patients [14,15,16,17,18,40,41,42,44,45,46,47,48,49,54,55,57,61,62,66]. Overall survival (OS) was the primary endpoint in all studies. Patients with high LNR values had 68% higher probability of death (Figure 2) than patients with low LNR (pooled RR = 1.68, 95% CI: 1.47–1.91) with significant heterogeneity (I^2^ = 76.2%, *p* < 0.001). For DFS, 10 studies were included (Figure 3) and patients with high LNR had a 68% increased risk for worse DFS (pooled RR = 1.68, 95% CI: 1.42–1.99) with significant heterogeneity as well (I^2^ = 63.5%, *p* = 0.003). Regarding DSS, data from 11 studies showed that patients with high LNR have a 94% increased risk (Figure 4) compared with patients with low LNR (pooled RR = 1.94, 95% CI: 1.56–2.42, I^2^ = 85.9%, *p* < 0.001). Similarly, for LRDFS, data from four studies demonstrated that patients with high LNR have a 55% increased probability of locoregional disease recurrence (Figure 5) than patients with low LNR (pooled RR = 1.55, 95% CI: 1.10–2.20, I^2^ = 60%, *p* = 0.058). For DMFS, data from four studies showed that patients with high LNR had an 83% increased risk of distant metastasis (Figure 6) compared with patients with low LNR (pooled RR = 1.83, 95% CI: 1.13–2.96, I^2^ = 77.4%, *p* = 0.004). In addition, combination of data from three studies showed a 73% increased risk for local recurrence (pooled RR = 1.73, 95% CI: 1.41–2.13, I^2^ = 6.1%, *p* = 0.345, Figure 7). Regarding RFS, analysis of two studies did not show any significant association (pooled RR = 2.27, 95% CI: 0.91–5.62, I^2^ = 80.4%, *p*= 0.024, Supplementary Figure S1).

#### 3.3.2. Studies Analyzing Patients with Positive and Negative Lymph Nodes (Group NO)

**Figure 8:**
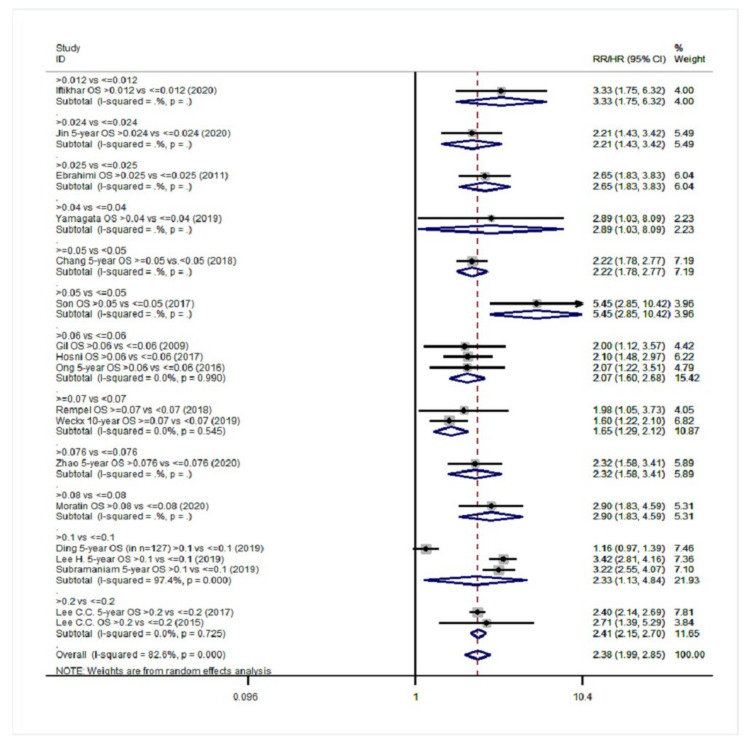
Forest plot showing associations between lymph node ratio (LNR) and overall survival (OS) in group NO. Apart from the overall analysis, the subanalyses by LNR cut-off values are presented.

**Figure 9:**
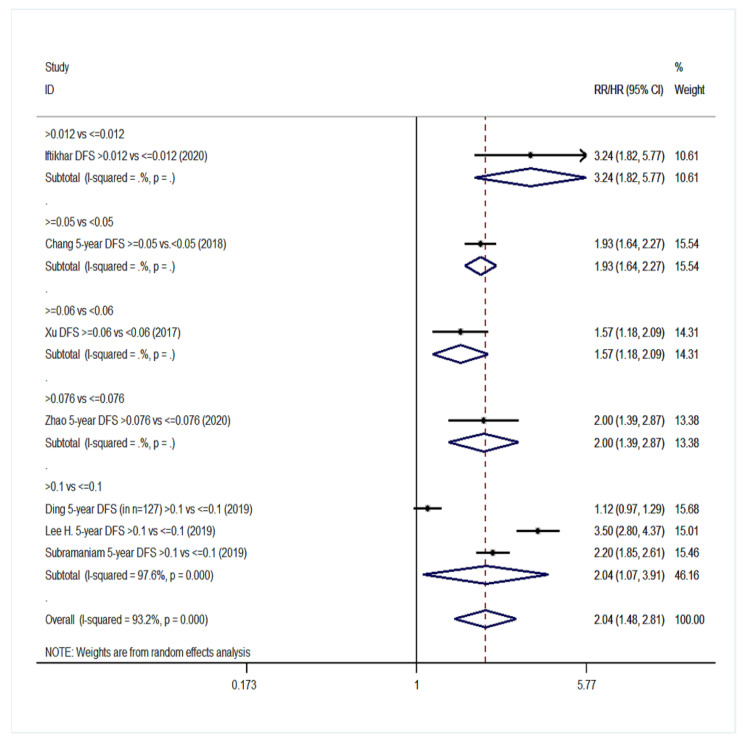
Forest plot describing the association between lymph node ratio (LNR) and disease-free survival (DFS) in group NO. Apart from the overall analysis, the subanalyses by LNR cut-off values are presented.

**Figure 10:**
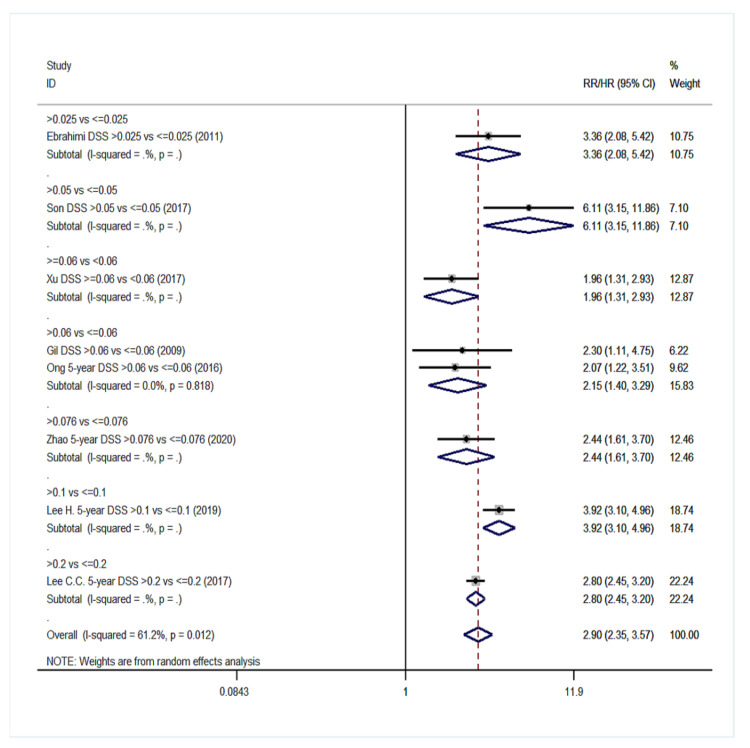
Forest plot demonstrating associations between lymph node ratio (LNR) and disease-specific survival (DSS) in group NO. Apart from the overall analysis, the subanalyses by LNR cut-off values are presented.

Overall, 20 studies were eligible for meta-analysis in the group NO with a total of 11,701 patients [14,18,40,43,48,49,51,52,53,56,57,58,59,60,62,63,64,65,66,67]. Eighteen studies were included in the statistical analysis for OS. Patients with high LNR values had a 138% increased probability of death (Figure 8) compared to patients with low LNR values (pooled RR = 2.38; 95% CI: 1.99–2.85). Considerable heterogeneity existed among the studies for OS (I^2^ = 82.6%, *p* < 0.001). Pooling of seven studies also exhibited a burdening effect of higher LNR values on DFS (pooled RR = 2.04; 95% CI: 1.48–2.81, I^2^: 93.2%, *p* < 0.001, Figure 9). Pooled analysis of eight studies on DSS indicated a pooled relative risk of 2.90 (95% CI: 2.35–3.57, I^2^: 61.2%, *p* = 0.012, Figure 10). Regarding LRDFS and DMFS, combination of three study arms for each outcome resulted in a relative risk greater than 1 (pooled RR = 1.88 and pooled RR = 2.11, respectively) but without statistically significant associations (95% CI: 0.83–4.25 for LRDFS and 95% CI: 0.97–4.63 for DMFS, Supplementary Figures S2 and S3).

### 3.4. Meta-Regression Analysis

Supplementary Table S6 presents the results of the meta-regression analysis.

In group YES, the floor of mouth and tongue cancer modified the correlation between LNR and OS. Cancer on the floor of the mouth was found to slightly modify the association between OS and LNR (exponentiated coefficient: 0.89, 95% CI: 0.79–0.99, *p* = 0.033); more specifically, high LNR patients and carcinomas located on the floor of the mouth experienced better prognosis compared to other oral cancer patients with high LNR values as well. On the contrary, cancer of the tongue had the opposite effect on the association between OS and LNR (exponentiated coefficient: 1.11, 95% CI: 1.04–1.19, *p* = 0.004); patients with high LNR and cancer of the tongue have less favorable survival. The bubble plot for tongue cancer is presented in Supplementary Figure S4, and the bubble plot for cancer on the floor of the mouth is presented in Supplementary Figure S5. In group NO, higher percentage of tumors located in the tongue was the only variable that could modify the correlation between LNR and OS of patients (exponentiated coefficient: 1.08; 95% CI: 1.01–1.16, *p* = 0.032). The bubble plot (Supplementary Figure S6) shows that the effect of high LNR values was more pronounced in terms of OS in studies with increased number of patients with tongue tumors.

### 3.5. Evaluation of Quality of Studies and Risk of Bias

Within-study risk of bias assessment for all 32 studies included in the systematic review with the Newcastle–Ottawa Scale is presented in detail in Supplementary Table S7. Twenty-four studies were found to be of high quality, while the remaining belonged in the “intermediate” range. All studies have excellent scores in the selection process, and follow-up was adequate (≥90% response rate) in the majority of studies. In terms of comparability, pN-classification was considered the most significant confounding factor. Only six studies were adjusted on pN-classification, and generally, the overall quality was compromised in the “comparability” section.

In the group YES, significant publication bias was detected via Egger’s test in the analysis of OS (*p* = 0.001) and DSS (*p* = 0.003). These results are reflected as asymmetry in the respective funnel plots (Supplementary Figures S7 and S8). On the contrary, no significant publication bias was detected via Egger’s test in the analysis of DFS (*p* = 0.076), and the funnel plot of DFS showed no significant asymmetry (Supplementary Figure S9). Regarding publication bias in group NO, no significant publication bias was detected via Egger’s test in the analysis on OS (*p* = 0.572); the result was reflected in the respective funnel plot, as no obvious asymmetry was identified (Supplementary Figure S10).

### 3.6. LNR as an Independent Prognostic Factor

#### 3.6.1. LNR as an Independent Prognostic Factor in Group YES

In group YES, the pooled estimate was calculated separately for studies with multivariate (pooled RR = 1.90, 95% CI: 1.64–2.21) and univariate (pooled RR = 1.68, 95% CI: 1.47–1.91) analyses, and both results were statistically significant for OS (Supplementary Figure S11). For DFS, the pooled RRs were 2.07 (95% CI: 1.77–2.42) and 1.68 (95% CI: 1.42–1.99), respectively (Supplementary Figure S12). For DSS, the results from the meta-analysis were statistically significant for multivariate (pooled RR = 2.21, 95% CI: 1.75–2.80) and univariate analysis (pooled RR = 1.94, 95% CI: 1.56–2.42) (Supplementary Figure S13). The results for DMFS, LRDFS, LRFS, and RFS are presented in Supplementary Figures S14–S17.

#### 3.6.2. LNR as an Independent Prognostic Factor in Group NO

In group NO (Supplementary Figures S18–S22), when adjusting for potential confounders, patients with high LNR values had a twofold or more risk of worse prognosis, (pooled RR for OS = 2.82; 95% CI: 2.36–3.37, pooled RR for DFS = 2.58; 95% CI: 1.44–4.64, pooled RR for DSS = 3.23; 95% CI: 2.25–4.64, pooled RR for LRDFS = 2.92; 95% CI: 1.41–6.03). Regarding studies that did not adjust for potential confounding factors, the results from the analysis were the following: pooled RR for OS = 2.06, 95% CI: 1.59–2.67, pooled RR for DFS = 1.74, 95% CI: 1.22–2.48, pooled RR for DSS = 2.72, 95% CI: 2.40–3.08. Results regarding univariate analysis in LRDFS and DMFS lacked statistical significance (pooled RR for LRDFS = 1.12; 95% CI: 0.97–1.29, pooled RR for DMFS = 2.11; 95% CI: 0.97–4.63).

## 4. Discussion

The present systematic review and meta-analysis of 32 studies assessed the relationship between LNR and survival outcomes in patients with oral squamous cell carcinoma who had undergone neck dissection. Importantly, we show that high LNR is significantly correlated with a worse prognosis.

Our results are in accordance with a recent meta-analysis by Huang et al. (2019) [67], which included 19 studies. Huang et al. showed that LNR is a prognostic factor in oral cancer patients for OS, DFS, and DSS. Our study provides a more thorough insight into this reported relationship between LNR and survival; we studied a larger compendium of endpoints (OS, DFS, DSS, DMFS, LRDFS, LRFS, and RFS), and we pooled data from a total of 32 studies. We also performed subgroup and meta-regression analyses to explore potential effect modifiers and address the arising issue of heterogeneity between studies. In addition, we evaluated LNR as an independent prognostic factor by performing subgroup analyses for studies with multivariate as opposed to univariate analysis.

During the past few years, many studies have demonstrated the value of LNR as a better prognostic factor compared to N status proposed by the 8th edition of AJCC guidelines for TNM status for oral cancer patients [68]. This could be explained by the fact that LNR reflects not only the N status but also the extent of the disease. A possible challenge could relate to cases where the primary lesion crosses the midline, and bilateral neck dissection is necessary. Another possible challenge could relate to the N3 status during pathological examination of the surgical specimens, where the presence of a large positive solitary node >6 cm could not be easily differentiated from the coalescence of multiple lymph nodes, and because of that rationale, some studies excluded N3 node status from their analysis [18,42,54]. The aforementioned challenges make the use of LNR as a prognostic factor difficult in those cases, as it is a fraction, and it depends on the alterations that its numerator, the positive lymph nodes excised, and its denominator, the lymph node yield, incur. Subsequently, LNR having the LNY as its denominator should not lead the surgeon to perform more extended neck dissections, if not necessary, in order to increase the LNY and decrease the total fraction of the LNR. Kim et al. (2011) reported that in N+ patients, limited neck dissections did not affect the prognostic ability of LNR [54]. Therefore, care must be taken to the adequacy of the neck dissection performed and to the meticulous examination of the specimen rather than the extension of the neck dissection to a higher number of levels [69].

The total number of lymph nodes retrieved from neck dissection surgery (LNY) depends on the type, as well as the quality of the neck dissection per se. A retrospective study from Ebrahimi et al. (2011) [70], analyzing 225 patients with oral cancer who had undergone a supraomohyoid elective neck dissection (SOHND), showed that an LNY of more than 18 was linked to a more favorable prognosis. This finding proposed by Ebrahimi et al. (2011) was further validated from two prospective NRG Oncology trials (Radiation Therapy Oncology Group RTOG 9501 and RTOG 0234), reporting an improvement in overall survival and a decrease in loco-regional failure when 18 was used as a threshold in LNY [71]. Naturally, LNY is higher in cases of modified radical or radical dissections, where frequently, the desirable LNY is greater than 30 [72]. However, selective neck dissection (SND) does not apply to all oral cancer subsites. For instance, in tongue or floor of mouth cancers, more radical procedures are the preferred approach, as these tumors are associated with skip lymph node metastases, with the previous node level free of metastatic disease. Therefore, in tongue and floor of mouth cancers, modified radical dissection still remains the standard of care for an LN-positive (LN+) neck [73]. Another new technique used in N0 patients is sentinel lymph node biopsy (SLNB), aiming to minimize the use of more radical procedures, where unnecessary, and their impact on patients’ morbidity and economic burden [74].

To further explore the heterogeneity of results, we performed a meta-regression on variables that are considered potential modifiers of the association between LNR and overall survival. The only three potential confounding factors that slightly modified the relationship between LNR and survival were the floor of the mouth as the primary site in group YES and the tongue as the primary site in both groups, as shown in the results of the meta-regression analysis. This finding can be explained by the fact that patients with floor-of-mouth cancer are typically more likely to develop cervical lymph node metastases [36], and therefore, LNR might not be the most ideal prognostic factor for these patients, as they may be categorized as high LNR patients early at their course of the disease. Patients with anterior tongue tumors are diagnosed with occult neck metastases in 50–60% of cases, even in early T1/T2 stages, and occult neck metastases can increase the risk of dying from cancer by five times [75]. Disease progression might be quicker in tongue cancer compared to other sites due to the complexity of the tongue’s lymphatic and vascular network [76].

The present systematic review and meta-analysis has several limitations. First, all eligible studies were observational studies, and only three of them were prospectively designed. In addition, many studies did not report patient and disease characteristics, such as personal history, comorbidities, post-operative therapy administered, lymph node yield, and other pathological features. Another important limitation is heterogeneity in reporting pathological outcomes across the studies; for instance, surgical specimens were assessed by general pathologists or technicians in the majority of the studies, except for the study by Agarwal et al. (2019) [46], where pathologists trained in head and neck cancer evaluated the nodal biopsies. Marres et al. (2014) retrospectively studied all the neck dissection specimens in their institution between 2002 and 2012. Before 2007 the specimens were examined by pathologists, and after 2007 by pathology technicians. Their study showed that after 2007 the mean LNY increased from 24 to 32, and alongside the mean LNR decreased from 11.4% to 8.7% [77]. This finding makes apparent the fact that the implementation of a standardized protocol for harvesting and examining ND specimens is necessary. To address this problem, the American Association of Pathologists proposed a detailed form of reporting for head and neck cancer in order to minimize the potential reporting biases and lack of reporting on important cancer features such as positive margins of resection and extranodal extension [78].

There are some limitations in the general applicability of LNR as a prognostic factor in patients with OSCC. Firstly, it is dependent on the extent of the neck dissection performed, as when more radical procedures are performed, the lymph node yield increases. Another difficulty arising in the implementation of LNR is the variability of cut-off points used in the literature. In the studies included in our meta-analysis, LNR cut-offs ranged from 0.012 to 0.2, with a mean LNR cut-off point of 0.068. Patel et al. (2013) [15] retrieved data from the ICOR database and included the largest pool of patients, with a total of 4254 patients. Using the ROC curve, they identified 0.07 as a validated cut-off, and it was used as a point of reference in some studies [41,45,59]. Other cut-off points that were frequently used in the eligible studies were 0.06 [18,51,52,54,61,64] and 0.05 [40,42,48,55]. Hence, the cut-off points that seem to have a greater consensus in the literature vary between 0.05 and 0.07, although a universal LNR cut-off point remains to be identified.

Despite the limitations, our study has a number of strengths. Literature was meticulously searched, and every effort possible was made to explore and reduce heterogeneity. Additionally, our subanalyses adjusted for co-variates highlighted LNR as an independent prognostic factor. More prospective studies with clearly defined endpoints and clinical trials with large sample sizes will help further validate these findings, establish a universal cut-off for each surgical procedure, and might incorporate LNR in future classification systems as an important prognostic factor for patients with oral cancer.

## 5. Conclusions

In conclusion, our systematic review and meta-analysis showed that LNR is an independent prognostic factor for OS for patients with oral cancer who had undergone neck dissection independently of LN status. Patients with high LNR are linked with significantly worse survival outcomes compared with patients with low LNR values for almost all studied survival endpoints, although tongue and FOM carcinomas have a slight tendency to modify the relationship between LNR and survival. We can safely come to the conclusion that LNR is a reliable prognostic factor combining various information, such as N status, the extent of the disease, and the radicality or not of the neck dissection performed, and therefore, LNR could possibly contribute to better risk stratification of oral cancer patients, adding valuable information in the existing classification system. We believe that more prospective, well-designed studies are needed to validate the significance and reproducibility of LNR as a prognostic factor.

## Figures and Tables

**Table 2:** Correlations between lymph node ratio (LNR) and survival outcomes; subgroup analyses by LNR cut-off values are presented. Significant associations are noted in bold.

Survival Endpoints	Studies Analyzing Exclusively Patients with Positive Lymph Nodes (Group YES)			Studies Analyzing Patients with Positive and Negative Lymph Nodes (Group NO)		
	n ^§^	RR (95% CI)	Heterogeneity I^2^, *p*	n ^§^	RR (95% CI)	Heterogeneity I^2^, *p*
Overall survival (OS)	20	**1.68 (1.47–1.91)**	76.2%, <0.001	18	**2.38 (1.99–2.85)**	82.6%, <0.001
Disease-free survival (DFS)	10	**1.68 (1.42–1.99)**	63.5%, 0.003	7	**2.04 (1.48–2.81)**	93.2%, <0.001
Disease-specific survival (DSS)	11	**1.94 (1.56–2.42)**	85.9%, <0.001	8	**2.90 (2.35–3.57)**	61.2%, 0.012
Recurrence-free survival (RFS)	2	2.27 (0.91–5.62)	80.4%, 0.024	1	Only 1 study	NC
Locoregional disease-free survival (LRDFS)	4	**1.55 (1.10–2.20)**	60%, 0.058	3	1.88 (0.83–4.25)	72.4%, 0.027
Distant metastasis-free survival (DMFS)	4	**1.83 (1.13–2.96)**	77.4%, 0.004	3	2.11 (0.97–4.63)	94%, <0.001
Local recurrence-free survival (LRFS)	3	**1.73 (1.41–2.13)**	6.1%, 0.345	1	Only 1 study	NC

^§^ number of studies; RR: relative risk.

## Supplementary Materials

The following supporting information can be downloaded at: https://www.mdpi.com/article/10.3390/cancers14184456/s1, Table S1. The Preferred Reporting Items for Systematic Reviews and Meta-Analyses (PRISMA) Checklist [79], Table S2. Study protocol, Table S3. Excluded studies, with reasons, Table S4. Characteristics of the eligible studies, Table S5. Characteristics of the eligible studies, Table S6. Meta-regression analysis examining the role of potential modifiers in the association between lymph node ratio (LNR) and survival outcomes, Table S7. Evaluation of within-study risk of bias with the Newcastle–Ottawa Scale, Figure S1. Forest plot describing the association between lymph node ratio (LNR) and recurrence-free survival in group YES. Apart from the overall analysis, the subanalyses by LNR cut-off values are presented, Figure S2. Forest plot describing the association between lymph node ratio (LNR) and locoregional disease-free survival in group NO. Apart from the overall analysis, the subanalyses by LNR cut-off values are presented, Figure S3. Forest plot describing the association between lymph node ratio (LNR) and distant metastasis-free survival in group NO. Apart from the overall analysis, the subanalyses by LNR cut-off values are presented, Figure S4. Plot depicting the modifying effect mediated by percentage of tumors affecting the tongue upon the association between high lymph node ratio (LNR) values and overall survival in group YES. The circle sizes represent the inverse of each within-study variance, Figure S5. Plot depicting the modifying effect mediated by percentage of tumors affecting the floor of mouth upon the association between high lymph node ratio (LNR) values and overall survival in group YES. The circle sizes represent the inverse of each within-study variance, Figure S6. Plot depicting the modifying effect mediated by percentage of tumors affecting the tongue upon the association between high lymph node ratio (LNR) values and overall survival in group NO. The circle sizes represent the inverse of each within-study variance, Figure S7. Funnel plot of the meta-analysis on overall survival in group YES showing evidence of publication bias as considerable asymmetry, Figure S8. Funnel plot of the meta-analysis on disease-specific survival in group YES showing evidence of publication bias as considerable asymmetry, Figure S9. Funnel plot of the meta-analysis on disease-free survival in group YES without obvious asymmetry, i.e., no evidence of publication bias, Figure S10. Funnel plot of the meta-analysis on overall survival in group NO without obvious asymmetry, i.e., no evidence of publication bias, Figure S11. Forest plot describing the association between lymph node ratio (LNR) and overall survival in group YES. Apart from the overall analysis, the subanalyses on degree of adjustment are presented, Figure S12. Forest plot describing the association between lymph node ratio (LNR) and disease-free survival in group YES. Apart from the overall analysis, the subanalyses on degree of adjustment are presented, Figure S13. Forest plot describing the association between lymph node ratio (LNR) and disease-specific survival in group YES. Apart from the overall analysis, the subanalyses on degree of adjustment are presented, Figure S14. Forest plot describing the association between lymph node ratio (LNR) and distant metastasis-free survival in group YES. Apart from the overall analysis, the subanalyses on degree of adjustment are presented, Figure S15. Forest plot describing the association between lymph node ratio (LNR) and locoregional disease-free survival in group YES. Apart from the overall analysis, the subanalyses on degree of adjustment are presented, Figure S16. Forest plot describing the association between lymph node ratio (LNR) and local recurrence-free survival in group YES. Apart from the overall analysis, the subanalyses on degree of adjustment are presented, Figure S17. Forest plot describing the association between lymph node ratio (LNR) and recurrence-free survival in group YES. Apart from the overall analysis, the subanalyses on degree of adjustment are presented, Figure S18. Forest plot describing the association between lymph node ratio (LNR) and overall survival in group NO. Apart from the overall analysis, the subanalyses on degree of adjustment are presented, Figure S19. Forest plot describing the association between lymph node ratio (LNR) and disease-free survival in group NO. Apart from the overall analysis, the subanalyses on degree of adjustment are presented, Figure S20. Forest plot describing the association between lymph node ratio (LNR) and disease-specific survival in group NO. Apart from the overall analysis, the subanalyses on degree of adjustment are presented, Figure S21. Forest plot describing the association between lymph node ratio (LNR) and locoregional disease-free survival in group NO. Apart from the overall analysis, the subanalyses on degree of adjustment are presented, Figure S22. Forest plot describing the association between lymph node ratio (LNR) and distant metastasis-free survival in group NO. Apart from the overall analysis, the subanalyses on degree of adjustment are presented.
