# Supplementary material for: Lymph Node Ratio as a Prognostic Factor in Neck Dissection in Oral Cancer Patients: A Systematic Review and Meta-Analysis
Source: Cancers (Basel). 2022 Sep 14;14(18):4456. doi: 10.3390/cancers14184456 (PMC9497248; doi:10.3390/cancers14184456)
Supplement: Supplementary file 1 [file cancers-14-04456-s001.zip › cancers-1911959-supplementary.pdf]

Supplementary Material

Table S1. The Preferred Reporting Items for Systematic Reviews and Meta-Analyses (PRISMA) Checklist (continued) [1].

Section/topic	#	Checklist item	Reported on page #
**TITLE**			
Title	1	Identify the report as a systematic review, meta-analysis, or both.	p.1
**ABSTRACT**			
Structured summary	2	Provide a structured summary including, as applicable: background; objectives; data sources; study eligibility criteria, participants, and interventions; study appraisal and synthesis methods; results; limitations; conclusions and implications of key findings; systematic review registration number.	p. 1
**INTRODUCTION**			
Rationale	3	Describe the rationale for the review in the context of what is already known.	p. 2
Objectives	4	Provide an explicit statement of questions being addressed with reference to participants, interventions, comparisons, outcomes, and study design (PICOS).	p. 2
**METHODS**			
Protocol and registration	5	Indicate if a review protocol exists, if and where it can be accessed (e.g., Web address), and, if available, provide registration information including registration number.	p.2, Table S2
Eligibility criteria	6	Specify study characteristics (e.g., PICOS, length of follow-up) and report characteristics (e.g., years considered, language, publication status) used as criteria for eligibility, giving rationale.	p. 2-3
Information sources	7	Describe all information sources (e.g., databases with dates of coverage, contact with study authors to identify additional studies) in the search and date last searched.	p. 3
Search	8	Present full electronic search strategy for at least one database, including any limits used, such that it could be repeated.	p. 3
Study selection	9	State the process for selecting studies (i.e., screening, eligibility, included in systematic review, and, if	p. 3

		applicable, included in the meta-analysis).	
Data collection process	10	Describe method of data extraction from reports (e.g., piloted forms, independently, in duplicate) and any processes for obtaining and confirming data from investigators.	p. 3
Data items	11	List and define all variables for which data were sought (e.g., PICOS, funding sources) and any assumptions and simplifications made.	p. 3
Risk of bias in individual studies	12	Describe methods used for assessing risk of bias of individual studies (including specification of whether this was done at the study or outcome level), and how this information is to be used in any data synthesis.	p. 4-5
Summary measures	13	State the principal summary measures (e.g., risk ratio, difference in means).	p. 3-4
Synthesis of results	14	Describe the methods of handling data and combining results of studies, if done, including measures of consistency (e.g., I^2) for each meta-analysis.	p. 4

Section/topic	#	Checklist item	Reported on page #
Risk of bias across studies	15	Specify any assessment of risk of bias that may affect the cumulative evidence (e.g., publication bias, selective reporting within studies).	p. 5
Additional analyses	16	Describe methods of additional analyses (e.g., sensitivity or subgroup analyses, meta-regression), if done, indicating which were pre-specified.	p. 4
**RESULTS**			
Study selection	17	Give numbers of studies screened, assessed for eligibility, and included in the review, with reasons for exclusions at each stage, ideally with a flow diagram.	p. 5-6, Figure 1, Table S3
Study characteristics	18	For each study, present characteristics for which data were extracted (e.g., study size, PICOS, follow-up period) and provide the citations.	p. 6-7, Tables 1, S4, S5
Risk of bias within studies	19	Present data on risk of bias of each study and, if available, any outcome level assessment (see item 12).	p.4, 18, Table S7

Results of individual studies	20	For all outcomes considered (benefits or harms), present, for each study: (a) simple summary data for each intervention group (b) effect estimates and confidence intervals, ideally with a forest plot.	p. 10-17, Figures 2-10, Figures S1-S3
Synthesis of results	21	Present results of each meta-analysis done, including confidence intervals and measures of consistency.	p. 10-17, Table 2, Figures 2-10, Figures S1-S3
Risk of bias across studies	22	Present results of any assessment of risk of bias across studies (see Item 15).	p. 18, Figures S7-S10
Additional analysis	23	Give results of additional analyses, if done (e.g., sensitivity or subgroup analyses, meta-regression [see Item 16]).	p. 18-19, Table S6, Figures S4-S6, S11-S22
**DISCUSSION**			
Summary of evidence	24	Summarize the main findings including the strength of evidence for each main outcome; consider their relevance to key groups (e.g., healthcare providers, users, and policy makers).	p. 19-20
Limitations	25	Discuss limitations at study and outcome level (e.g., risk of bias), and at review-level (e.g., incomplete retrieval of identified research, reporting bias).	p. 20
Conclusions	26	Provide a general interpretation of the results in the context of other evidence, and implications for future research.	p. 19-20
**FUNDING**			
Funding	27	Describe sources of funding for the systematic review and other support (e.g., supply of data); role of funders for the systematic review.	-

Table S2. Study protocol (continued).

**Title**	Lymph node ratio as a prognostic factor in neck dissection in oral cancer patients: A systematic review and meta-analysis.
**PICOS**	
**Population**	Oral cancer patients undergone neck dissection
**Intervention**	Neck dissection (Selective, Modified Radical, Radical, Bilateral)
**Comparison**	Lymph node ratio (high vs. low)
**Outcomes**	Survival outcomes (overall survival, disease-free survival, disease-specific survival, locoregional disease-free survival, distant metastasis-free survival, recurrence-free survival, local recurrence-free survival)
**Study Design**	Observational studies (case-control, prospective cohort, retrospective cohort), experimental studies (RCTs, non-RCTs)
**Eligibility Criteria**	**1.** Observational studies (case-control, prospective cohort, retrospective cohort), experimental studies (RCTs, non-RCTs).<br>**2.** Each study should report that all patients have oral cancer and a neck dissection surgery is performed.<br>**3.** Survival data should be reported by measurements of the lymph node ratio as a dichotomous categorical variable.
**Exclusion Criteria**	**1.** Case reports, case series, reviews and meta-analyses.<br>**2.** Evidence of preoperative administration of radiotherapy and/or chemotherapy.
**Languages**	English (or foreign in case of possibility of translation)
**Databases**	PubMed, Google Scholar, Cochrane Library, EMBASE, ClinicalTrials.gov, OpenGrey
**End of Search Date**	20 December 2020
**PubMed Search Strategy**	((node OR nodal) AND (ratio OR density)) AND oral AND (carcinoma OR carcinomas OR cancer OR cancers OR neoplasm OR neoplasms OR malignant OR malignancy) AND (Prognosis OR Prognostic OR Outcome OR fatal OR OS OR mortality OR fatality OR death OR survival OR PFS OR DFS OR DSS OR progression OR TTP OR EFS OR recurrence OR LRF)<br><br>There were no limits applied to the search and no language restriction.The search

	algorithm was rather broad, so as to maximize the number of articles to be scrutinized, aiming to uncover any hidden (i.e. not apparent in the abstract) information in the full text and tables of articles.

Table S3. Excluded studies, with reasons (continued).

Author	Year	PMID	Reasons for exclusion
Adel et al. [2]	2016	27057838	Data overlap with paper including similar analysis from ICOR database (Patel et al., 2013)
Amar et al. [3]	2012	22714852	Insufficient data analysis with no hazard ratios (HRs) provided as effect estimate
Chen et al. [4]	2015	26302761	No separate analysis for oral cancer
Faisal et al. [5]	2020	33236214	Insufficient data, multivariate analysis with LNR=0 as reference and the number of patients

			under each LNR category was not provided
Feng et al. [6]	2017	28751709	No separate analysis for oral cancer
Hingsammer et al. [7]	2019	30738712	Multivariate analysis with insufficient survival data and no HRs
Iocca et al. [8]	2020	32380357	Oral cancer as reference group in multivariate analysis and no association investigated between LNR and survival
Kim KY et al. [9]	2012	22193423	Data overlap with more recent paper by the same author (2017)
Kim KY et al. [10]	2017	27588367	Insufficient data, multivariate analysis with LNR=0 as reference and the number of patients under each LNR category was not provided - Unsuccessful attempts to contact the authors

Liao et al. [11]	2012	22104249	Data overlap with paper including similar analysis from ICOR database (Patel et al., 2013) and use of multiple LNR cut-off points according to neck dissection levels
Mascitti et al. [12]	2018	30217459	Insufficient data analysis with no HRs and 95% CIs provided
Noble et al. [13]	2016	26851040	Insufficient data analysis with no 95% CIs provided in the multivariate analysis and absence of Kaplan - Meier curves for LNR
Roberts et al. [14]	2016	26969807	No separate analysis for oral cancer
Safi et al. [15]	2017	28981183	Patient data included in a larger cohort investigated around the same time (Safi et al., 2017)
Safi et al. [16]	2017	28529103	Patient data included in a larger cohort investigated around the same time (Safi et al.,

			2017)
Safi et al. [17]	2018	29709331	Patient data included in a larger cohort investigated around the same time (Safi et al., 2017)
Sayed et al. [18]	2013	23893514	Data overlap with paper including similar analysis from ICOR database (Patel et al., 2013)
Shrime et al. [19]	2009	19340867	Includes patients who received preoperative radiation
Troeltzsch et al. [20]	2018	30098956	LNR is investigated as a continuous variable
Zirk et al. [21]	2018	29249633	Data overlap with study investigating a larger cohort (Safi et al., 2017)

Table S4. Characteristics of the eligible studies (continued).

Study	Study period	Study design	Number and percentage of males	Geographical region	Ethnicity	Definition of endpoints	TNM stage (AJCC)	T stage	N stage	Treatment groups
Agarwal et al. (2019) [22]	2011	Retrospective cohort	70, (74%)	India/Pakistan	NR	OS: date of treatment completion to date of death/last follow-up DFS: NR	NR	T1: 6 (6%), T2: 7 (8%), T3: 13 (14%), T4: 68 (72%)	N1: 1 (1%), N2a: 28 (30%), N2b: 3 (3%), N2c: 1 (1%), N3b: 61 (65%)	Surgery + RT: 19 (20.2%), Surgery + CRT: 75 (79.8%)
Arun et al. (2020) [23]	2011-2016	Retrospective cohort	153, (72.2%)	India/Pakistan	NR	DFS: time between the date of primary surgery and the date of clinicoradiological confirmation of disease recurrence (locoregional or distant metastasis)/last follow-up OS: time between the date of primary surgery for OSCC and the date of death of any cause/last follow-up	NR	T1: 30 (14.2%), T2: 79 (37.3%), T3: 23 (10.8%), T4a: 63 (29.7%), T4b: 17 (8%)	N1: 83 (39.2%), N2a: 3 (1.4%), N2b: 102 (48.1%), N2c: 20 (9.4%), N3: 4 (1.9%)	Surgery alone: 19/205 (9.3%), Surgery + RT: 81/205 (39.5%), Surgery + CRT: 105 (51.2%)
Bharath et al. (2018) [24]	2012-2013	Prospective cohort	39 (78.6%)	India/Pakistan	NR	NR	NR	T1: 14 (33.3%), T2: 29 (50.9%), T3: 6 (11.7%), T4: 2 (3.9%)	N1: 24 (47%), N2: 27 (53%)	Surgery alone: 2 (3.9%), Surgery + RT: 49 (96.1%), Surgery + CRT: 15 (29.4%)
Chang et al. (2018) [25]	2002-2015	Retrospective cohort	355 (91.3%)	East Asia	Eastern Asian	OS: day of therapeutic	I: 99 (25.4%), II: 85 (21.9%), III:	T1: 119 (30.6%), T2: 125 (32.1%),	N0: 256 (65.8%), N1: 55 (14.1%),	Surgery alone: 106,

						surgery to date of death/last follow-up<br>DFS: day of surgery to date of tumor recurrence (local/distant metastasis)	64 (16.5%), IV: 141 (36.2%)	T3: 43 (11.1%), T4: 102 (26.2%)	N2a: 2 (0.5%), N2b: 64 (16.5%), N2c: 11 (2.8%), N3: 1 (0.3%)	Surgery + RT: 69, Surgery + CT: 56, Surgery + CCRT: 158
Chow et al. (2017) [26]	2000-2016	Retrospective cohort	20 (51.3%)	East Asia	Eastern Asian	NR	I: 5(12.8%), II: 11 (28.2%), III: 10 (25.6%), IV: 13 (33.3%)	T1: 9 (23.1%), T2: 15 (38.5%), T3: 7 (17.9%), T4: 8 (20.5%)	N0: 20 (51.3%), N1: 10 (25.6%), N2: 9 (23.1%), N3: 0	Surgery alone: 21 (53.8%), surgery+ RT: 11 (28.2%), Surgery+ CT: 1 (2.6%), Surgery+ CRT: 6 (15.4%)
Ding et al. (2019) [27]	2000-2015	Retrospective cohort	105 (70.5%)	USA/Canada	White: 117 (78.5%), Non-white: 32 (21.5%)	OS: date of diagnosis to date of death/last follow-up<br>DFS: date of diagnosis to date of local recurrence/regional lymph node metastasis/distant metastasis<br>LRDFS: date of diagnosis to date of local recurrence/regional lymph node metastasis<br>DMFS: date of diagnosis to date of distant metastasis	NR	T1: 46 (30.9%), T2: 40 (26.8%), T3-T4: 63 (42.3%)	N0: 41 (27.5%), N1:24 (16.1%), N2- N3: 62 (41.6%)	Surgery alone: 26 (17.4%), Surgery+ RT: 33 (22.1%), Surgery+ CRT: 90 (60.4%)
Ebrahimi et al. (2011) [28]	1987-2009	Retrospective cohort	204 (65.2%)	Australia	NR	OS: date of surgery to date of death/last follow-	NR	T1-T2: 198 (63.3%) T3-T4: 115	N0: 148 (47.3%), N1: 50 (16%), N2a: 6 (1.9%),	NR

						up, DSS: date of surgery to date of OSCC death		(36.7%)	N2b: 85 (27.2%), N2c: 24 (7.7%)	
Gil et al. (2009) [29]	1986-1996	Retrospective cohort	227 (59%)	USA/Canada	NR	OS: date of surgery to date of death/last follow-up, DSS: date of surgery to date of death from OSCC LRDFS: NR	I: 44 (11%), II: 103 (27%), III: 90 (23%), IV: 149 (39%)	T1: 56 (15%), T2: 168 (44%), T3: 70 (18%), T4: 92 (24%)	N0: 219 (57%), N1: 72 (19%), N2a: 2 (1%), N2b: 83 (22%), N2c: 8 (2%), N3: 2 (1%)	Surgery alone: 162 (42%), Surgery + RT: 224 (58%)
Hosni et al. (2017) [30]	1994-2012	Retrospective cohort	577 (63%)	USA/Canada	NR	OS: date of surgery to death/last follow-up RF: date of surgery to regional failure with no evidence of local failure/distant metastases ,DF: date of surgery to distant metastases with no evidence of local/regional failure	NR	T1-T2: 631 (69%), T3-T4: 283 (31%)	N0: 482 (52.7%), N1: 128 (14%), N2a: 6 (0.7%), N2b: 225 (24.6%), N2c: 73 (8%)	Surgery alone, Surgery + RT, Surgery + CRT
Iftikhar et al. (2020) [31]	2000-2018	Retrospective cohort	87 (66%)	India/Pakistan	NR	OS: deceased or alive after 5 years from primary treatment (surgery) DFS: recurrence or no recurrence after 5 years from the start of treatment	I: 34 (26.2%), II: 37 (28.5%), III: 21 (16.2%), IVA: 38 (29.2%)	T1: 44 (33.8%), T2: 69 (53.1%), T3: 11 (8.5%), T4: 6 (4.6%)	N0: 75 (57.7%), N1: 20 (15.4%), N2: 35 (26.9%)	Surgery alone: 53 (40.8%), Surgery + RT: 43 (33.1%), Surgery + CRT: 34 (26.2%)
Jin et al. (2020) [32]	2009-2013	Retrospective cohort	127 (55%)	East Asia	NR	OS: time from initial diagnosis to all-cause death	NR	T1-T2: 166 (71%), T3-T4: 67 (29%)	N0: 156 (67%), N1: 33 (14%), N2-N3: 44 (19%)	NR
Kim et al. (2011) [33]	1994-2006	Retrospective cohort	134 (64%)	East Asia	NR	First day of treatment to date	I: 85 (40%), II: 33 (16%), III: 31 (15%),	T1: 101 (48%), T2: 71 (34%), T3: 11	N0: 133 (63%), N1: 34 (16%),	Surgery alone: 135 (64%),

						of event or last follow-up	IV: 62 (29%)	(5%), T4: 28 (13%)	N2b: 37 (18%), N2c: 7 (3%)	Surgery + RT: 69 (33%), Surgery + CRT: 7 (3%)
Künzel et al. (2014) [34]	1980-2010	Retrospective cohort	297 (79.4%)	Europe	NR	OS: date of initial diagnosis to death/last follow-up DSS: date of initial diagnosis to tumor- or treatment related death/time of patient's last admission LRC: time of initial diagnosis/patient's last admission to the first locoregional recurrence LC: time of initial diagnosis/patient's last admission to the first local recurrence RC: time of initial diagnosis/patient's last admission to the first regional recurrence	I: 105 (28.1%), II: 78 (20.9%), III: 73 (19.5%), IV: 118 (31.6%)	T1: 154 (41.2%), T2: 154 (41.2%), T3: 39 (10.4%), T4: 26 (7.2%), Tx: 1 (0.2%)	N0: 209 (55.9%), N1: 58 (15.5%), N2a: 6 (1.6%), N2b: 66 (17.6%), N2c: 18 (4.8%), N3: 17 (4.5%)	Surgery alone: 95 (25.4%), Surgery + RT: 235 (62.8%), Surgery + CRT: 43 (11.5%), Surgery + CT: 1 (0.3%)
Lee C.C. et al. (2015) [35]	2004-2013	Retrospective cohort	322 (92.8%)	East Asia	NR	NR	NR	T1: 95 (27.4%), T2: 112 (32.3%), T3: 30 (8.6%), T4: 110 (31.7%)	N0: 235 (67.7%), N1: 30 (8.6%), N2: 80 (23.1%), N3: 2 (0.6%)	NR
Lee C.C. et al. (2017) [36]	2007-2013	Retrospective cohort	2528 (63.9%)	USA/Canada	White: 3316 (83.8%), Black/Other:	OS: time of initial diagnosis to death from all causes	I: 938 (23.7%), II: 694 (17.5%), III: 849 (21.5%),	T1: 1398 (35.3%), T2: 1353 (34.2%), T3: 474 (12%),	N0: 2132 (53.9%), N1: 826 (20.9%), N2: 967 (24.4%),	NR

					642 (16.2%)	DSS: time of initial diagnosis to death from cancer	IVA: 1393 (35.2%), IVB: 84 (2.1%)	T4: 733 (18.5%)	N3: 33 (0.8%)	
Lee H. et al. (2019) [37]	2006-2015	Retrospective cohort	214 (62%)	East Asia	NR	DFS: time from initial surgery to recurrence/last follow-up<br>OS: time from initial surgery to all-cause death/last follow-up<br>DSS: time from initial surgery to disease-specific death/last follow-up	I: 134 (38.8%), II: 42 (12.2%), III: 48 (13.9%), IVA: 121 (35.1%)	T1: 170 (49.3%), T2: 89 (25.8%), T3: 6 (1.7%), T4a: 80 (23.2%)	N0: 196 (56.8%), N1: 61 (17.1%), N2b: 72 (20.9%), N2c: 16 (4.6%)	Surgery alone: 190 (55.1%), Surgery + RT: 123 (35.7%), Surgery + CRT: 32 (9.3%)
Lieng et al.(2016) [38]	1980-2011	Retrospective cohort	48 (67%)	Australia	NR	DFS: time from diagnosis to time of recurrence, death or development of a second malignancy<br>OS: NR	NR	T1: 23 (32%), T2: 31 (43%), T3: 9 (12.5%), T4:9 (12.5%)	N1: 43 (60%), N2: 28 (39%), N3: 1 (1%)	Surgery alone: 19 (26%), Surgery + RT (+/- CT): 53 (74%
Moratin et al. (2020) [39]	2010-2017	Retrospective cohort	273 (63.5%)	Europe	NR	NR	I: 138 (32.1%), II: 73 (17%), III: 47 (10.9%), IV: 172 (40%)	T1: 165 (38.4%), T2: 122 (28.4%), T3: 28 (6.5%), T4: 115 (26.7%)	N0: 280 (65.1%), N1: 50 (11.6%), N2a: 3 (0.7%), N2b: 52 (12.1%), N2c: 31 (7.2%), N3a: 1 (0.2%), N3b: 10 (2.3%), Missing: 3 (0.7%)	NR
Ong et al. (2016) [40]	2002-2010	Retrospective cohort	56 (56.6%)	East Asia	NR	OS: date of surgery to date of death/last follow-up<br>DSS: date of surgery to death of	I: 25 (25.3%), II: 26 (26.3%), III: 18 (18.2%), IV: 26 (26.3%), Unknown: 4 (4%)	T1: 39 (39.3%), T2: 44 (44.4%), T3: 8 (8.1%), T4: 6 (6.1%), Unknown: 2 (2%)	N0: 57 (57.6%), N1: 17 (17.2%), N2/N3: 25 (25.2%)	Surgery alone: 65 (65.7%), Surgery + RT: 25 (25.2%), Surgery + CRT: 9 (9.1%)

						tongue cancer			
Patel et al. (2013) [41]	NR	Retrospective cohort	2815 (60.1%)	Multicontinental (11 centers worldwide)	NR	OS: date of surgery to date of death/last follow-up, DSS: time of diagnosis to death resulting from OSCC<br>DFS: NR<br>LRFS: NR<br>LRDFS: NR<br>DMFS: NR	I: 464 (9%), II: 799 (13%), III: 668 (16%), IV: 2323 (62%)	T1: 613 (13%), T2: 1374 (30%),<br>T3: 623 (15%), T4: 1644 (42%)	N0: 2268 (43.3%), N1: 652 (15.3%), N2a: 88 (2%), N2b: 988 (23.2%), N2c: 246 (6%), N3: 12 (0.2%)	Surgery alone: 1297 (22%), Surgery + RT: 2245 (58%), Surgery + CRT: 553 (15%), Surgery + RT+ Erbitux: 159 (5%)
Rempel et al. (2018) [42]	1994-2013	Retrospective cohort	129 (75%)	Europe	NR	OS: time from the beginning of primary therapy to death from any cause	II: 22 (13%), III: 24 (14%), IVA: 96 (56%), IVB: 29 (17%)	T2: 58 (34%), T3: 27 (16%), T4a: 57 (33%), T4b: 29 (17%)	N0: 34 (20%), N1: 29 (17%), N2: 106 (62%), N3: 2 (1%)	Surgery + CRT: 171 (100%)
Safi et al. (2017) [43]	2004-2014	Retrospective cohort	290 (58.1%)	Europe	NR	LRR: tumor of similar histology appearing after 6 weeks of treatment and within the first 3 years after therapy of the primary tumor locally/within the lymph neck nodes	I: 166 (33.26%), II: 116 (23.24%), III: 64 (12.82%), IV: 153 (30.68%)	T1: 206 (41.28%), T2: 166 (33.26%), T3: 39 (7.8%), T4: 88 (17.66%)	N0: 342 (68.5%), N+: 157 (31.5%)	Surgery alone: 258 (51.7%), Surgery + RT: 95 (19%), Surgery + CRT: 146 (29.3%)
Shrime et al. (2009) [44]	1994-2004	Retrospective cohort	94 (65.7%)	USA/Canada	NR	Date of diagnosis to date of death/last follow-up	NR	T1-T2: 65 (45.8%),<br>T3-T4: 77 (54.2%)	N1: 48 (33.6%), N2: 95 (66.4%)	Surgery alone: 50 (35%), Surgery + RT: 91 (63.6%), Surgery + CT: 2 (1.4%)
Son et al. (2017) [45]	2010-2015	Prospective cohort	101 (64.3%)	East Asia	NR	RFS: date of surgery to date of first recurrence<br>DSS: date of	I: 59 (37.6%),<br>II: 14 (8.9%),<br>III: 19 (12.1%), IV: 65 (41.4%)	T1: 75 (47.8%),<br>T2: 21 (13.4%),<br>T3: 4 (2.5%), T4: 57 (36.3%)	N0: 92 (58.6%), N1: 22 (14%), N2: 43 (27.4%)	Surgery alone: 78 (49.7%), Surgery + RT: 56 (35.7%),

						surgery to date of index-cancer death OS: date of surgery to date of all-cause death/last follow-up				Surgery + CRT: 23 (14.6%)
Spoerl et al. (2020) [46]	2004-2017	Retrospective cohort	515 (71.8%)	Europe	NR	OS: date of resection to date of death/last follow-up, RFS: date of resection to date of first recurrence/last follow-up	I: 219 (30.5%), II: 117 (16.3%), III: 115 (16%), IV: 266 (37.1%)	T1: 290 (40.4%), T2: 236 (32.9%), T3: 56 (7.8%), T4: 135 (18.8%)	N0: 427 (59.5%), N1: 110 (15.3%), N2a: 8 (1.1%), N2b: 113 (15.8%), N2c: 50 (7%), N3: 9 (1.3%)	Surgery alone: 382 (53.3%), Surgery + RT: 232 (32.4%), Surgery + CRT: 103 (14.4%)
Subramaniam et al. (2019) [47]	2004-2014	Retrospective cohort	498 (77%)	India/Pakistan	NR	OS: time from initial surgery to date of death/last follow-up DFS: time from initial surgery to date of recurrence (local, regional or distant)	NR	T1: 261 (41%), T2: 228 (35%), T3: 59 (9%), T4a: 95 (15%)	N0: 372 (58%), N1: 101 (15%), N2a: 10 (2%), N2b: 3 (1%), N2c: 22 (3%), N3b: 135 (21%)	Surgery alone: 301 (46%), Surgery + RT: 171 (27%), Surgery + CRT: 171 (27%)
Suzuki et al. (2016) [48]	2008-2013	Prospective cohort	22 (62.9%)	East Asia	Eastern Asian	OS: period from resection to death/last contact DMFS: period from resection to date of distant metastases/last contact, Lung MFS: period from resection to date of lung metastases/last contact	III: 4 (11.4%), IV: 31 (88.6%)	T1: 4 (11.4%), T2: 15 (42.9%), T3: 7 (20%), T4: 9 (25.7%)	N1: 5 (14.3%), N2: 29 (82.9%), N3: 1 (2.9%)	Surgery alone: 14 (40%), Surgery + RT: 10 (28.6%), Surgery + CRT: 7 (20%), Surgery + CT: 4 (11.4%)
Urban et al. (2013) [49]	1988-2007	Retrospective cohort	2021 (65%)	USA/Canada	White: 2515 (81%),	OS: time of initial diagnosis to date of	NR	T1: 766 (27%), T2: 1217 (43%),	N1: 942 (32%), N2: 1798 (61%),	Surgery alone: 747 (24%),

					Black: 320 (10%), Other: 256 (8%)	death/last follow-up, CSS: NR		T3: 857 (30%)	N3: 227 (8%)	Surgery + RT: 2344 (76%)
Weckx et al. (2019) [50]	2002-2015	Retrospective cohort	87 (55%)	Europe	NR	OS: period of time from the beginning of the primary therapy to all-cause death, in months	I: 36 (23%), II: 32 (20%), III: 18 (11%), IVA: 49 (31%), IVB: 24 (15%)	T1: 39 (25%), T2: 48 (30%), T3: 20 (13%), T4a: 44 (28%), T4b: 10 (6%)	N0: 96 (60%), N1: 21 (13%), N2a: 12 (8%), N2b: 9 (6%), N2c: 5 (3%), N3a: 0 (0%), N3b: 16 (10%)	Surgery alone: 70 (44%), Surgery + RT: 31 (20%), Surgery + CRT: 58 (37%)
Xu et al. (2017) [51]	1999-2011	Retrospective cohort	1151 (56.5%)	East Asia	NR	DFS: time from diagnosis until first documented recurrence/death DSS: time from the first operation to death/last follow-up	NR	T1: 497 (24.4%), T2: 793 (38.9%), T3: 211 (10.4%), T4a: 503 (24.7%), T4b: 32 (1.6%)	N0: 928 (45.6%), N1: 293 (14.4%), N2: 401 (19.7%), N3: 5 (0.2%), Unknown: 409 (20.1%)	Surgery alone: 1076 (52.8%), Surgery + RT: 542 (26.6%), Surgery + CCRT: 149 (7.4%), Missing: 269 (13.2%)
Yamagata et al. (2019) [52]	2008-2015	Retrospective cohort	52 (54.7%)	East Asia	NR	OS: date of first diagnosis to death from any cause	I: 17 (17.9%), II: 23 (24.2%), III: 13 (13.7%), IVA: 40 (42.1%), IVB: 2 (2.1%)	T1: 23 (24.2%), T2: 40 (42.1%), T3: 7 (7.4%), T4a: 23 (24.3%), T4b: 2 (2.2%)	N0: 43 (45.3%), N1: 22 (23.2%), N2b: 24 (25.3%), N2c: 6 (6.3%)	Surgery alone: 66 (69.5%), Surgery + RT: 4 (4.3%), Surgery + CRT: 25 (26.3%)
Zhao et al. (2020) [53]	2008-2010	Retrospective analysis from phase III RCT	172 (69.4%)	East Asia	NR	OS: date of random assignment to occurrence of all-cause death DFS: date of random assignment to tumor recurrence/all-cause death DSS: date of random	0: 11 (4.4%), I: 12 (4.8%), II: 21 (8.5%), III: 75 (30.2%), IV: 129 (52%)	T0: 15 (6.1%), T1: 35 (14.1%), T2: 71 (28.6%), T3: 90 (36.3%), T4: 37 (14.9%)	N0: 103 (41.5%), N1: 41 (16.5%), N2a: 6 (2.5%), N2b: 77 (31%), N2c: 21 (8.5%)	Surgery + RT: 127 (51.2%), Surgery + CRT: 121 (48.8%)

assignment to occurrence of OSCC death<br>LRFS: date of random assignment to local tumor/neck recurrence/all-cause death<br>DMFS: date of random assignment to tumor distant metastasis/all-cause death

Table S5. Characteristics of the included studies (continued).

Study	Number of patients per oral cancer subsite	Adjustment factors	Univariate/multivariate analysis	Type of neck dissection, patients per type, n (%)	Percentage of MRND, RND, B/L ND, n (%)	Median/mean positive nodes removed (range)	Presence of extracapsular spread, n (%)	Close /involved margins, n (%)
Agarwal et al. (2019) [22]	3 (3%), 16 (17%), 39 (42%), 34 (36%), 2 (2%)	PNI, ENE >2, ENE grade 3-4	Multivariate	Unilateral Selective: 12 (13%), Bilateral Selective: 7 (7%), Unilateral Modified radical: 49 (52%), Unilateral Modified radical + Contralateral Selective: 15 (16%), Bilateral Modified radical: 11 (12%)	U/L MND: 49 (52%), U/L MND + C/L SND: 15 (16%), B/L MND: 11 (12%)	2 (1-25)	91, (97%)	Involved: 1, (1%)
Arun et al. (2020) [23]	NR	N/A	Univariate	Unilateral: 153<br>Bilateral: 59	NR	2 (1-42)	122, (57.5%)	Close /involved: 53, (25%)
Bharath et al. (2018) [24]	51 (100%)	N/A	Univariate	NR	NR	1.98	22, (43.1%)	NR
Chang et al. (2018) [25]	2 (0.5%), 18 (4.6%), 52 (13.4%), 170 (43.7%), 9 (2.3%), 127 (32.6%), 11 (2.8%)	N/A	Univariate	NR	NR	NR	0, (0%)	0 (0%)
Chow et al. (2017) [26]	39 (100%)	N/A	Univariate	Selective<br>24 (61.5%), Modified radical 12 (30.8%), Radical<br>3 (7.7%)	MRND: 12 (30.8%), RND: 3 (7.7%)	1 (1-17)	6, (15.4%)	Involved: 6, (15.4%)
Ding et al. (2019) [27]	61 (40.9%), 43 (28.9%), 45 (30.2%)	N/A	Univariate	NR	NR	2	35, (23.5%)	Involved: 48, (32.2%)
Ebrahimi et al. (2011) [28]	109 (34.8%), 116 (37.1%), 41 (13.1%), 28 (8.9%), 15 (4.8%), 4 (1.3%)	age, T-classification, ECS, involved margin	Multivariate	Level I-V: 61 (15.2%), Level I-IV: 110 (27.4%), Level I-III: 220 (54.7%), Other: 11 (2.7%)	Level I-V: 61 (15.2%)	3.4	62, (19.8%)	Involved: 18.2%

Gil et al. (2009) [29]	175 (45%), 79 (20%), 4 (1%), 66 (17%), 2 (1%), 36 (9%), 24 (6%)	pT-, pN-classification, overall TNM stage, ECS, total no. of nodes, no. of positive nodes	Multivariate	Selective: 229 (59%), Modified radical: 65 (17%), Radical: 50 (13%), Bilateral: 46 (12%)	MRND: 65 (17%), RND: 50 (13%), B/L ND: 46 (12%)	2.7 (1-22)	24.6%	NR
Hosni et al. (2017) [30]	419 (46%), 495 (54%)	NR	Univariate/ Multivariate	Ipsilateral (all) Selective: 625 (68.4%), Modified Radical: 239 (26.1%), Radical: 21 (2.3%), Limited Upper: 29 (3.2%) Contralateral (368) Selective 277 (75.3%), Modified Radical 38 (10.3%), Limited Upper 53	Ipsilateral (all) MRND: 239 (26.1%), RND: 21 (2.3%) Contralateral (368) MRND: 38 (10.3%)	2 (1-49)	187, (20%)	Involved: 77, (8%)
Iftikhar et al. (2020) [31]	130 (100%)	Margin status	Multivariate	Ipsilateral Modified radical: 82 (68.3%), Selective: 20 (16.7%), Radical: 18 (15%) Contralateral Selective: 41 (75.9%), Modified radical: 12 (22.2%), Radical: 1 (1.85%)	Ipsilateral MRND: 82 (68.3%), RND: 18 (15%), Contralateral MRND: 12 (22.2%), RND: 1 (1.85%)	NR	NR	Involved: 12, (9.2%)
Jin et al. (2020) [32]	84 (37%), 149 (63%)	N/A	Univariate	NR	NR	0.74	NR	NR
Kim SY et al. (2011) [33]	166 (79%), 17 (8%), 16 (8%), 5 (2%), 4 (2%), 3 (1%)	tumor thickness, T-classification, No. of positive nodes, size	Multivariate	Elective: 151 (62%), Therapeutic: 60 (28%), Selective: 125 (59%), Modified Radical/Radical: 54 (26%), Bilateral: 32 (15%)	MR/R: 54 (26%), Bilateral: 32 (15%)	2 (1-17)	19, (9%)	Involved: 12, (6%)

		of metastatic deposits						
Künzel et al. (2014) [34]	218 (58.3%), 137 (39.6%), 14 (3.7%), 5 (1.3%)	pN (grouped), UICC	Multivariate/Univariate	Bilateral: 182, Ipsilateral: 192	B/L ND: 182	2 (1-15)	32, (9%)	Involved: 11, (3%)
Lee C.C. et al.(2015) [35]	158 (45.5%), 116 (33.4%), 73 (21%)	age, gender, comorbidity, pT, primary tumor site, margin status, differentiation	Multivariate	Elective: 195 Therapeutic: 152	NR	1.04	NR	Involved: 29, (8.4%)
Lee C.C. et al. (2017) [36]	2041 (51.6%), 160 (4%), 671 (17%), 680 (17.2%), 268 (6.8%), 55 (1.4%), 83 (2.1%)	N/A	Univariate	NR	NR	1.31	NR	NR
Lee H. et al. (2019) [37]	277 (80.3%), 31 (9%), 15 (4.3%), 13 (3.8%), 3 (0.9%), 4 (1.2%), 2 (0.6%)	KPS≤80, postoperative therapy, tumor site, tumor size, DOI, PNI, differentiation, involved margins, T-,N-classification, no. of positive nodes, >40 examined nodes, laterality of node involved, low neck	Multivariate	Elective/ Therapeutic	NR	0	149, (43.2%)	Involved: 20, (5.8%)

		node involvement , ENE						
Lieng et al. (2016) [38]	72 (100%)	NR	Multivariate	NR	NR	NR	33, (46%)	Involved: 7, (10%)
Moratin et al. (2020) [39]	97 (22.6%), 33 (7.7%), 120 (27.9%), 119 (27.7%), 29 (6.7%), 32 (7.4%)	T-stage, grading, age	Multivariate	NR	NR	NR	NR	Involved: 23, (5.3%)
Ong et al. (2016) [40]	99 (100%)	N/A	Univariate	Radical: 34 (34.3%), Comprehensive: 20 (20.2%), Selective (supraomohyoid): 39 (39.4%), Unknown: 6 (6.1%)	RND: 34.3%	2 (0-13)	NR	Involved: 7, (7.1%)
Patel et al. (2013) [41]	NR	gender, age, DOI, ECS, margins, T-, N-classification , TNM stage,, LND-based TNM stage, total no. of lymph nodes, treatment group	Multivariate	Elective: 2434 (52%), Therapeutic: 1820 (48%), I-III/IV: 2746 (60.7%), I-V: 525 (13.2%), Radical: 327 (9.9%), Bilateral: 656 (16%)	RND: 327 (9.9%), B/L ND: 656 (16%)	3.1 (1-34)	1280, (30%)	NR
Rempel et al. (2018) [42]	71 (42%), 32 (19%), 32 (19%), 25 (15%), 5 (3%), 6 (4%)	age, margin status, ypT-, ypN-classification	Multivariate	Modified radical: 171 (100%)	MRND: 100%	Mean:1.18 (0-18) Median: 0	NR	18, (10%)
Safi et al. (2017) [43]	158 (31.66%), 119 (23.84%), 94 (18.83%), 54 (10.82%), 74 (14.85 %)	grading, ECS, T-classification , treatment	Multivariate	Selective, Modified radical, Bilateral	NR	1 (1-11)	41, (8.2%)	0, (0%)
Shrime et al. (2009) [44]	NR	NR	Multivariate	NR	NR	3.3 (1-24)	56, (41.8%)	NR

Son et al. (2017) [45]	140 (89.2%), 4 (2.5%), 4 (2.5%), 3 (1.9%), 3 (1.9%), 2 (1.3%), 1 (0.6%)	tumor size >2 cm, close/involved margins	Multivariate	Elective: 102, Therapeutic: 55	Therapeutic: 55 (41.4%)	1.4	NR	Involved: 6, (3.8%)
Spoerl et al. (2020) [46]	51 (7.1%), 22 (3.1%), 106 (14.8%), 48 (6.7%), 210 (29.3%), 280 (39.1%)	NR	Multivariate	Unilateral: 72, Bilateral: 218	B/L ND: 218	mean 3.1 (1-41) median 2	78, (10.9%)	NR
Subramaniam et al. (2019) [47]	429 (67%), 37 (6%), 173 (26%), 4 (1%)	N/A	Univariate	Ipsilateral selective, contralateral	0%	NR	167, (26%)	Involved: 5, (1%)
Suzuki et al. (2016) [48]	20 (57.1%), 4 (11.4%), 4 (11.4%), 3 (8.6%), 3 (8.6%), 1 (2.9%)	p stage (IV/III), positive surgical margin/ECS or both	Multivariate	Unilateral: 26 (74.3%), Bilateral: 9 (25.7%)	B/L ND: 9 (25.7%)	154	16, (45.7%)	7, (20%)
Urban et al. (2013) [49]	1338 (43%), 857 (28%), 896 (29%)	N/A	Univariate	NR	NR	2 (1-68)	287, (9.3%)	NR
Weckx et al. (2019) [50]	55 (35%), 29 (18%), 38 (24%), 13 (8%), 8 (5%), 16 (10%)	N/A	Univariate	Ipsilateral SND: 67 (42%), Ipsilateral MRND: 30 (19%), Ipsilateral RND: 6 (4%), Bilateral SND: 19 (12%), Ipsilateral MRND + Contralateral SND: 24 (15%), Bilateral MRND: 11 (7%), Ipsilateral RND + Contralateral SND: 2 (1%)	Ipsilateral MRND: 30 (19%), Ipsilateral RND: 6 (4%), Ipsilateral MRND + Contralateral SND: 24 (15%), Bilateral MRND: 11 (7%), Ipsilateral RND + Contralateral SND: 2	NR	21, (13%)	0, (0%)

					(1%)			
Xu et al. (2017) [51]	842 (41.3%), 366 (18%), 331 (16.3%), 217 (10.7%), 213 (10.4%), 67 (3.3%)	PNI, ECS, pathologic grade, gender, clinical features, T-stage	Multivariate	Bilateral: 305, Ipsilateral selective: 889/1568	B/L ND: 305	1.22 (0-55)	110, (26.3%)	0%
Yamagata et al. (2019) [52]	44 (46.3%), 28 (29.5%), 8 (8.4%), 8 (8.5%), 2 (2.2%), 5 (5.3%)	nodal disease area	Multivariate	RND: 47 (49.5%), SOHND: 37 (38.9%), RND + SOHND: 9 (9.5%), B/L SOHND: 2 (2.1%)	RND: 47 (49.5%), RND + SOHND: 9 (9.5%)	1 (0-33)	16, (16.8%)	Involved: 2, (2.2%) Close: 20, (20.1%)
Zhao et al. (2020) [53]	110 (44.4%), 39 (15.7%), 43 (17.3%), 16 (6.5%), 30 (12.1%), 10 (4%)	N/A	Univariate	NR	NR	1.9 (0-35)	37, (14.9%)	Involved: 0, (0%)

Table S6. Meta-regression analysis examining the role of potential modifiers in the association between lymph node ratio (LNR) and survival outcomes (continued).

Variables	Category or increment	OS - Studies analyzing exclusively patients with positive lymph nodes			DFS - Studies analyzing exclusively patients with positive lymph nodes			DSS - Studies analyzing exclusively patients with positive lymph nodes			OS - Studies analyzing patients with positive and negative lymph nodes		
		n§	Exponentiated coefficient (95% CI)	P	n§	Exponentiated coefficient (95% CI)	p	n§	Exponentiated coefficient (95% CI)	p	n§	Exponentiated coefficient (95% CI)	p
Percentage of males	10% increase	20	0.91 (0.78-1.07)	0.258	10	0.89 (0.72-1.09)	0.223	11	1.28 (0.87-1.87)	0.184	18	1.01 (0.85-1.21)	0.858
Mean age of study	10 year increase	18	1.05 (0.71-1.57)	0.788	8	Less than ten studies		11	0.70 (0.35-1.42)	0.282	16	0.79 (0.54-1.15)	0.204
Percentage of lip	10% increase	5	Less than ten studies		3	Less than ten studies		3	Less than ten studies		4	Less than ten studies	
Percentage of upper gum	10% increase		Insufficient data			Insufficient data			Insufficient data			Insufficient data	
Percentage of lower gum	10% increase		Insufficient data			Insufficient data			Insufficient data			Insufficient data	
Percentage of gum	10% increase		Insufficient data			Insufficient data			Insufficient data			Insufficient data	
Percentage of buccal mucosa	10% increase	13	1.04 (0.92-1.17)	0.496	5	Less than ten studies		8	Less than ten studies		13	0.98 (0.84-1.15)	0.784
Percentage of tongue	10% increase	17	**1.11 (1.04-1.19)**	**0.004**	8	Less than ten studies		8	Less than ten studies		18	**1.08 (1.01-1.16)**	**0.032**
Percentage of alveolus	10% increase		Insufficient data			Insufficient data			Insufficient data		3	Less than ten studies	
Percentage of retromolar trigone	10% increase	7	Less than ten studies		4	Less than ten studies		5	Less than ten studies		7	Less than ten studies	
Percentage of gingiva	10% increase	6	Less than ten studies			Insufficient data		5	Less than ten studies		3	Less than ten studies	

Percentage of hard palate	10% increase	8	Less than ten studies		3	Less than ten studies		5	Less than ten studies		8	Less than ten studies	
Percentage of floor of mouth	10% increase	13	**0.89(0.79-0.99)**	**0.033**	5	Less than ten studies		7	Less than ten studies		13	0.85 (0.72-1.00)	0.056
Percentage of radical dissection	10% increase	7	Less than ten studies		3	Less than ten studies		5	Less than ten studies		10	0.97 (0.88-1.07)	0.504
Median number of nodes removed	One node increase	17	0.99(0.96-1.01)	0.303	8	Less than ten studies		10	0.98 (0.92-1.03)	0.354	13	1.00 (0.95-1.05)	0.911
Median number of positive nodes removed	One positive node increase	16	1.09(0.92-1.28)	0.299	5	Less than ten studies		9	Less than ten studies		11	0.93 (0.65-1.34)	0.683
Percentage of extracapsular spread	10% increase	18	1.03(0.96-1.11)	0.383	10	1.05 (0.97-1.14)	0.214	8	Less than ten studies		10	1.11 (0.87-1.42)	0.341
Percentage of positive margins	10% increase	14	0.91(0.76-1.1)	0.306	8	Less than ten studies		8	Less than ten studies		15	0.82 (0.67-1.01)	0.060
Percentage of administered chemotherapy	10% increase	19	0.95(0.89-1.01)	0.071	10	0.93 (0.87-1.00)	0.053	9	Less than ten studies		13	0.93 (0.85-1.02)	0.126
Percentage of administered radiotherapy	10% increase	20	0.94(0.88-1.02)	0.128	10	1.00 (0.89-1.12)	0.987	11	0.97 (0.85-1.11)	0.595	14	0.93 (0.83-1.04)	0.187
Publication year	1 year increase	20	1.00(0.95-1.05)	0.955	10	0.94 (0.86-1.03)	0.165	11	1.01 (0.92-1.11)	0.797	18	1.00 (0.94-1.07)	0.998

§number of studies

Table S7. Evaluation of within-study risk of bias with the Newcastle-Ottawa Scale (continued).

Study	Selection				Comparability		Outcome			Total
	Representativeness	Selection of non-exposed	Ascertainment of exposure	Outcome not present at start	Comparability on pN-classification	Comparability on other factors	Assessment of outcome	Long enough follow-up (median ≥2 years)	Adequacy (completeness) of follow-up (≥90% response rate)	
Agarwal et al. (2019)	1	1	1	1	0	1	1	1	1	8
Arun et al. (2020)	1	1	1	1	0	0	1	0	1	6
Bharath et al. (2018)	1	1	1	1	0	0	1	1	0	6
Chang et al. (2018)	1	1	1	1	0	0	1	1	1	7
Chow et al. (2017)	1	1	1	1	0	0	1	1	1	7
Ding et al. (2019)	1	1	1	1	0	0	1	0	1	6
Ebrahimi et al. (2011)	1	1	1	1	0	1	1	1	1	8
Gil et al. (2009)	1	1	1	1	1	1	1	1	1	9
Hosni et al. (2017)	1	1	1	1	0	0	1	1	1	7
Iftikhar et al. (2020)	1	1	1	1	0	1	0	0	1	6
Jin et al. (2020)	1	1	1	1	0	0	1	1	1	7
Kim SY et al. (2011)	1	1	1	1	0	1	1	1	1	8

Künzel et al. (2014)	1	1	1	1	1	1	1	1	1	9
Lee C.C. et al. (2015)	1	1	1	1	0	1	1	1	1	8
Lee C.C. et al. (2017)	1	1	1	1	0	0	1	0	1	6
Lee H. et al. (2019)	1	1	1	1	1	1	1	1	1	9
Lieng et al. (2016)	1	1	1	1	0	0	1	1	1	7
Moratin et al. (2020)	1	1	1	1	0	1	1	0	1	7
Ong et al. (2016)	1	1	1	1	0	0	1	1	1	7
Patel et al. (2013)	1	1	1	1	1	1	1	1	1	9
Rempel et al. (2018)	1	1	1	1	1	1	1	1	1	9
Safi et al. (2017)	1	1	1	1	0	1	1	1	1	8
Shrime et al. (2009)	1	1	1	1	0	0	1	1	1	7
Son et al. (2017)	1	1	1	1	0	1	1	1	1	8
Spoerl et al. (2020)	1	1	1	1	0	0	1	1	1	7
Subramaniam et al.(2019)	1	1	0	1	0	0	0	1	0	4
Suzuki et al. (2016)	1	1	1	1	0	1	1	0	1	7
Urban et al. (2013)	1	1	1	1	0	0	1	0	1	6
Weckx et al. (2019)	1	1	1	1	0	0	1	1	1	7
Xu et al. (2017)	1	1	1	1	0	1	1	1	0	7
Yamagata et al. (2019)	1	1	1	1	0	1	0	0	0	5
Zhao et al. (2020)	1	1	1	1	1	1	1	1	1	9

Figure S1. Forest plot describing the association between lymph node ratio (LNR) and recurrence-free survival (RFS) in group YES. Apart from the overall analysis, the subanalyses by LNR cut-off values are presented.

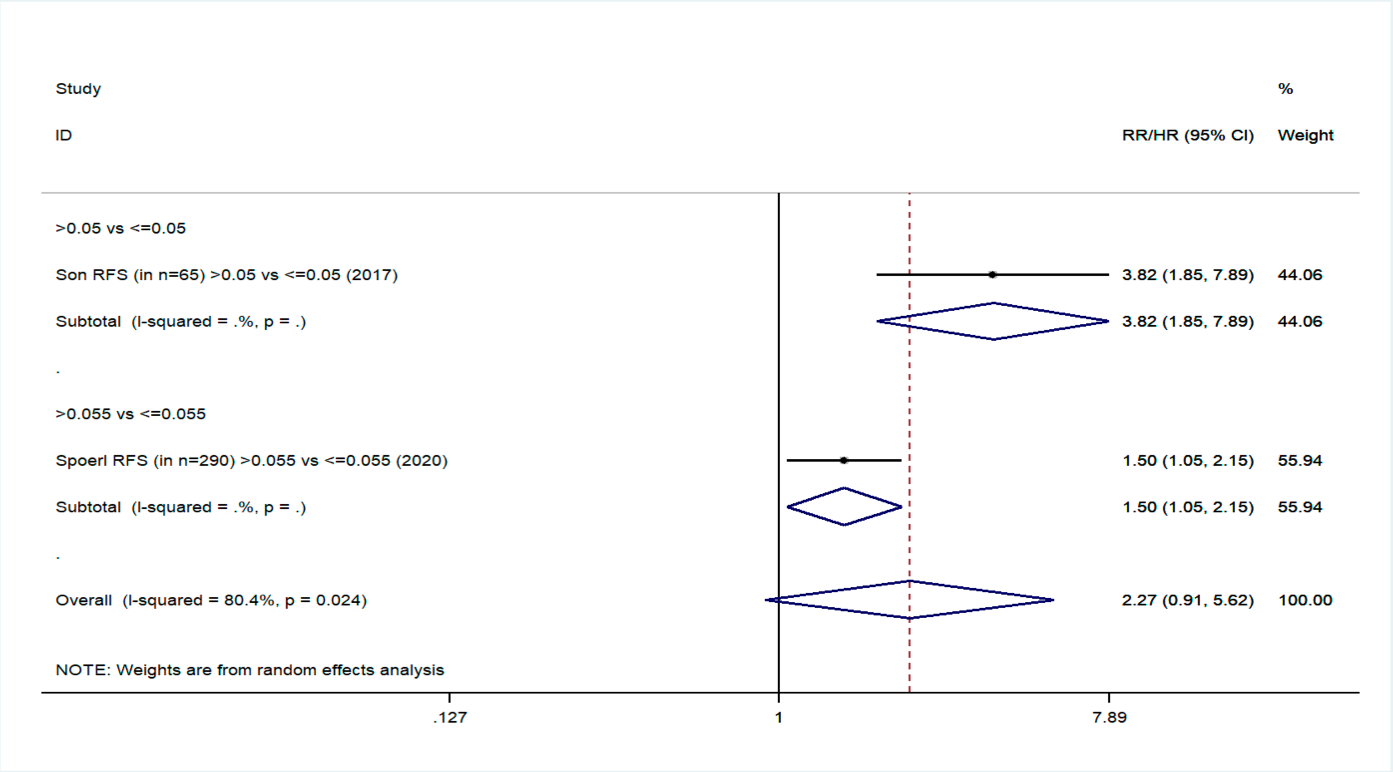

Figure S2. Forest plot describing the association between lymph node ratio (LNR) and locoregional disease-free survival (LRDFS) in group NO. Apart from the overall analysis, the subanalyses by LNR cut-off values are presented.

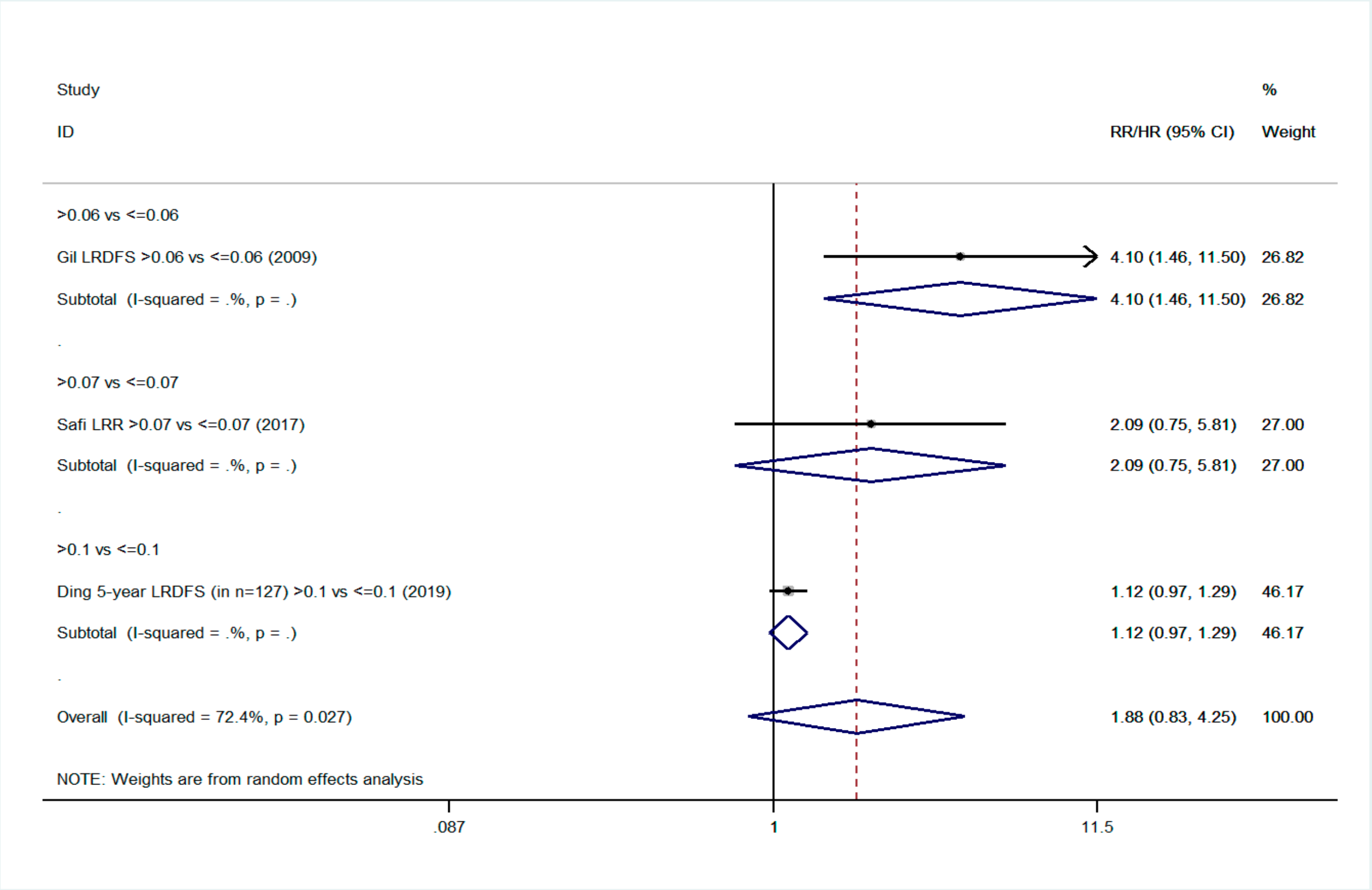

Figure S3. Forest plot describing the association between lymph node ratio (LNR) and distant metastasis-free survival (DMFS) in group NO. Apart from the overall analysis, the subanalyses by LNR cut-off values are presented.

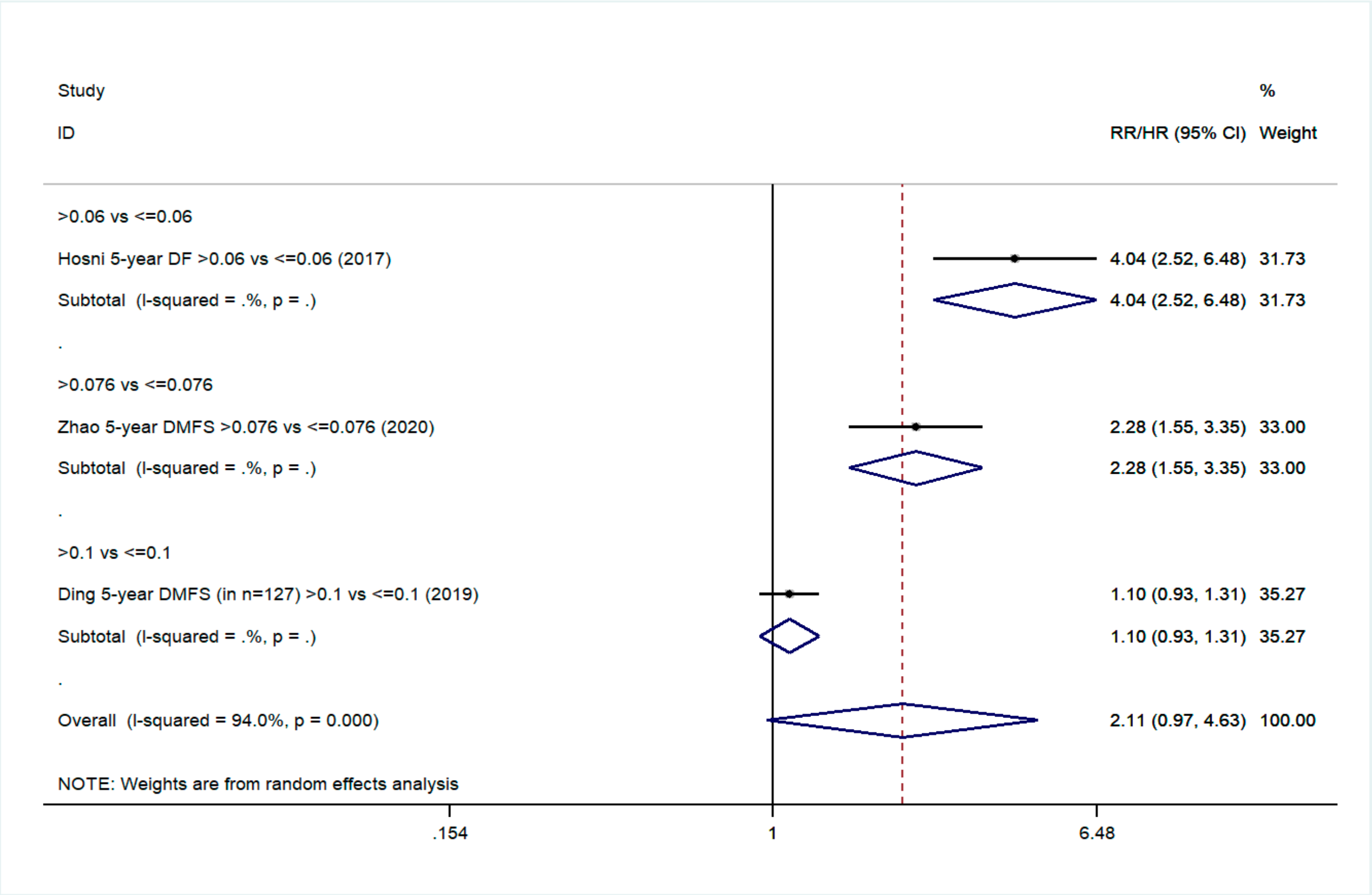

Figure S4. Plot depicting the modifying effect mediated by percentage of tumors affecting the tongue upon the association between high lymph node ratio (LNR) values and overall survival (OS) in group YES. The circle sizes represent the inverse of each within-study variance.

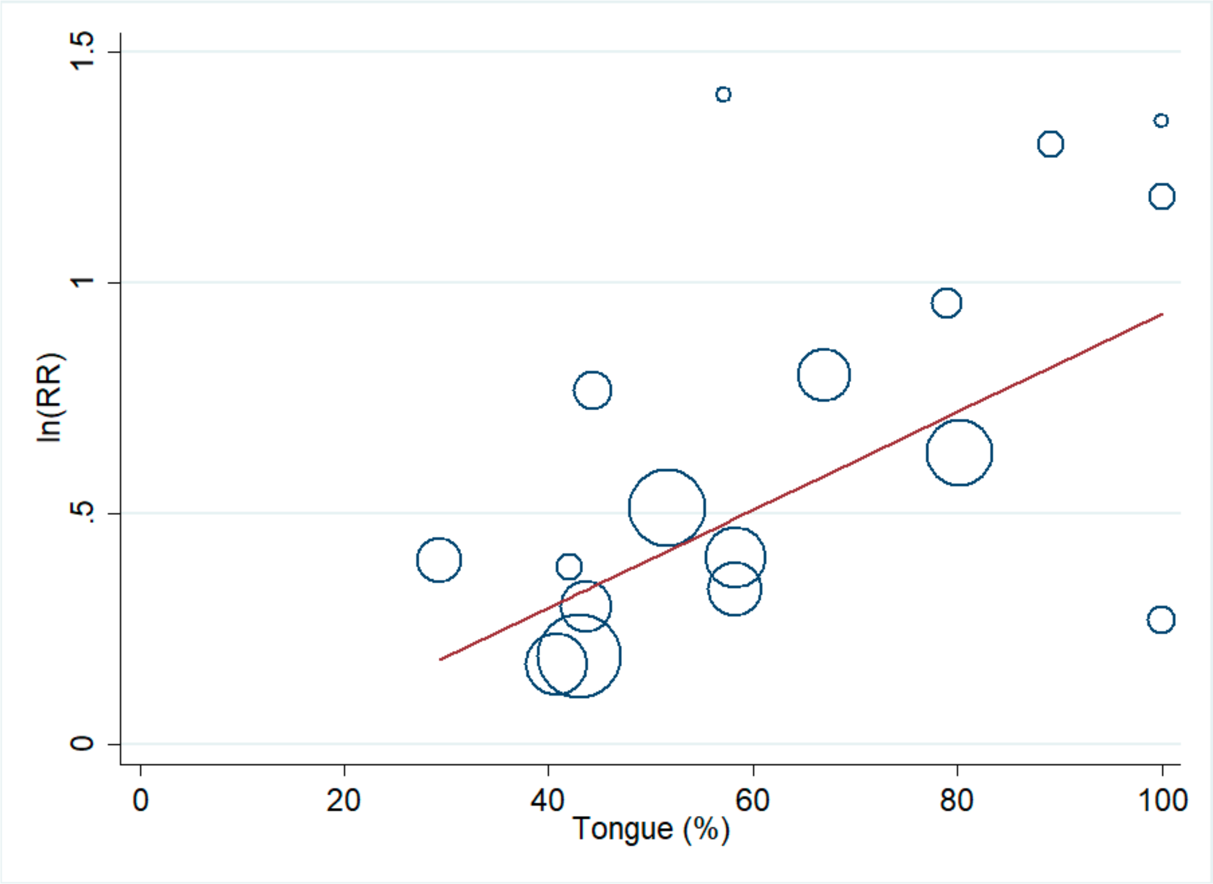

Figure S5. Plot depicting the modifying effect mediated by percentage of tumors affecting the floor of mouth upon the association between high lymph node ratio (LNR) values and overall survival in group YES. The circle sizes represent the inverse of each within-study variance.

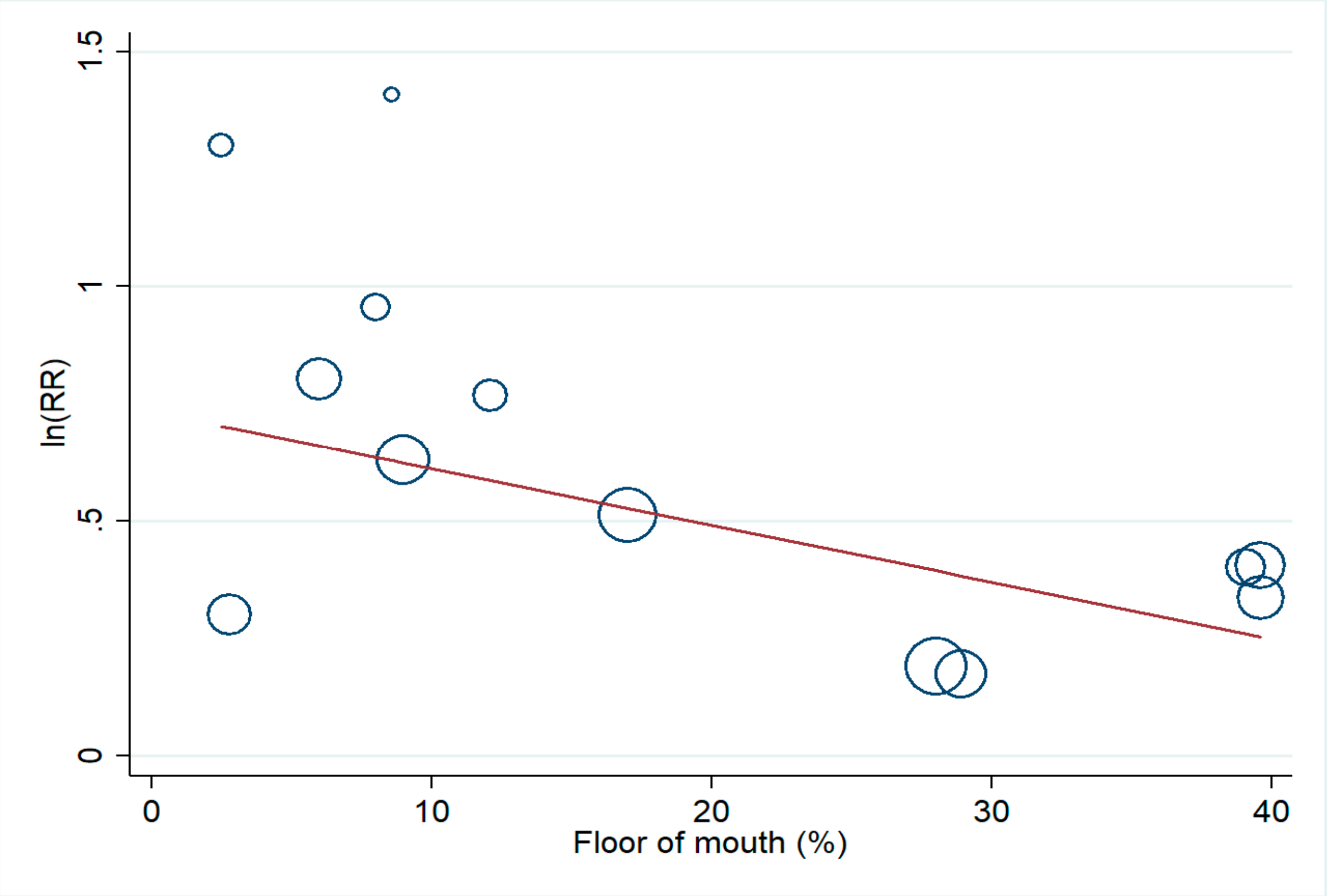

Figure S6. Plot depicting the modifying effect mediated by percentage of tumors affecting the tongue upon the association between high lymph node ratio (LNR) values and overall survival in group NO. The circle sizes represent the inverse of each within-study variance.

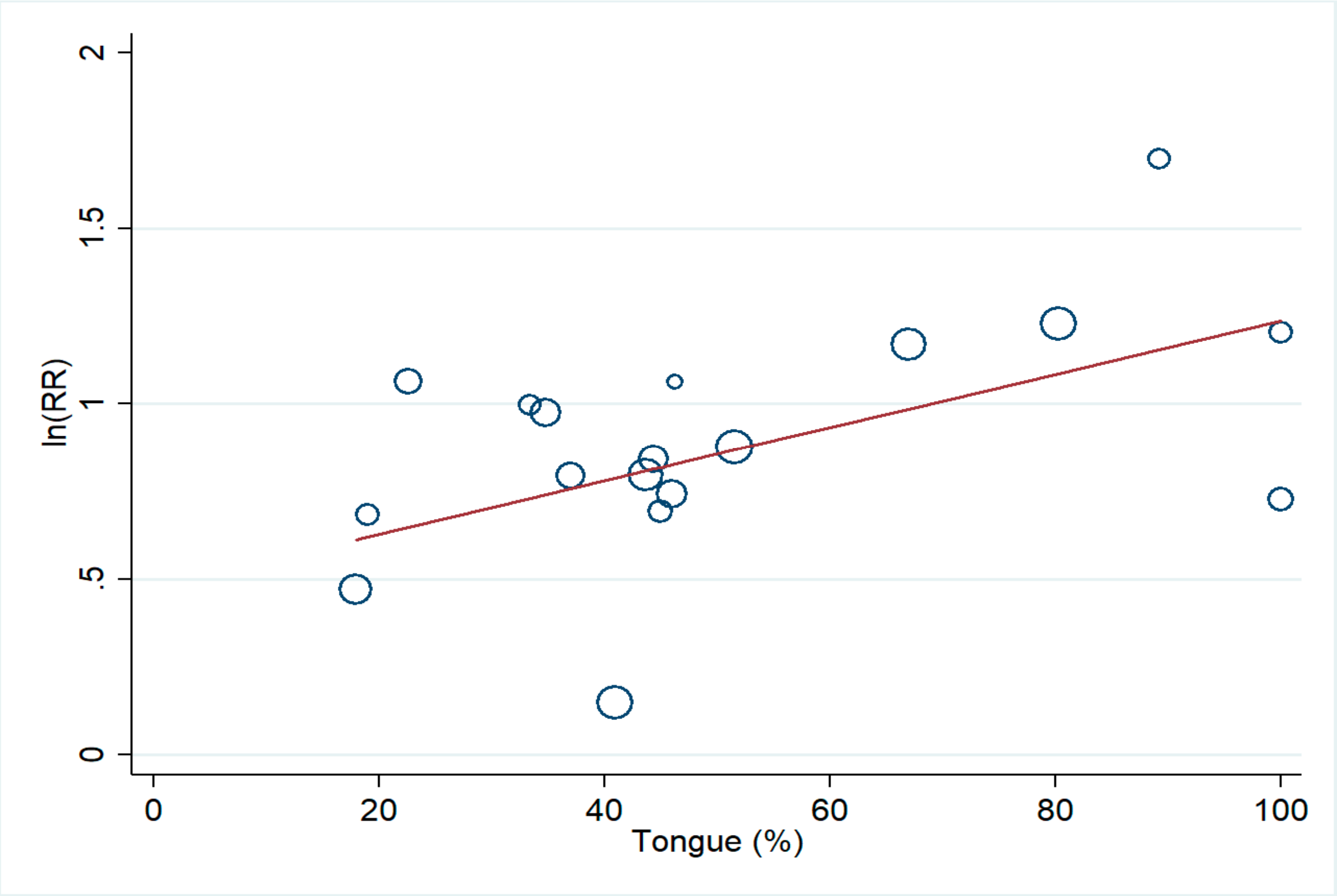

Figure S7. Funnel plot of the meta-analysis on overall survival in group YES showing evidence of publication bias as considerable asymmetry.

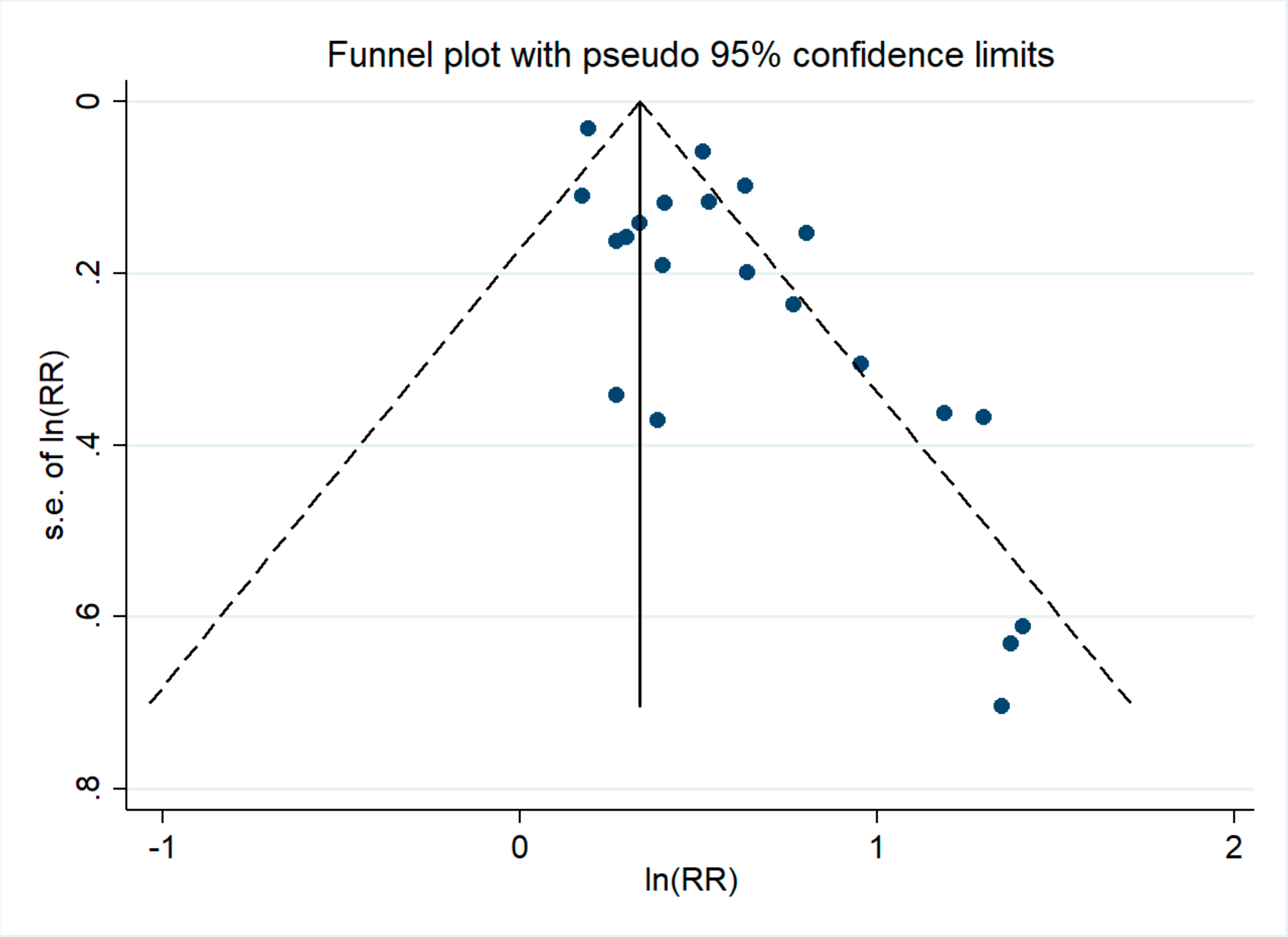
Funnel plot with pseudo 95% confidence limits
s.e. of ln(RR)
0
.2
.4
.6
.8
-1
0
1
2
ln(RR)

Figure S8. Funnel plot of the meta-analysis on disease-specific survival in group YES showing evidence of publication bias as considerable asymmetry.

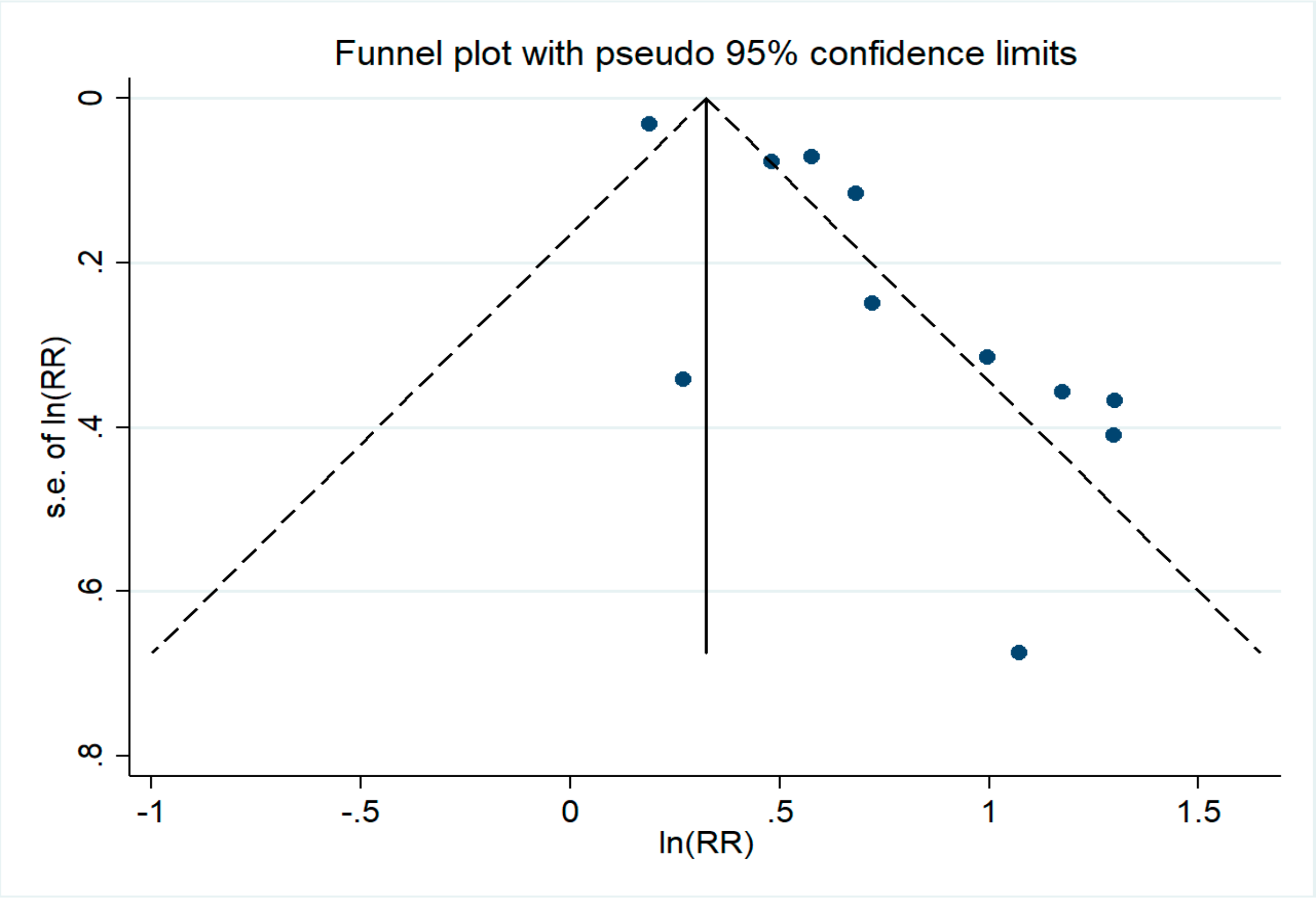

Figure S9. Funnel plot of the meta-analysis on disease-free survival in group YES without obvious asymmetry, i.e. no evidence of publication bias.

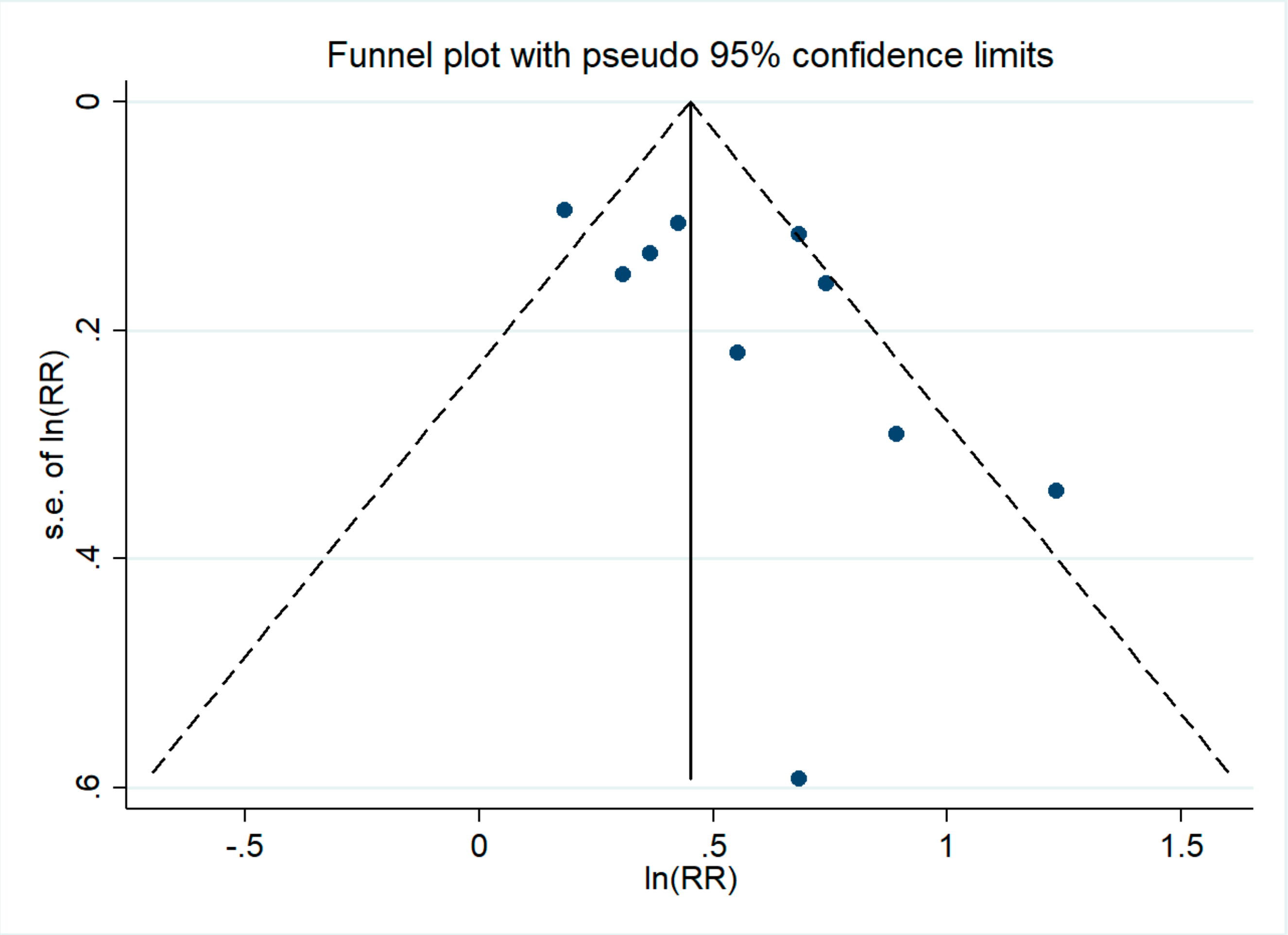

Figure S10. Funnel plot of the meta-analysis on overall survival in group NO without obvious asymmetry, i.e. no evidence of publication bias.

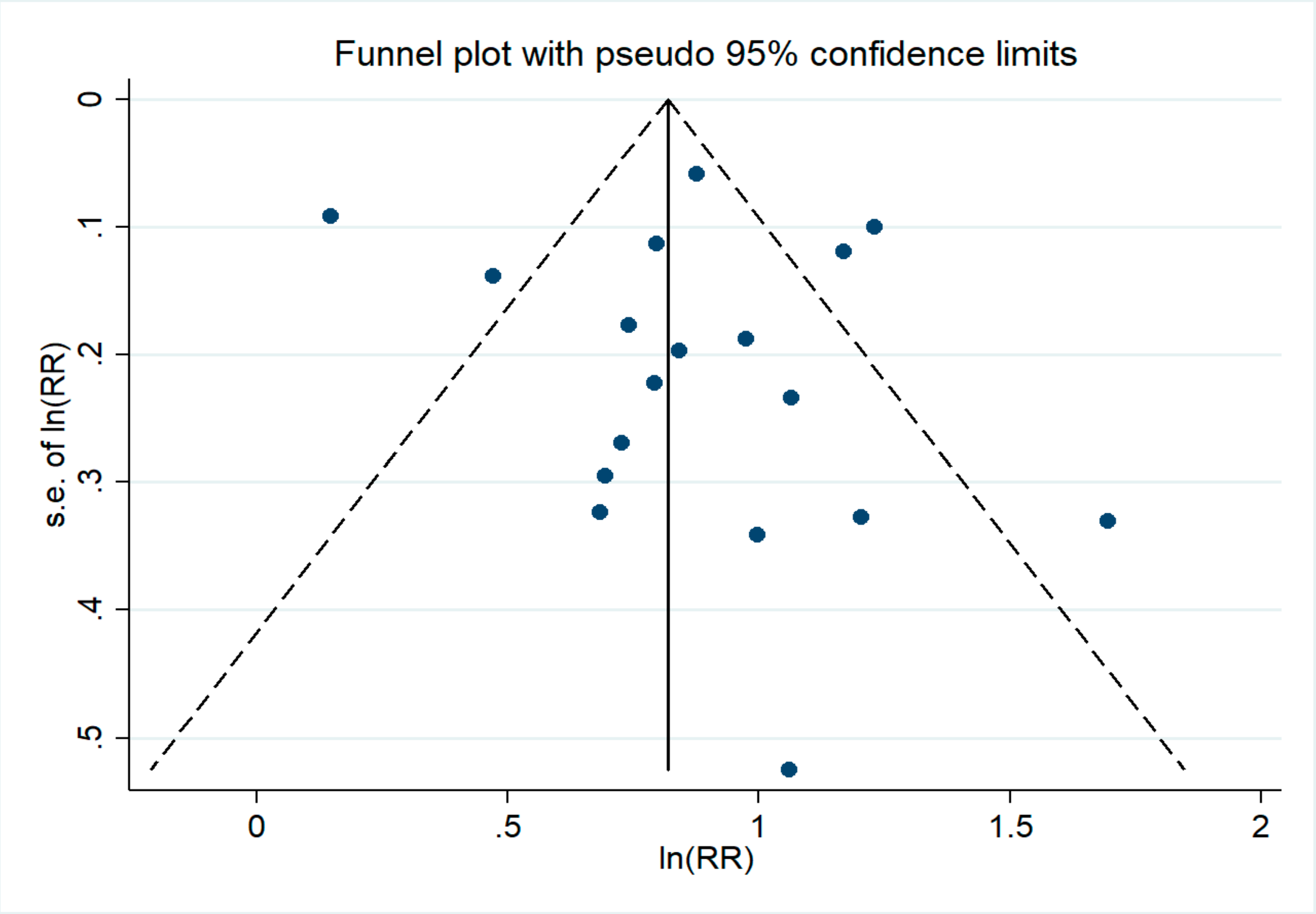

Figure S11. Forest plot describing the association between lymph node ratio (LNR) and overall survival (OS) in group YES. Apart from the overall analysis, the subanalyses on degree of adjustment are presented.

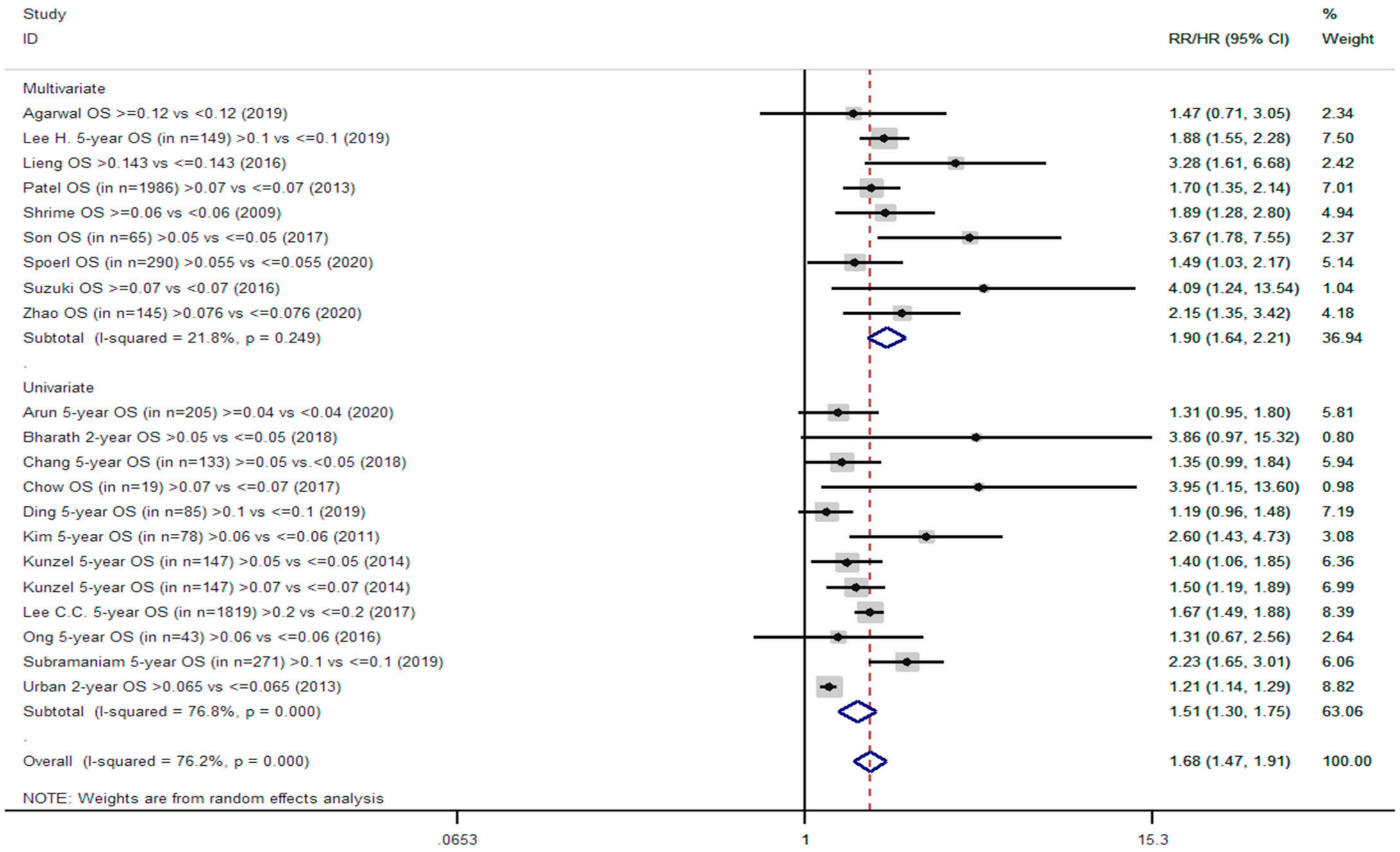

Figure S12. Forest plot describing the association between lymph node ratio (LNR) and disease-free survival (DFS) in group YES. Apart from the overall analysis, the subanalyses on degree of adjustment are presented.

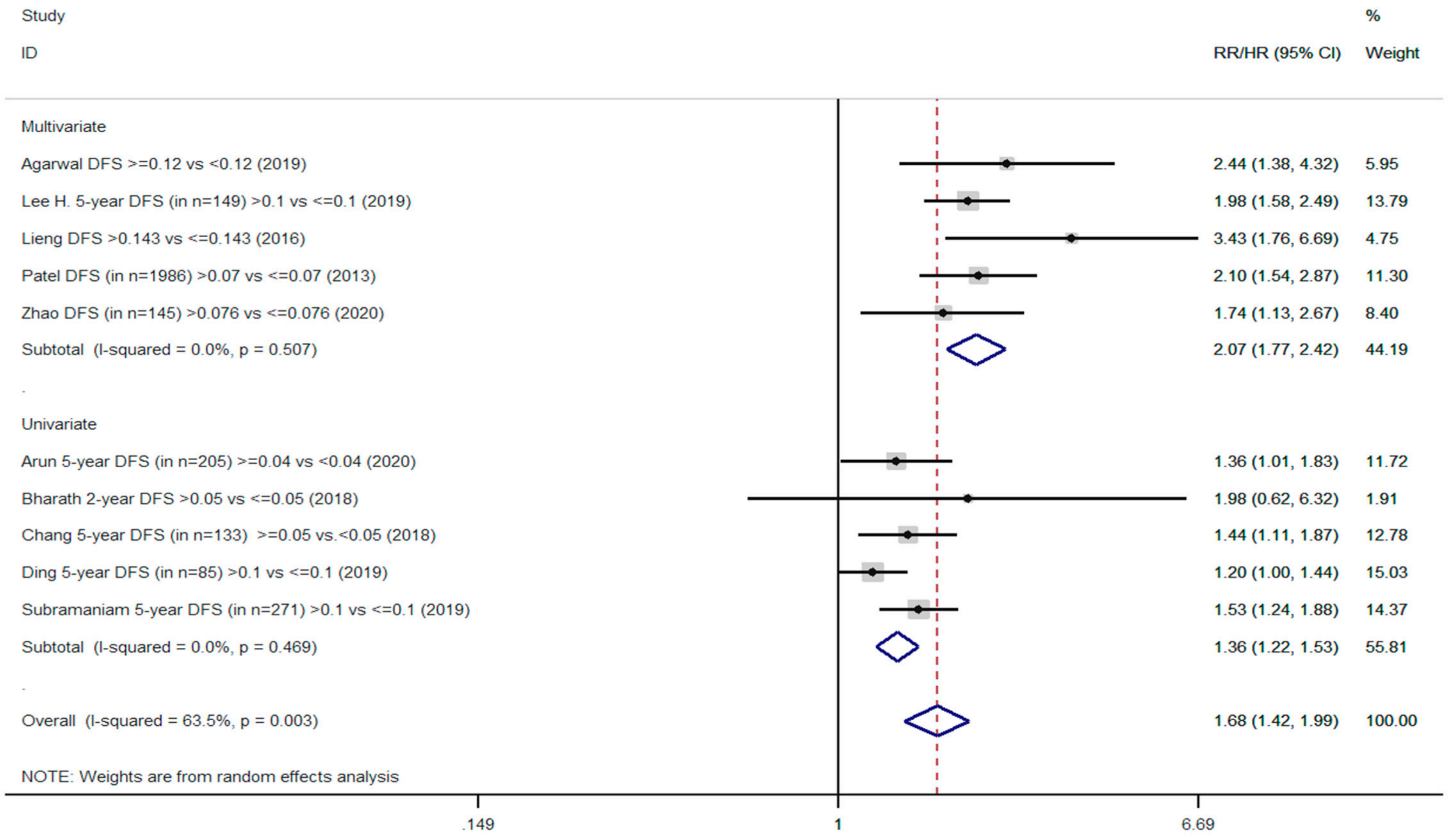

Figure S13. Forest plot describing the association between lymph node ratio (LNR) and disease-specific survival (DSS) in group YES. Apart from the overall analysis, the subanalyses on degree of adjustment are presented.

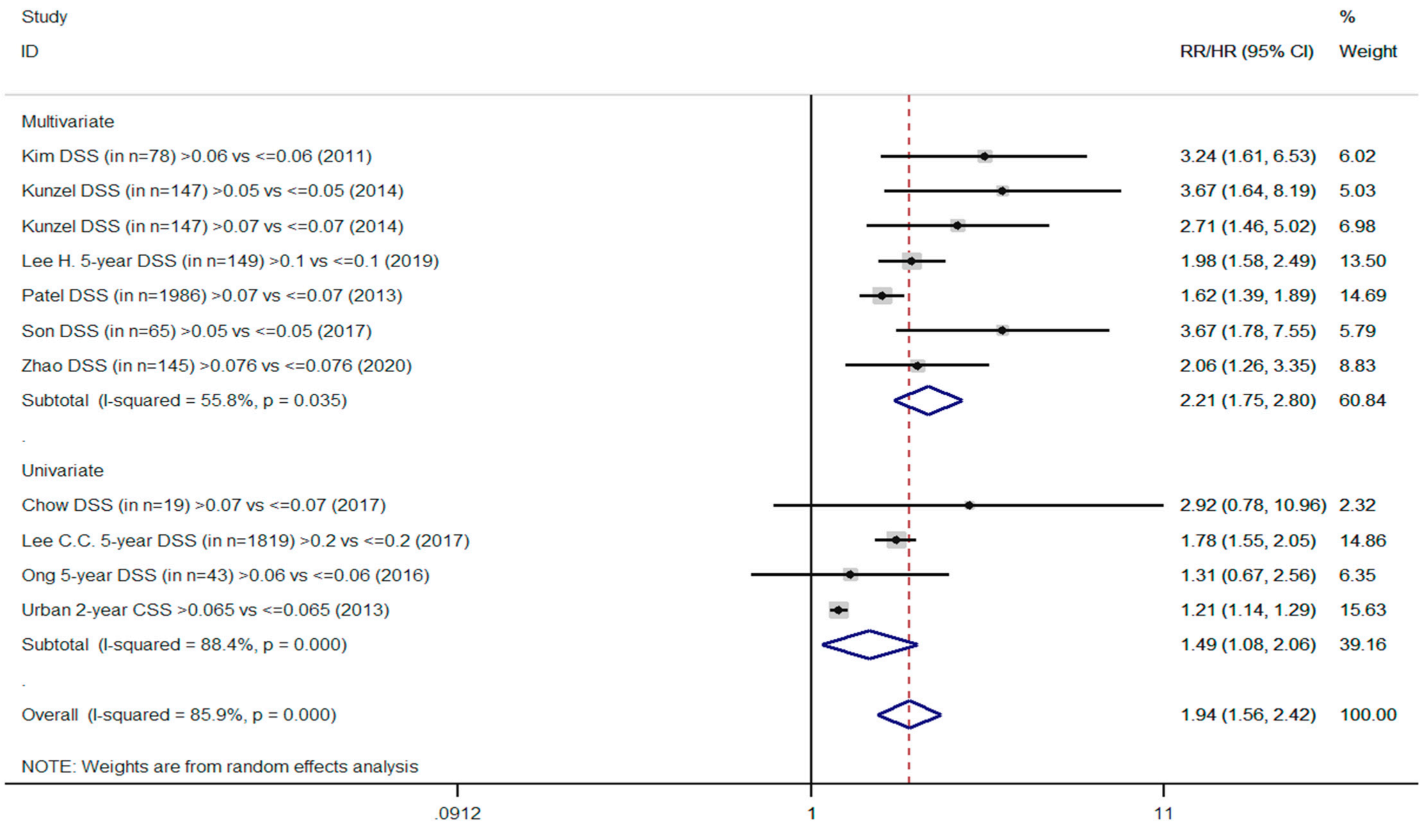

Figure S14. Forest plot describing the association between lymph node ratio (LNR) and distant metastasis-free survival (DMFS) in group YES. Apart from the overall analysis, the subanalyses on degree of adjustment are presented.

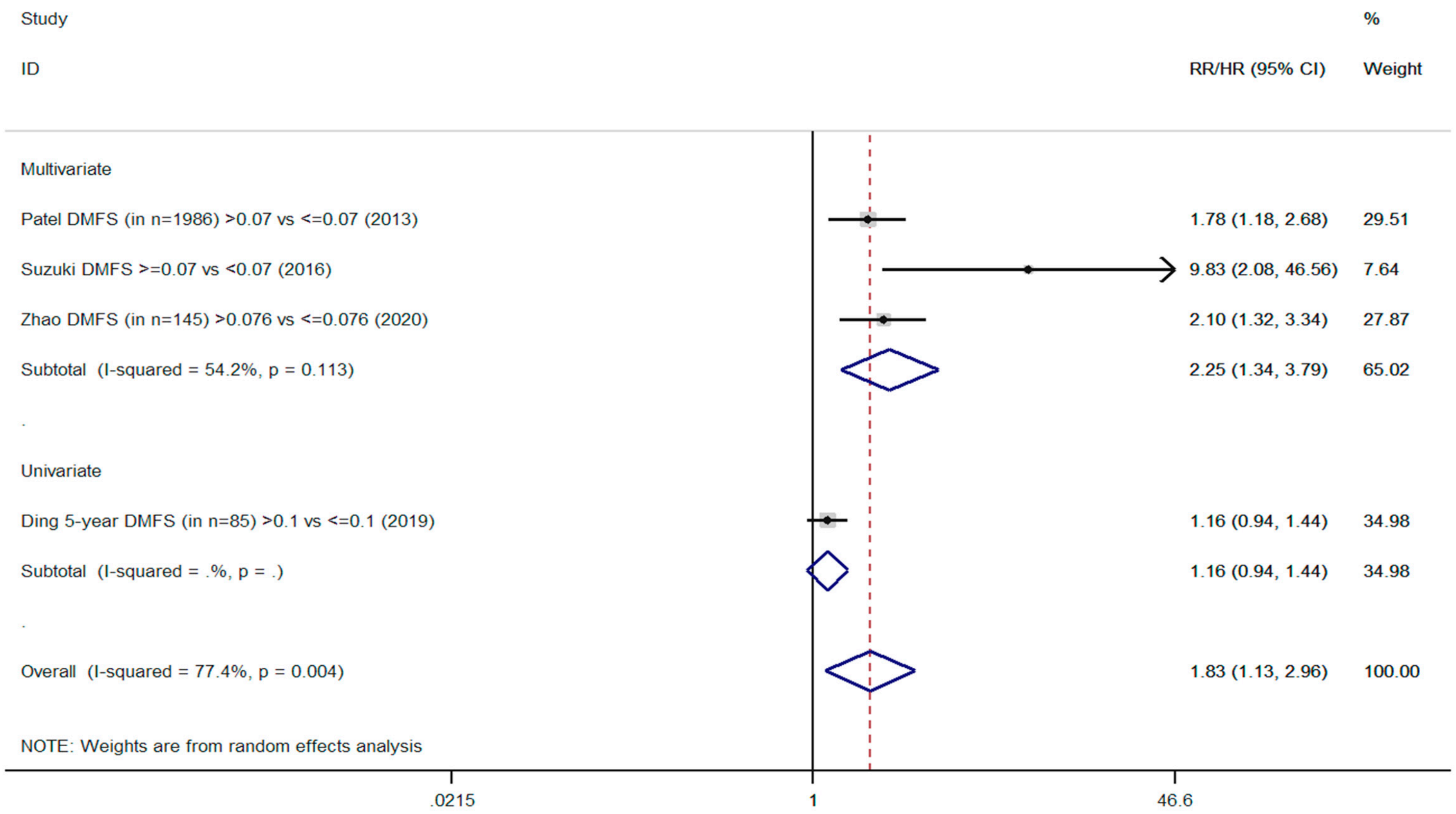

Figure S15. Forest plot describing the association between lymph node ratio (LNR) and locoregional disease-free survival (LRDFS) in group YES. Apart from the overall analysis, the subanalyses on degree of adjustment are presented.

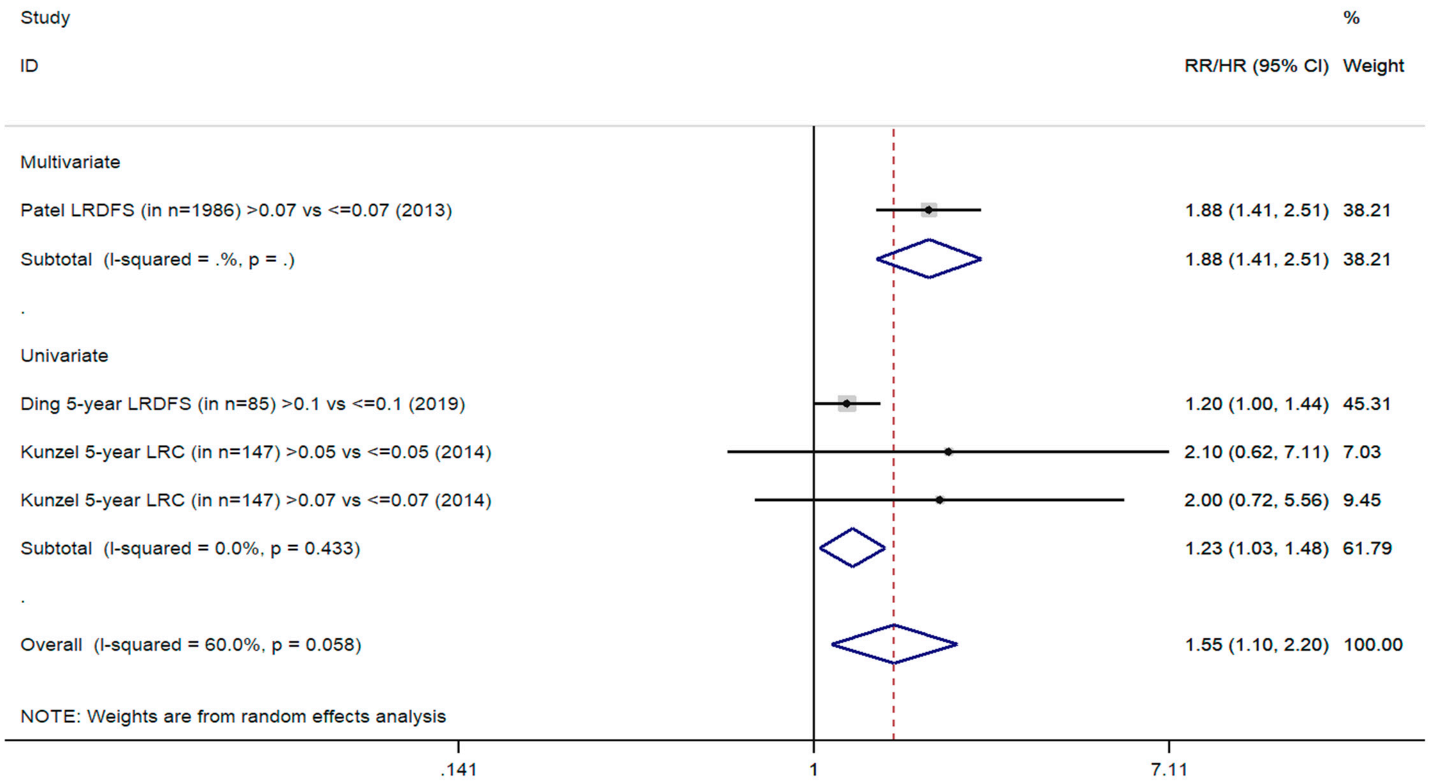

Figure S16. Forest plot describing the association between lymph node ratio (LNR) and local recurrence-free survival (LRFS) in group YES. Apart from the overall analysis, the subanalyses on degree of adjustment are presented.

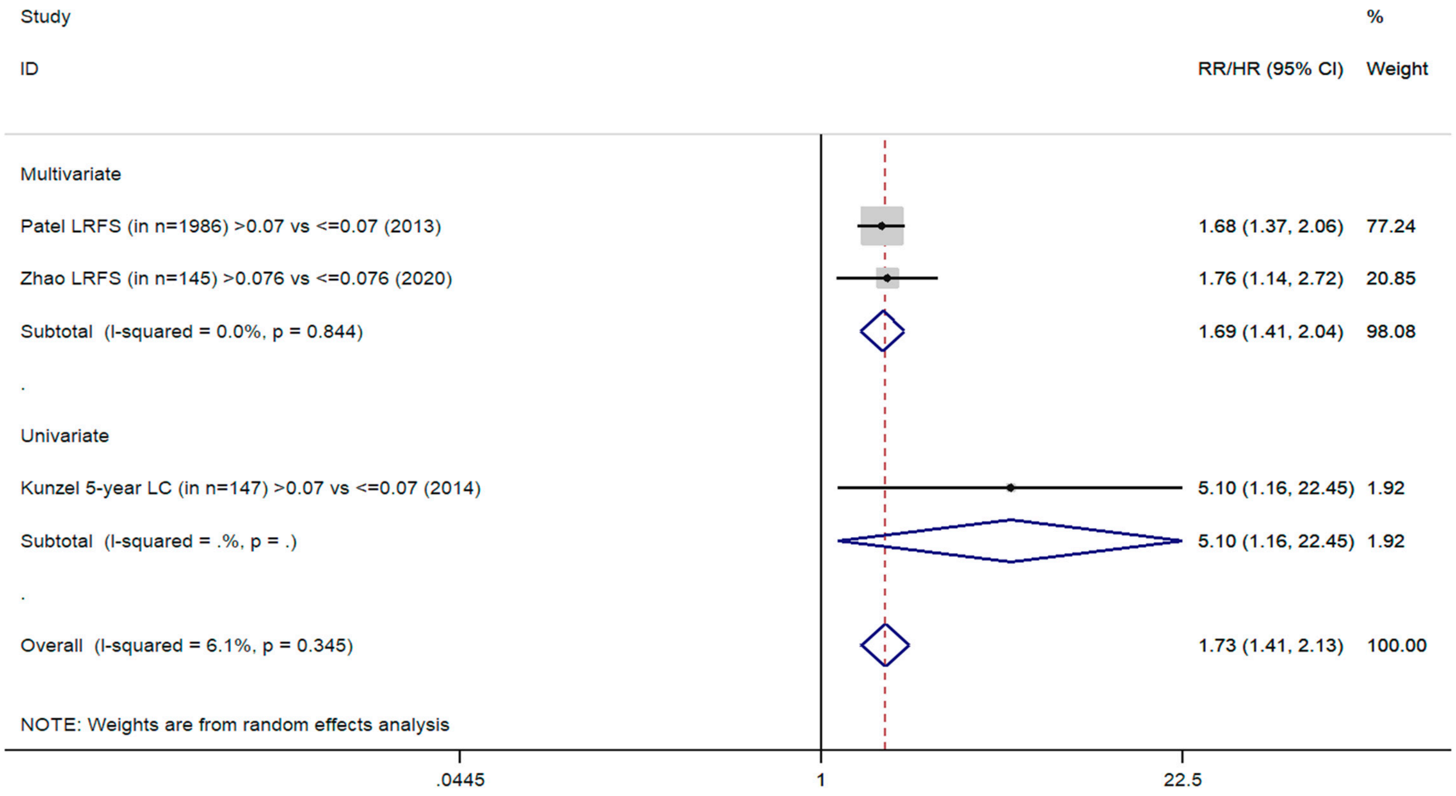

Figure S17. Forest plot describing the association between lymph node ratio (LNR) and recurrence-free survival (RFS) in group YES. Apart from the overall analysis, the subanalyses on degree of adjustment are presented.

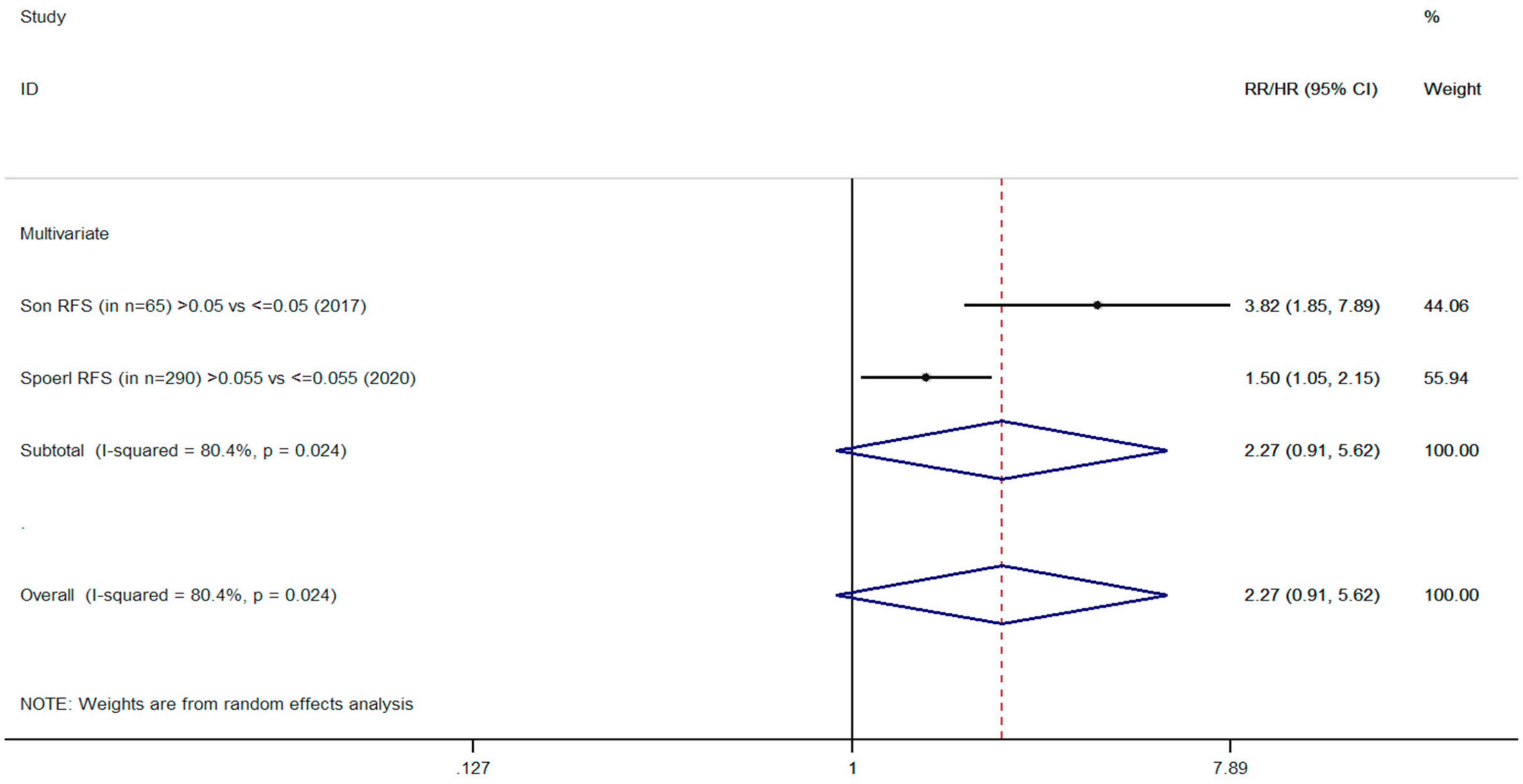

Figure S18. Forest plot describing the association between lymph node ratio (LNR) and overall survival (OS) in group NO. Apart from the overall analysis, the subanalyses on degree of adjustment are presented.

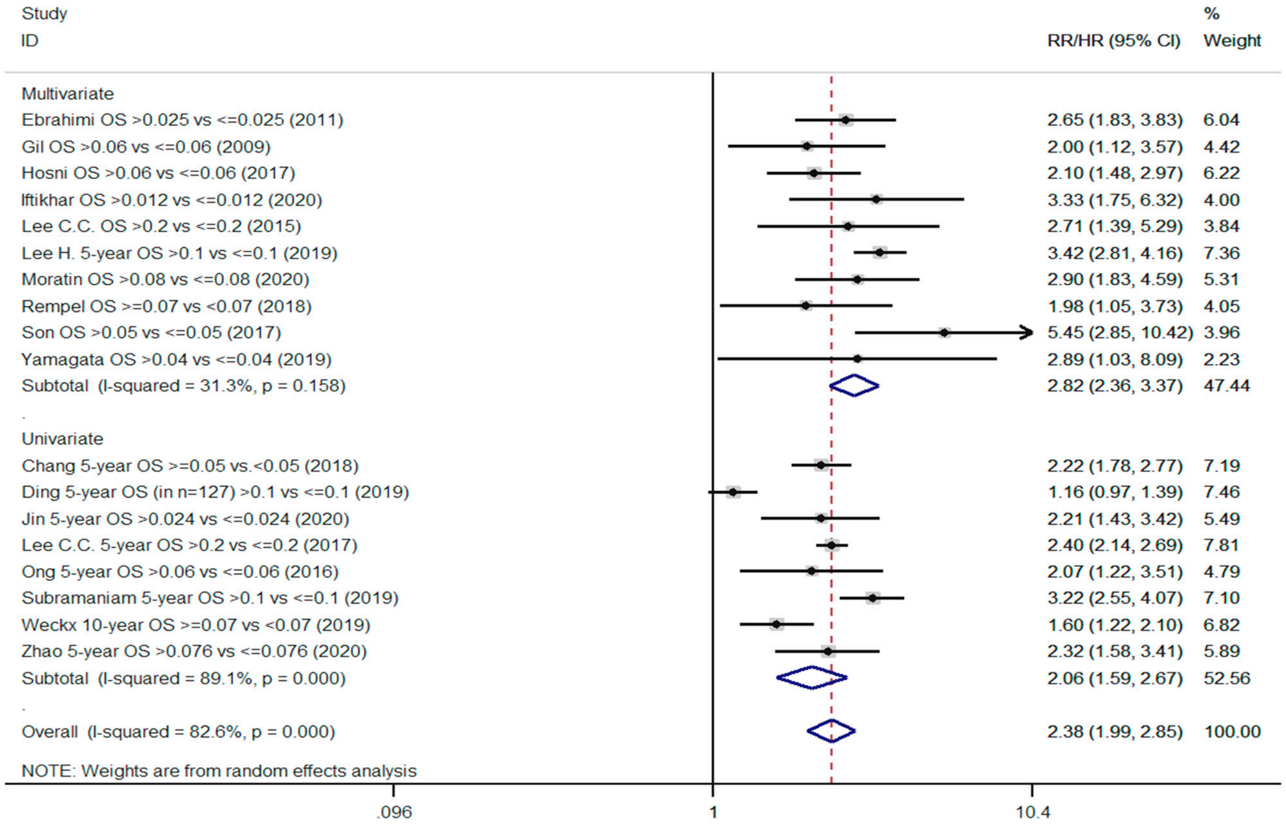

Figure S19. Forest plot describing the association between lymph node ratio (LNR) and disease-free survival (DFS) in group NO. Apart from the overall analysis, the subanalyses on degree of adjustment are presented.

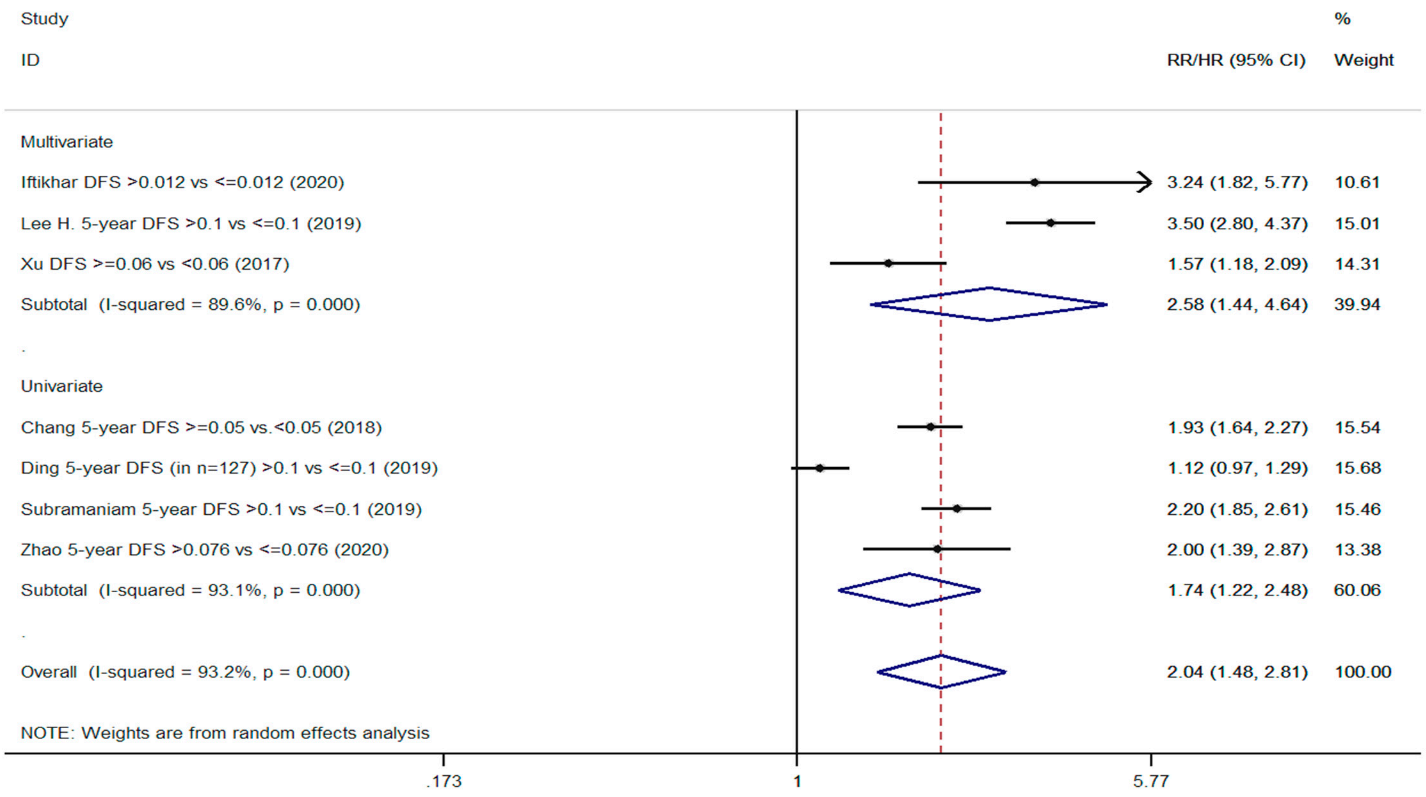

Figure S20. Forest plot describing the association between lymph node ratio (LNR) and disease-specific survival (DSS) in group NO. Apart from the overall analysis, the subanalyses on degree of adjustment are presented.

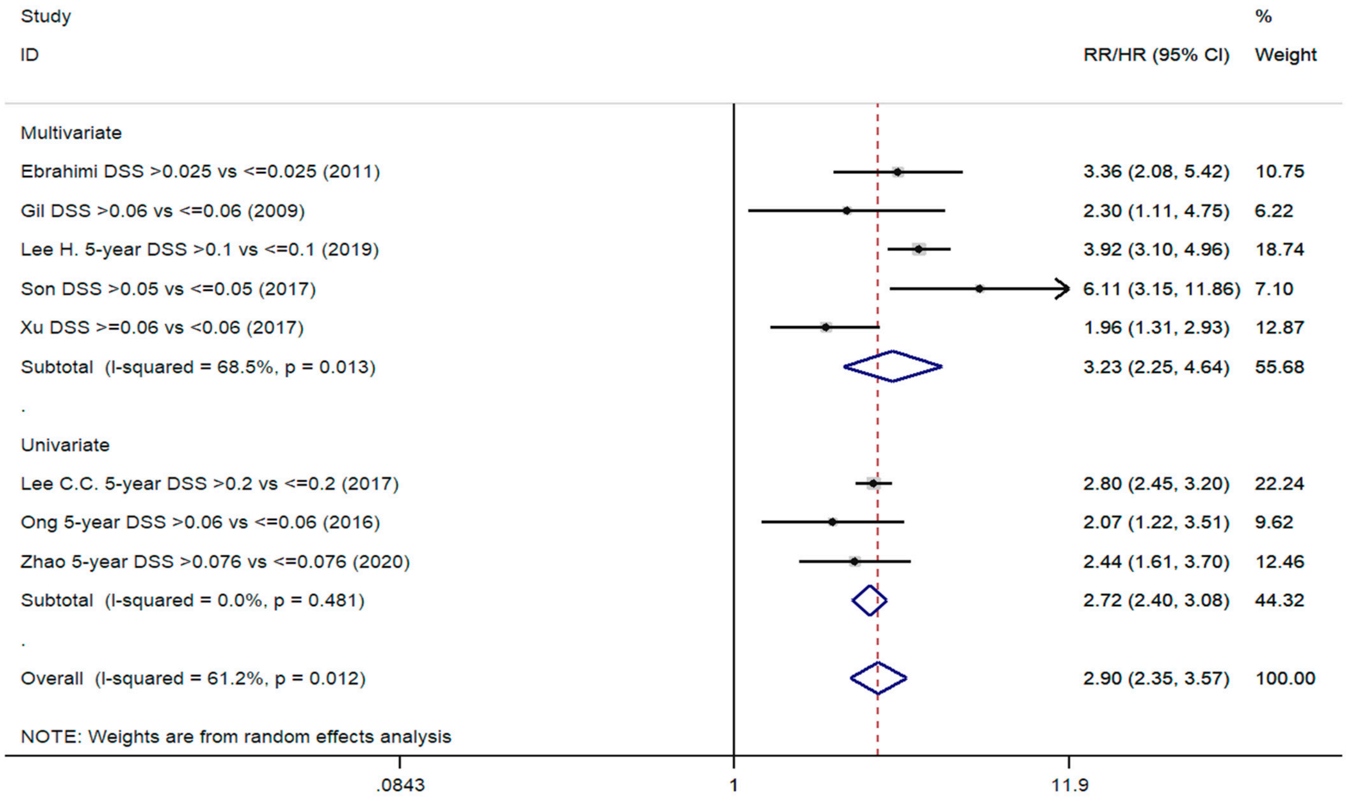

Figure S21. Forest plot describing the association between lymph node ratio (LNR) and locoregional disease-free survival (LRDFS) in group NO. Apart from the overall analysis, the subanalyses on degree of adjustment are presented.

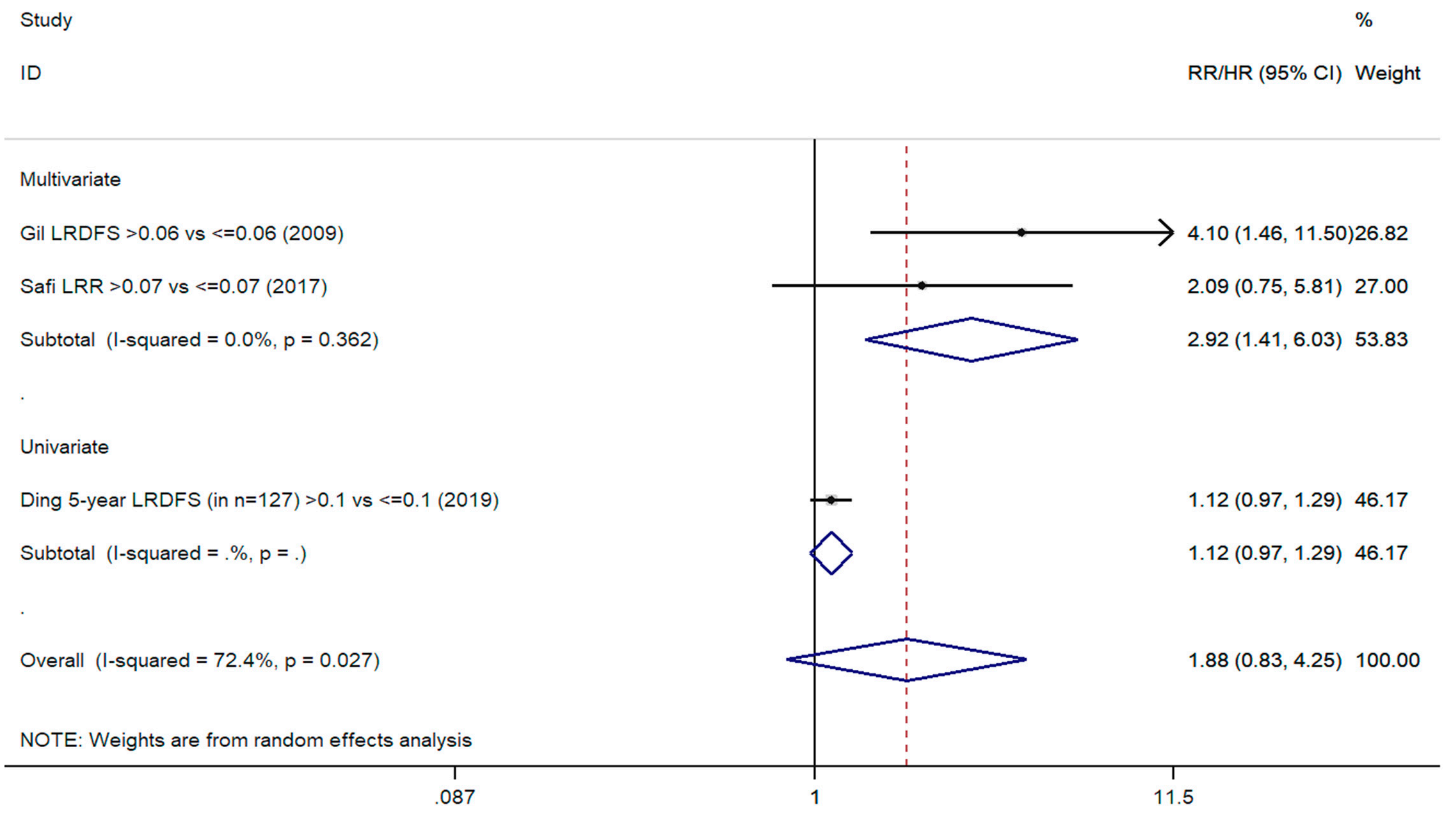

Figure S22. Forest plot describing the association between lymph node ratio (LNR) and distant metastasis-free survival (DMFS) in group NO. Apart from the overall analysis, the subanalyses on degree of adjustment are presented.

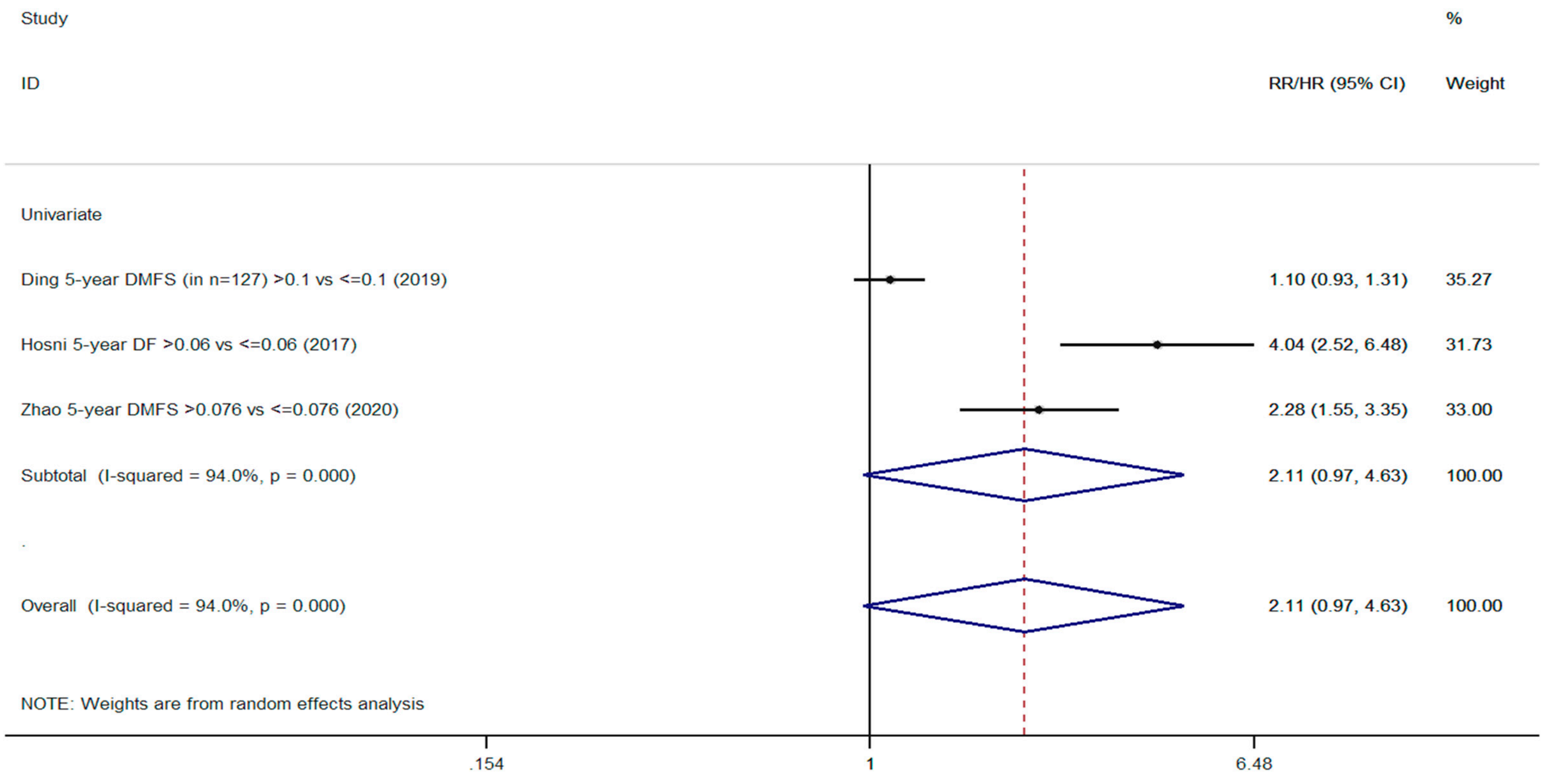

References

1. Liberati, A.; Altman, D.G.; Tetzlaff, J.; Mulrow, C.; Gøtzsche, P.C.; Ioannidis, J.P.A.; Clarke, M.; Devereaux, P.J.; Kleijnen, J.; Moher, D. The PRISMA Statement for Reporting Systematic Reviews and Meta-Analyses of Studies That Evaluate Health Care Interventions: Explanation and Elaboration. *Journal of Clinical Epidemiology* **2009**, *62*, e1–e34, doi:10.1016/j.jclinepi.2009.06.006.
2. Adel, M.; Tsao, C.-K.; Wei, F.-C.; Chien, H.-T.; Lai, C.-H.; Liao, C.-T.; Wang, H.-M.; Fan, K.-H.; Kang, C.-J.; Chang, J.T.-C.; et al. Preoperative SCC Antigen, CRP Serum Levels, and Lymph Node Density in Oral Squamous Cell Carcinoma. *Medicine* **2016**, *95*, e3149, doi:10.1097/MD.0000000000003149.
3. Amar, A.; Rapoport, A.; Curioni, O.A.; Dedivitis, R.A.; Cernea, C.R.; Brandão, L.G. A Densidade Do Linfonodo Metastático Como Fator Prognóstico No Carcinoma Espinocelular Da Língua e Soalho Bucal. *Braz. j. otorhinolaryngol.* **2012**, *78*, 86–90, doi:10.1590/S1808-86942012000300015.
4. Chen, C.-C.; Lin, J.-C.; Chen, K.-W. Lymph Node Ratio as a Prognostic Factor in Head and Neck Cancer Patients. *Radiat Oncol* **2015**, *10*, 181, doi:10.1186/s13014-015-0490-9.
5. Faisal, M.; Dhanani, R.; Ullah, S.; Bakar, M.A.; Irfan, N.; Malik, K.I.; Loya, A.; Boban, E.M.; Hussain, R.; Jamshed, A. Prognostic Outcomes of Treatment Naïve Oral Tongue Squamous Cell Carcinoma (OTSCC): A Comprehensive Analysis of 14 Years. *Eur Arch Otorhinolaryngol* **2021**, *278*, 3045–3053, doi:10.1007/s00405-020-06482-x.
6. Feng, Z.; Xu, Q.S.; Wang, C.; Li, J.Z.; Mao, M.H.; Li, H.; Qin, L.Z.; Han, Z. Lymph Node Ratio Is Associated with Adverse Clinicopathological Features and Is a Crucial Nodal Parameter for Oral and Oropharyngeal Cancer. *Sci Rep* **2017**, *7*, 6708, doi:10.1038/s41598-017-07134-7.
7. Hingsammer, L.; Seier, T.; Ikenberg, J.; Schumann, P.; Zweifel, D.; Rücker, M.; Bredell, M.; Lanzer, M. The Influence of Lymph Node Ratio on Survival and Disease Recurrence in Squamous Cell Carcinoma of the Tongue. *International Journal of Oral and Maxillofacial Surgery* **2019**, *48*, 851–856, doi:10.1016/j.ijom.2019.01.008.
8. Iocca, O.; Di Maio, P.; De Virgilio, A.; Pellini, R.; Golusiński, P.; Petruzzi, G.; Zocchi, J.; Pirola, F.; Janczak, R.; Golusiński, W.; et al. Lymph Node Yield and Lymph Node Ratio in Oral Cavity and Oropharyngeal Carcinoma: Preliminary Results from a Prospective, Multicenter, International Cohort. *Oral Oncology* **2020**, *107*, 104740, doi:10.1016/j.oraloncology.2020.104740.
9. Kim, K.-Y.; Cha, I.-H. Risk Stratification of Oral Cancer Patients Using a Combined Prognostic Factor Including Lymph Node Density and Biomarker. *J Cancer Res Clin Oncol* **2012**, *138*, 483–490, doi:10.1007/s00432-011-1129-3.
10. Kim, K.-Y.; Zhang, X.; Kim, S.-M.; Lee, B.-D.; Cha, I.-H. A Combined Prognostic Factor for Improved Risk Stratification of Patients with Oral Cancer. *Oral Dis* **2017**, *23*, 91–96, doi:10.1111/odi.12579.
11. Liao, C.-T.; Hsueh, C.; Lee, L.-Y.; Lin, C.-Y.; Fan, K.-H.; Wang, H.-M.; Huang, S.-F.; Chen, I.-H.; Kang, C.-J.; Ng, S.-H.; et al. Neck Dissection Field and Lymph Node Density Predict Prognosis in Patients with Oral Cavity Cancer and Pathological Node Metastases Treated with Adjuvant Therapy. *Oral Oncology* **2012**, *48*, 329–336, doi:10.1016/j.oraloncology.2011.10.017.

12. Mascitti, M.; Rubini, C.; De Michele, F.; Balercia, P.; Girotto, R.; Troiano, G.; Lo Muzio, L.; Santarelli, A. American Joint Committee on Cancer Staging System 7th Edition versus 8th Edition: Any Improvement for Patients with Squamous Cell Carcinoma of the Tongue? *Oral Surgery, Oral Medicine, Oral Pathology and Oral Radiology* **2018**, *126*, 415–423, doi:10.1016/j.oooo.2018.07.052.
13. Noble, A.R.; Greskovich, J.F.; Han, J.; Reddy, C.A.; Nwizu, T.I.; Khan, M.F.; Scharpf, J.; Adelstein, D.J.; Burkey, B.B.; Koyfman, S.A. Risk Factors Associated with Disease Recurrence in Patients with Stage III/IV Squamous Cell Carcinoma of the Oral Cavity Treated with Surgery and Postoperative Radiotherapy. *Anticancer Res* **2016**, *36*, 785–792.
14. Roberts, T.J.; Colevas, A.D.; Hara, W.; Holsinger, F.C.; Oakley-Girvan, I.; Divi, V. Number of Positive Nodes Is Superior to the Lymph Node Ratio and American Joint Committee on Cancer N Staging for the Prognosis of Surgically Treated Head and Neck Squamous Cell Carcinomas: Lymph Node Prognostics for HNSCC. *Cancer* **2016**, *122*, 1388–1397, doi:10.1002/cncr.29932.
15. Safi, A.-F.; Grandoch, A.; Nickenig, H.-J.; Zöller, J.E.; Kreppel, M. Importance of Lymph Node Ratio for Locoregional Recurrence of Squamous Cell Carcinoma of the Buccal Mucosa. *Head & Neck* **2017**, *39*, 2488–2493, doi:10.1002/hed.24922.
16. Safi, A.-F.; Grandoch, A.; Nickenig, H.-J.; Zöller, J.E.; Kreppel, M. The Importance of Lymph Node Ratio for Locoregional Recurrence of Squamous Cell Carcinoma of the Tongue. *Journal of Cranio-Maxillofacial Surgery* **2017**, *45*, 1058–1061, doi:10.1016/j.jcms.2017.04.008.
17. Safi, A.-F.; Kauke, M.; Grandoch, A.; Nickenig, H.-J.; Zöller, J.; Kreppel, M. The Importance of Lymph Node Ratio for Patients with Mandibular Infiltration of Oral Squamous Cell Carcinoma. *J Craniomaxillofac Surg* **2018**, *46*, 1007–1012, doi:10.1016/j.jcms.2018.03.021.
18. Sayed, S.I.; Sharma, S.; Rane, P.; Vaishampayan, S.; Talole, S.; Chaturvedi, P.; Chaukar, D.; Deshmukh, A.; Agarwal, J.P.; D'cruz, A.K. Can Metastatic Lymph Node Ratio (LNR) Predict Survival in Oral Cavity Cancer Patients?: Can Metastatic LNR Predict Survival. *J. Surg. Oncol.* **2013**, *108*, 256–263, doi:10.1002/jso.23387.
19. Shrime, M.G.; Ma, C.; Gullane, P.J.; Gilbert, R.W.; Irish, J.C.; Brown, D.H.; Goldstein, D.P. Impact of Nodal Ratio on Survival in Squamous Cell Carcinoma of the Oral Cavity. *Head Neck* **2009**, *31*, 1129–1136, doi:10.1002/hed.21073.
20. Troeltzsch, M.; Haidari, S.; Boser, S.; Troeltzsch, M.; Probst, F.A.; Ehrenfeld, M.; Otto, S. What Factors Are Associated With Regional Recurrence After Operative Treatment of Oral Squamous Cell Carcinoma? *Journal of Oral and Maxillofacial Surgery* **2018**, *76*, 2650–2659, doi:10.1016/j.joms.2018.07.005.
21. Zirk, M.; Safi, A.-F.; Buller, J.; Nickenig, H.-J.; Dreiseidler, T.; Zinser, M.; Drebber, U.; Zöller, J.E.; Kreppel, M. Lymph Node Ratio as Prognosticator in Floor of Mouth Squamous Cell Carcinoma Patients. *J Craniomaxillofac Surg* **2018**, *46*, 195–200, doi:10.1016/j.jcms.2017.11.021.
22. Agarwal, J.P.; Kane, S.; Ghosh-Laskar, S.; Pilar, A.; Manik, V.; Oza, N.; Wagle, P.; Gupta, T.; Budrukkar, A.; Murthy, V.; et al. Extranodal Extension in Resected Oral Cavity Squamous Cell Carcinoma: More to It than Meets the Eye. *Laryngoscope* **2019**, *129*, 1130–1136, doi:10.1002/lary.27508.
23. Arun, I.; Maity, N.; Hameed, S.; Jain, P.V.; Manikantan, K.; Sharan, R.; Arun, P. Lymph Node Characteristics and Their Prognostic Significance in Oral Squamous Cell Carcinoma. *Head Neck* **2021**, *43*, 520–533, doi:10.1002/hed.26499.

24. Bharath, V.M.; Balagopal, P.G.; Nebu, A.G.; Jayasudha, A.V.; Iqbal Ahmed, M.; Sebastian, P. Can Metastatic Lymph Node Ratio Be Used as an Independent Prognostic Factor in Carcinoma Tongue? *Gulf J Oncolog* **2018**, *1*, 6–10.
25. Chang, W.-C.; Lin, C.-S.; Yang, C.-Y.; Lin, C.-K.; Chen, Y.-W. Lymph Node Density as a Prognostic Predictor in Patients with Betel Nut-Related Oral Squamous Cell Carcinoma. *Clin Oral Investig* **2018**, *22*, 1513–1521, doi:10.1007/s00784-017-2247-3.
26. Chow, T.-L.; Kwan, W.W.Y.; Fung, S.-C.; Ho, L.-I. Prognostic Value of Lymph Node Density in Buccal Squamous Cell Carcinoma. *Am J Otolaryngol* **2017**, *38*, 529–532, doi:10.1016/j.amjoto.2017.05.001.
27. Ding, D.; Stokes, W.; Eguchi, M.; Hararah, M.; Sumner, W.; Amini, A.; Goddard, J.; Somerset, H.; Bradley, C.; McDermott, J.; et al. Association Between Lymph Node Ratio and Recurrence and Survival Outcomes in Patients With Oral Cavity Cancer. *JAMA Otolaryngol Head Neck Surg* **2019**, *145*, 53–61, doi:10.1001/jamaoto.2018.2974.
28. Ebrahimi, A.; Clark, J.R.; Zhang, W.J.; Elliott, M.S.; Gao, K.; Milross, C.G.; Shannon, K.F. Lymph Node Ratio as an Independent Prognostic Factor in Oral Squamous Cell Carcinoma. *Head Neck* **2011**, *33*, 1245–1251, doi:10.1002/hed.21600.
29. Gil, Z.; Carlson, D.L.; Boyle, J.O.; Kraus, D.H.; Shah, J.P.; Shaha, A.R.; Singh, B.; Wong, R.J.; Patel, S.G. Lymph Node Density Is a Significant Predictor of Outcome in Patients with Oral Cancer. *Cancer* **2009**, *115*, 5700–5710, doi:10.1002/cncr.24631.
30. Hosni, A.; McMullen, C.; Huang, S.H.; Xu, W.; Su, J.; Bayley, A.; Bratman, S.V.; Cho, J.; Giuliani, M.; Kim, J.; et al. Lymph Node Ratio Relationship to Regional Failure and Distant Metastases in Oral Cavity Cancer. *Radiother Oncol* **2017**, *124*, 225–231, doi:10.1016/j.radonc.2017.06.018.
31. Iftikhar, H.; Rozi, S.; Zahid, N.; Awan, M.S.; Nathani, K.R. Lymph Node Ratio as a Prognostic Marker of Oral Tongue Squamous Cell Carcinoma: A Cohort Study. *Ann R Coll Surg Engl* **2020**, *102*, 726–732, doi:10.1308/rcsann.2020.0173.
32. Jin, W.; Zhu, Z.; Wu, Y.; Ding, X.; Wu, H.; Song, X.; Wu, Y. Prognostic Value of Log Odds of Positive Lymph Nodes in Patients with Resectable Oral Squamous Cell Carcinoma. *Oral Oncol* **2020**, *108*, 104709, doi:10.1016/j.oraloncology.2020.104709.
33. Kim, S.Y.; Nam, S.Y.; Choi, S.-H.; Cho, K.-J.; Roh, J.-L. Prognostic Value of Lymph Node Density in Node-Positive Patients with Oral Squamous Cell Carcinoma. *Ann Surg Oncol* **2011**, *18*, 2310–2317, doi:10.1245/s10434-011-1614-6.
34. Künzel, J.; Mantsopoulos, K.; Psychogios, G.; Grundtner, P.; Koch, M.; Iro, H. Lymph Node Ratio as a Valuable Additional Predictor of Outcome in Selected Patients with Oral Cavity Cancer. *Oral Surg Oral Med Oral Pathol Oral Radiol* **2014**, *117*, 677–684, doi:10.1016/j.oooo.2014.02.032.
35. Lee, C.-C.; Ho, H.-C.; Su, Y.-C.; Lee, M.-S.; Hung, S.-K.; Chen, Y.-L. The Prognostic Ability of Log Odds of Positive Lymph Nodes in Oral Cavity Squamous Cell Carcinoma. *Medicine (Baltimore)* **2015**, *94*, e1069, doi:10.1097/MD.0000000000001069.
36. Lee, C.-C.; Su, Y.-C.; Hung, S.-K.; Chen, P.-C.; Huang, C.-I.; Huang, W.-L.; Lin, Y.-W.; Yang, C.-C. Recommendation for Incorporation of a Different Lymph Node Scoring System in Future AJCC N Category for Oral Cancer. *Sci Rep* **2017**, *7*, 14117, doi:10.1038/s41598-017-06452-0.
37. Lee, H.; Roh, J.-L.; Cho, K.-J.; Choi, S.-H.; Nam, S.Y.; Kim, S.Y. Number of Positive Lymph Nodes Better Predicts Survival for Oral Cavity Cancer. *J Surg Oncol* **2019**, *119*, 675–682, doi:10.1002/jso.25386.

38. Lieng, H.; Gebski, V.J.; Morgan, G.J.; Veness, M.J. Important Prognostic Significance of Lymph Node Density in Patients with Node Positive Oral Tongue Cancer. *ANZ J Surg* **2016**, *86*, 681–686, doi:10.1111/ans.13512.
39. Moratin, J.; Metzger, K.; Kansy, K.; Ristow, O.; Engel, M.; Hoffmann, J.; Flechtenmacher, C.; Freier, K.; Freudlsperger, C.; Horn, D. The Prognostic Significance of the Lymph Node Ratio in Oral Cancer Differs for Anatomical Subsites. *Int J Oral Maxillofac Surg* **2020**, *49*, 558–563, doi:10.1016/j.ijom.2019.10.015.
40. Ong, W.; Zhao, R.; Lui, B.; Tan, W.; Ebrahimi, A.; Clark, J.R.; Soo, K.-C.; Tan, N.-C.; Tan, H.-K.; Iyer, N.G. Prognostic Significance of Lymph Node Density in Squamous Cell Carcinoma of the Tongue. *Head Neck* **2016**, *38 Suppl 1*, E859-866, doi:10.1002/hed.24113.
41. Patel, S.G.; Amit, M.; Yen, T.C.; Liao, C.T.; Chaturvedi, P.; Agarwal, J.P.; Kowalski, L.P.; Ebrahimi, A.; Clark, J.R.; Cernea, C.R.; et al. Lymph Node Density in Oral Cavity Cancer: Results of the International Consortium for Outcomes Research. *Br J Cancer* **2013**, *109*, 2087–2095, doi:10.1038/bjc.2013.570.
42. Rempel, V.; Safi, A.F.; Drebber, U.; Nickenig, H.J.; Neugebauer, J.; Zöller, J.E.; Kreppel, M. The Prognostic Relevance of Lymph Node Ratio in Patients with Oral Squamous Cell Carcinoma Treated with Neoadjuvant Therapy Regimen and Radical Surgery. *J Craniomaxillofac Surg* **2018**, *46*, 1659–1663, doi:10.1016/j.jcms.2018.05.053.
43. Safi, A.-F.; Kauke, M.; Grandoch, A.; Nickenig, H.-J.; Drebber, U.; Zöller, J.; Kreppel, M. The Importance of Log Odds of Positive Lymph Nodes for Locoregional Recurrence in Oral Squamous Cell Carcinoma. *Oral Oncol* **2017**, *72*, 48–55, doi:10.1016/j.oraloncology.2017.07.005.
44. Shrime, M.G.; Bachar, G.; Lea, J.; Volling, C.; Ma, C.; Gullane, P.J.; Gilbert, R.W.; Irish, J.C.; Brown, D.H.; Goldstein, D.P. Nodal Ratio as an Independent Predictor of Survival in Squamous Cell Carcinoma of the Oral Cavity. *Head Neck* **2009**, *31*, 1482–1488, doi:10.1002/hed.21114.
45. Son, H.-J.; Roh, J.-L.; Cho, K.-J.; Choi, S.-H.; Nam, S.Y.; Kim, S.Y. Nodal Factors Predictive of Recurrence and Survival in Patients with Oral Cavity Squamous Cell Carcinoma. *Clin Otolaryngol* **2018**, *43*, 470–476, doi:10.1111/coa.12995.
46. Spoerl, S.; Gerken, M.; Mamilos, A.; Fischer, R.; Wolf, S.; Nieberle, F.; Klingelhöffer, C.; Meier, J.K.; Spoerl, S.; Ettl, T.; et al. Lymph Node Ratio as a Predictor for Outcome in Oral Squamous Cell Carcinoma: A Multicenter Population-Based Cohort Study. *Clin Oral Investig* **2021**, *25*, 1705–1713, doi:10.1007/s00784-020-03471-6.
47. Subramaniam, N.; Balasubramanian, D.; Kumar, N.; Murthy, S.; Vijayan, S.N.; Nambiar, A.; Vidhyadharan, S.; Thankappan, K.; Iyer, S. Lymph Node Staging Systems in Oral Squamous Cell Carcinoma: A Comparative Analysis. *Oral Oncol* **2019**, *97*, 92–98, doi:10.1016/j.oraloncology.2019.08.002.
48. Suzuki, H.; Beppu, S.; Hanai, N.; Hirakawa, H.; Hasegawa, Y. Lymph Node Density Predicts Lung Metastases in Oral Squamous Cell Carcinoma. *Br J Oral Maxillofac Surg* **2016**, *54*, 213–218, doi:10.1016/j.bjoms.2015.11.002.
49. Urban, D.; Gluck, I.; Pfeffer, M.R.; Symon, Z.; Lawrence, Y.R. Lymph Node Ratio Predicts the Benefit of Post-Operative Radiotherapy in Oral Cavity Cancer. *Radiother Oncol* **2013**, *106*, 74–79, doi:10.1016/j.radonc.2012.09.022.
50. Weckx, A.; Riekert, M.; Grandoch, A.; Schick, V.; Zöller, J.E.; Kreppel, M. Time to Recurrence and Patient Survival in Recurrent Oral Squamous Cell Carcinoma. *Oral Oncol* **2019**, *94*, 8–13, doi:10.1016/j.oraloncology.2019.05.002.

51. Xu, Q.S.; Wang, C.; Li, B.; Li, J.Z.; Mao, M.H.; Qin, L.Z.; Li, H.; Huang, X.; Han, Z.; Feng, Z. Prognostic Value of Pathologic Grade for Patients with Oral Squamous Cell Carcinoma. *Oral Dis* **2018**, *24*, 335–346, doi:10.1111/odi.12727.
52. Yamagata, K.; Fukuzawa, S.; Kanno, N.; Uchida, F.; Yanagawa, T.; Bukawa, H. Is Lymph Node Ratio a Prognostic Factor for Patients With Oral Squamous Cell Carcinoma? *J Oral Maxillofac Surg* **2019**, *77*, 1510–1519, doi:10.1016/j.joms.2019.01.037.
53. Zhao, T.-C.; Liang, S.-Y.; Ju, W.-T.; Fu, Y.; Zhou, Z.-H.; Wang, L.-Z.; Li, J.; Zhang, C.-P.; Zhang, Z.-Y.; Zhong, L.-P. High-Risk Lymph Node Ratio Predicts Worse Prognosis in Patients with Locally Advanced Oral Cancer. *J Oral Pathol Med* **2020**, *49*, 787–795, doi:10.1111/jop.13043.
